# Identity- versus effort-based bureaucratic discrimination among mobile European Union citizens: Evidence from conjoint experiments

**DOI:** 10.1177/14651165261423087

**Published:** 2026-03-04

**Authors:** Jana Gómez Díaz, Eva Thomann, Anita Manatschal, Xavier Fernández-i-Marín

**Affiliations:** 1 Department of Politics and Public Administration, University of Konstanz, Konstanz, Germany; 2 Swiss Forum for Migration and Population Studies, University of Neuchâtel, Neuchâtel, Switzerland; 3 Department of Political Science, Universitat de Barcelona, Barcelona, Spain

**Keywords:** Bureaucratic discrimination, migration, EU mobility, ethnic hierarchies, street-level bureaucracy, choice-based conjoint experiments

## Abstract

Discrimination by welfare bureaucrats in host countries poses significant administrative burdens for mobile European Union citizens’ social rights in practice. However, the relative importance of applicants’ identity and perceived effort therein is understudied. Combining discrimination and behavioral theory, we investigate how nationality and perceived effort affect bureaucratic discrimination. A choice-based conjoint survey experiment presents 2403 bureaucrats in Switzerland, Denmark, Ireland, and Spain with fictional information requests from French and Bulgarian citizens. The results of the experiment show that bureaucrats—particularly administrators with antimigration and right-wing attitudes—favor French citizens over Bulgarians. Preferential treatment was strongest for applicants whose language fluency and job-seeking activities indicate integration efforts. Bureaucratic discrimination is much more about what EU citizens do than who they are.

## Introduction

This article analyzes how the nationality and integration efforts of mobile European Union (EU) workers applying for welfare benefits influence their treatment by bureaucrats across European countries. The EU's labor mobility rules grant all EU citizens the same legal status, including the right to reside and seek employment freely in all member states, and guarantee equal access to social protections regardless of nationality. However, welfare chauvinism and interethnic competition over welfare as a scarce resource can heighten group tensions (Garand et al., 2017; Helbling and Kriesi, 2014; Hjorth, 2016; Van der Waal et al., 2013). Concerns over perceived “welfare tourism” can make migrants’ access to benefits politically contested (Fernández-i-Marín et al., 2021).

In response, member states may impose administrative burdens (Herd et al., 2023) or narrowly interpret EU social rights to limit nonnationals’ access (Heindlmaier and Blauberger, 2017; Martinsen et al., 2019; Thierry and Martinsen, 2018). Due to complex legal frameworks, mobile EU workers often seek guidance to navigate host countries’ administrative systems. Street-level bureaucrats, who implement welfare policy in direct interaction with mobile EU citizens (Lipsky, 1980/2010), play a key role in ensuring fair treatment. These bureaucrats have considerable discretion to either facilitate or obstruct access to benefits. When this discretion leads to disparities in waiting periods, information access, or welfare decisions, it may result in bureaucratic discrimination—biased behavior that systematically favors or disadvantages certain citizens, denying them equal treatment (Allport, 1954: 51; Correll et al., 2010; Einstein and Glick, 2017; Hemker and Rink, 2017).

Bureaucratic biases based on citizens’ ascriptive characteristics—such as gender, race, ethnicity, and religion—are well documented (Baumgartner et al., 2019; Fereidooni, 2011; Grohs et al., 2016; Knox et al., 2020; Thomann and Rapp, 2018; White et al., 2015). These traits, which are often unchangeable, reflect predetermined identities rather than achievements or merits. Discrimination theories explain how bureaucrats act on prejudices or stereotypes—whether implicit or explicit, taste-based, statistical, or shaped through socialization (Assouline et al., 2022; Correll et al., 2010; Koopmans et al., 2019; Schneider and Ingram, 1993). Research on migration attitudes and ethnic hierarchies provides rich international evidence on ethnic discrimination (Fibbi et al., 2021; Hagendoorn and Kleinpenning, 1991; Koopmans et al., 2019; Zschirnt and Ruedin, 2016), and suggests that perceived (dis)similarity of foreign EU citizens can trigger such biases as well (Lewicki, 2023; Rovny, 2024). As a result, EU citizens from more culturally distant countries are more likely to experience bureaucratic discrimination (Adam et al., 2021; Fernández-i-Marín et al., 2021; Thomann and Rapp, 2018).

Bureaucratic behavior is also shaped by broader societal, organizational, and national contexts (Borrelli, 2021; Einstein and Glick, 2017; Hemker and Rink, 2017; Terum et al., 2018). Behavioral public administration and street-level bureaucracy literature emphasize how overburdened bureaucrats make boundedly rational decisions, using mental shortcuts to prioritize clients (Knill et al., 2024; Lipsky, 1980/2010; Moseley and Thomann, 2021; Thomann et al., 2025). These decisions often reflect professional reasoning about applicants’ likely behavior or success and whether they justify investing limited administrative resources (Lipsky, 1980/2010; Møller, 2016). Research shows that welfare applicants’ efforts— “earned deservingness”—interact with ethnic markers to shape bureaucrats’ perceptions (Fekjaer et al., 2023; Jilke and Tummers, 2018; Lotta and Kirschbaum, 2021). Effort-based factors such as job-seeking and language proficiency, which signal integration, influence the administrative handling by welfare bureaucracies (Adam et al., 2021; Ager and Strang, 2008; Harder et al., 2018).

While existing research highlights the importance of both identity-based and effort-based deservingness, their relative weight in explaining bureaucratic discrimination remains unclear. To address this gap, we ask: how do mobile EU citizens’ identity (nationality) and integration efforts affect their treatment by bureaucrats abroad? Our theoretical contribution lies in assessing the relative influence of biases based on ascriptive traits versus “earned deservingness.” Clarifying which of these factors carries more weight helps situate bureaucratic behavior within its organizational context, supports the design of targeted policy interventions (Thomann et al., 2025), and empowers EU citizens in their welfare interactions.

Empirically, we broaden the scope of bureaucratic discrimination research, which often focuses on the United States, by examining a comparative European setting. Our study analyzes the treatment of French and Bulgarian EU citizens requesting information about welfare benefits. Using a choice-based conjoint survey experiment, we assessed 2403 bureaucrats across four countries with distinct administrative cultures (Peters, 2021): Spain, Denmark, Ireland, and Switzerland. Each participant evaluated requests from two fictitious EU citizens seeking information about welfare eligibility, with only one eligible to receive a detailed response. We varied nationality, language proficiency, and job-seeking efforts to detect discrimination triggers related to national origin and perceived integration. This approach helps identify bureaucratic discrimination as a key, and as our findings suggest, widespread issue in EU governance, extending beyond national or local levels to the street level of welfare implementation. Particularly amid rising bureaucratic burdens (Knill et al., 2024), this form of discrimination creates significant challenges for mobile EU citizens (Herd et al., 2023; Moynihan et al., 2015).

Our study combines innovative methods—Choice-Based Conjoint Analysis with Hierarchical Bayes (HB) estimation—to study discrimination in a conjoint design (Hein et al., 2022). Compared to traditional aggregate-level models, HB represents a step forward by not making the assumption of equal utilities for each individual. In this sense, they improve predictive validity and provide more flexible treatment of heterogeneity across groups and individuals (Fernández-i-Marín et al., 2021; Goeken et al., 2021; Halme and Kallio, 2011; Rossi et al., 2005; Sonnier et al., 2007; Villiger, 2022; Zhirkov, 2022).

The results reveal a consistent preference for French nationals over Bulgarian nationals. This effect is more pronounced among right-leaning bureaucrats and weaker among those with immigrant-friendly attitudes. However, bureaucrats most consistently prioritize applicants who they perceive to display integration efforts. These findings provide robust evidence of how identity and effort interact in shaping welfare discrimination and highlight the role of political ideology and attitudes. While professional reasoning partly drives these administrative behaviors, they challenge the realization of EU citizens’ social rights and introduce burdens in practice. Rather than being guided solely by ethnic prejudice, the bureaucrats in our study focus significantly on what mobile EU citizens do—more than on who they are.

## Theory and expectations

International migrants often face disadvantages not due to their capabilities, but because of prevailing ethnocultural stereotypes, prejudices, and institutional biases (Borrelli, 2021; Fibbi et al., 2021). While mobile EU citizens enjoy legal equality through freedom of movement and access to social rights, they remain subject to cultural stereotypes and preconceived ideas when interacting with host-country administrations. Although bureaucrats are expected to implement laws impartially (Albrow, 1997; Grohs et al., 2016), discretion in welfare implementation (Lipsky, 1980/2010) may lead to unequal treatment, which potentially undermines the principle of equal treatment before the law and threatens public trust, institutional legitimacy, and democratic norms (Adman and Jansson, 2017; Baumgartner et al., 2019; Epp et al., 2017; Zacka, 2019). It also risks weakening the EU's commitment to freedom of movement and equal access to social protections.

We focus on a critical yet understudied stage in the administrative process: when mobile EU citizens seek information about eligibility for welfare benefits. Street-level bureaucrats can ease or complicate this interaction. Differences in response rates, answer quality, or helpfulness can create administrative burdens, defined as the learning, compliance, and psychological costs citizens face when engaging with government (Moynihan et al., 2015). These burdens are particularly consequential for mobile EU citizens navigating legally complex, uncertain systems.

Research has extensively documented bureaucratic discrimination across domains such as policing, housing, employment, and asylum services (Adman and Jansson, 2017; Baumgartner et al., 2019; Flage, 2018; Grohs et al., 2016; Koopmans et al., 2019; Zschirnt and Ruedin, 2016). These patterns stem from a mix of individual-level prejudice, meso-level organizational settings, and macro-level contexts (Borrelli, 2021; Ceobanu and Escandell, 2010; Epp et al., 2017; Kende et al., 2022). Particularly identity-based discrimination may be statistical (based on aggregated experience), taste-based (driven by personal dislike), or shaped by socialization and policy cues (Allport, 1954; Arrow, 1998; Assouline et al., 2022; Becker, 1971; Schneider and Ingram, 1993).

Existing research largely either examines ascriptive, identity-based discrimination—how ethnicity, race or nationality shapes perceptions of deservingness (Hemker and Rink, 2017); or it highlights how effort-based indicators, such as job-seeking behavior or language proficiency, can influence welfare judgments (Jilke and Tummers, 2018; Lotta and Kirschbaum, 2021). However, few studies compare the *relative importance* of these identity and effort-based signals in shaping bureaucrats’ real-world behavior, especially in the EU context. Our study addresses this gap. We assess how ascriptive traits (nationality) and perceived integration efforts (language proficiency, job-seeking efforts) influence bureaucratic responses to welfare inquiries in the context of intra EU mobility. Our study also examines how these effects vary with bureaucrats’ political ideology and attitudes toward immigration.

### Ascriptive traits: Nationality-based discrimination

In Europe, discrimination often reflects national and ethnic origins rather than racial identity, as is more common in the United States (Bell, 2009; Eide, 1998). Post–World War II immigration increased ethnic and racial diversity and led to persistent inequalities in housing, employment, and public services (Fibbi et al., 2021). Many of these disadvantages—so-called “ethnic penalties”—cannot be explained by differences in human capital alone (Borgna and Contini, 2014; Heath and Cheung, 2007). Instead, they stem from structural exclusion and both subtle and overt discrimination. Discrimination in welfare delivery is especially salient, as social benefits are limited resources. Social identity theory and research on welfare chauvinism suggest that people are more likely to favor in-group members when resources are scarce (Hjorth, 2016; Tajfel, 1982). Accordingly, street-level bureaucrats may see nonnationals as less deserving, leading to less friendly treatment, lower-quality information, or additional administrative hurdles (Adman and Jansson, 2017; Hemker and Rink, 2017; Thomann and Rapp, 2018).

However, this dynamic becomes more complex when comparing different EU citizens—who all *legally* belong to the same supranational “ingroup.” Here, ethnic hierarchies and perceived cultural distance come into play. Research shows that individuals judge out-groups based on their perceived similarity to the in-group, creating informal hierarchies of more or less “acceptable” migrants (Hagendoorn, 1993; Snellman and Ekehammar, 2005). These hierarchies are shaped by cultural stereotypes, historical narratives, and structural inequalities (Hagendoorn and Kleinpenning, 1991; Helbling and Kriesi, 2014; Koopmans et al., 2019). Western European countries often perceive Eastern European nationals as more culturally and economically distant (Lewicki, 2023; Rovny, 2024). Prior research confirms that such perceptions shape bureaucratic behavior—for example, in Germany, Eastern European applicants with limited language skills receive lower-quality service (Adam et al., 2021; Fernández-i-Marín et al., 2021).

*H1:* Information requests from EU mobile workers from France are more likely to be prioritized than those from Bulgaria.

These biases may further depend on bureaucrats’ individual ideologies and attitudes. Beyond average effects, we expect ideology and immigration attitudes to condition how strongly nationality shapes discretionary choices. Research consistently shows that right-wing and nativist orientations are often associated with stronger preference for cultural homogeneity, greater skepticism toward immigration, and heightened sensitivity to perceived out-group threats (Green et al., 2011; Manatschal and Rapp, 2015). Such beliefs can amplify the salience of nationality as a social cue, making right-wing/nativist bureaucrats more likely to differentiate between applicants based on perceived group membership and cultural or economic distance from the host society (Ceobanu and Escandell, 2010; Hainmueller and Hiscox, 2007). This logic aligns with social identity theory and stereotype activation research (Devine, 1989; Tajfel and Turner, 1979), which together suggest that preexisting beliefs about group boundaries and norms condition how decision-makers evaluate otherwise similar applicants.

Research documents that the core ideology of political conservatism, including right-wing populism, stresses resistance to change, fear of threat and loss, and a desire for order, structure and closure (Jost et al., 2003; Manatschal and Rapp, 2015). Right-wing/nativist bureaucrats could therefore be more likely to weight nationality signals higher when prioritizing cases, especially for applicants perceived as culturally/economically distant and therefore potentially threatening. By contrast, left-leaning voters endorse egalitarian norms (Bonilla et al., 2022; Mendelberg, 2001), which is why we expect left-leaning or proimmigrant bureaucrats to show a stronger internal motivation to control prejudice and to individuate rather than categorize. However, recent evidence suggests that this pattern is not uniform: Rueß et al. (2025) show that egalitarian attitudes do not always translate into reduced bias in bureaucratic decision making. Taken together, party ideology and immigration attitudes should *moderate* nationality effects by shaping the salience and acceptability of identity-based heuristics in bureaucratic judgment.

*H1a:* Discrimination based on nationality is stronger among bureaucrats with right-wing political ideologies.

*H1b:* Discrimination based on nationality is stronger among bureaucrats holding exclusive immigration attitudes.

### Effort-based deservingness

While much of the existing research emphasizes who clients are—focusing on ascriptive characteristics such as nationality or ethnicity—what clients do can also shape how street-level bureaucrats treat them. This distinction matters because welfare policy delivery takes place in environments marked by significant resource and time constraints (Knill et al., 2024). Therefore, bureaucrats are routinely forced to make imperfect, boundedly rational prioritization decisions (Lipsky, 1980/2010). To manage these constraints, they have to resort to mental shortcuts (Moseley and Thomann, 2021) and often rely on professional reasoning to apply “meaningful” criteria for distinguishing among clients. For instance, they may perceive certain applicants as more deserving because they appear hardworking, needy, or likely to succeed (Jilke and Tummers, 2018; Lipsky, 1980/2010; Lotta and Kirschbaum, 2021; Møller, 2016). In the context of EU mobility, such reasoning may center on the applicant's efforts to integrate—through language acquisition or engagement with the labor market. Importantly, these decisions, while based on cognitive heuristics, do not necessarily stem from personal bias or prejudice (Moseley and Thomann, 2021). Rather, they may reflect pragmatic, professional coping mechanisms aimed at allocating limited time and administrative resources efficiently.

### Language skills

Existing literature has identified language proficiency as a key signal of integration and, consequently, a factor in how bureaucrats perceive and treat welfare applicants (Jilke and Tummers, 2018). Individuals with fluent language skills are better positioned to form social connections, access the labor market, and develop a sense of belonging in the host country. As such, language skills are widely considered one of the most important indicators of successful immigrant integration (Ager and Strang, 2008; Harder et al., 2018; Pecoraro et al., 2022).

Prior research suggests that applicants with strong language skills are favored over those with limited proficiency (Adam et al., 2021). This is partly because bureaucrats interpret language proficiency as a proxy for the applicant's overall competence, motivation, and potential for successful integration (Holzinger, 2020; Scheibelhofer et al., 2021; Tenzer et al., 2014). As a result, they may regard such applicants as more trustworthy and deserving of assistance (Holzinger, 2020). Conversely, language barriers not only challenge applicants’ ability to navigate the system but also increase the burden on bureaucrats, who may have to invest more time and effort in communication. To minimize these costs, bureaucrats may prioritize applicants with better language skills, thereby streamlining interactions, avoiding misunderstandings, and reducing administrative complexity (Eckhard and Friedrich, 2024; Holzinger, 2020).

*H2:* Information requests from applicants with fluent language skills are more likely to be prioritized than those from applicants with broken language skills.

## Labor market integration efforts

The literature on street-level bureaucracy has long emphasized the role of deservingness cues in shaping how welfare workers evaluate and interact with clients (Lipsky, 1980/2010; Lotta and Kirschbaum, 2021; Møller, 2016; Thomann and Rapp, 2018). These cues stem not only from clients’ identities but also from their behaviors. A key concept here is earned deservingness: bureaucrats may view clients as more deserving when they demonstrate effort, such as through job-seeking or other forms of engagement (Jilke and Tummers, 2018). This contrasts with identity-based deservingness, which relies on ascriptive characteristics. While the two can overlap, earned deservingness provides a behavior-based lens through which bureaucrats evaluate applicants. The context in which bureaucrats operate also influences which form of deservingness takes precedence (Lotta and Kirschbaum, 2021).

Empirical studies show that street-level bureaucrats often prioritize clients they perceive as hardworking or proactive (Jilke and Tummers, 2018). Building on this, we expect that applicants who are perceived to have made visible efforts to seek employment before requesting welfare support will be perceived as more deserving and will receive more favorable treatment.

*H3:* Information requests from applicants who have made job-seeking efforts are more likely to be prioritized than those from applicants who have not.

## Data and methods

This study examines information requests relating to access to social assistance benefits for unemployed job-seeking EU citizens. To test the effects of the nationality and integration effort of mobile EU citizens who seek to apply for welfare benefits on how they are treated by bureaucrats, we conducted identical conjoint survey experiments in Switzerland, Denmark, Ireland, and Spain. The data was collected through 15-minute surveys targeting 2403 bureaucrats in the four countries.^1^ The sample includes an oversample of bureaucrats as part of a larger (representative) survey which was conducted among general population respondents in the four countries. The Online appendix contains descriptive statistics of the respective samples. Since the composition of the universe of all bureaucrats per country is unknown, we cannot assess the representativeness of our samples.

Our samples include civil servants and other public service staff, as their positions involve carrying out administrative functions within the state apparatus. We excluded teachers, university faculty, and police officers, as their professional duties differ markedly from the kinds of administrative decision-making processes we are interested in. Bureaucrats in our samples work in administrative settings, where applying rules, interpreting regulations, and managing citizen-facing procedures form part of their role. While our respondents are not, for the most part, street-level officials responsible for assessing mobile EU citizens’ welfare claims, they operate within the same overarching institutional culture, guided by the principles of impartiality, legality, and service to the public. This makes them a more suitable proxy for welfare decision-makers than the general public, who lack bureaucratic experience. Recruiting a sufficiently large number of welfare-facing street-level bureaucrats proved not feasible for this experiment. This is likely due to the high workload and time constraints characteristic of these roles. Such workload pressures are themselves relevant to our argument, as they may increase the likelihood of the kind of imperfect decision-making patterns observed in our experiment.

In spite of these practical recruitment limits, our sampling strategy offers a clear advantage over much existing research on bureaucratic discrimination, which frequently relies on general population samples to simulate bureaucratic decision-making (Adam et al., 2021). By drawing on respondents embedded in the public administration, our study benefits from greater internal validity, as these individuals are accustomed to the procedural constraints, institutional norms, and professional expectations of bureaucratic contexts that are relevant here.

We included EU member countries and a country that has freedom of movement arrangements with the EU. Within this group, we selected “diverse cases” representing four different administrative contexts: Spain (Southern Napoleonic), Denmark (Nordic), Ireland (Anglo-Saxon), and Switzerland (Federal) (Peters, 2021). By excluding countries with low immigration or xenophobia levels, we focus on countries with a comparatively higher likelihood of bureaucratic discrimination occurring (Levy, 2008). This allows us to test the external validity of our theoretical arguments across very different administrative contexts. Our samples in the four countries consists of public service employees and civil servants, excluding teachers/professors and police officers.

Ethics clearance for this research was provided by the ethics commission at the University of Neuchâtel, Switzerland (CER-UNINE, réf. 109-2023).^2^

It contains a choice-based conjoint experiment to measure bureaucratic discrimination, standard socioeconomic and demographic questions. Participants were asked to take on the role of a local government official, faced with two information requests from fictitious mobile EU citizens inquiring about their eligibility for welfare benefits. We emphasized that the conditions granting these benefits are complex, making a detailed and personalized response highly valuable to both individuals. However, the participants could only provide such assistance to one of them.

In our experiment, respondents were asked to choose between two hypothetical applicants rather than given the option to refuse both. This design choice mirrors the operational reality of many public administration settings, particularly in the context of bureaucratic overload. In such conditions, officials frequently face “triage” decisions—prioritizing one client's case over another due to time and resource constraints (Knill et al., 2024). While actual frontline bureaucrats may also decide not to process certain cases at all, the daily reality in many administrative contexts involves making comparative judgments under pressure. By forcing a choice, our design captures this core feature of decision-making in overloaded bureaucracies and allows us to isolate how nationality and perceived effort influence prioritization between applicants.

As research shows, several (combinations of) attributes of EU citizens can trigger discrimination, such as the perceived integration of particular groups, their conformity with the prevailing culture, and their general phenotypical “otherness” (Hagendoorn and Kleinpenning, 1991). Therefore, identity- and effort-based factors potentially triggering bureaucratic discrimination, such as nationality (French vs. Bulgarian), language proficiency (limited vs. fluent) and number of job applications (0 vs. 5) were varied randomly. The latter two capture integration effort, whereas French versus Bulgarian reflects perceived similar versus dissimilar national origin. Importantly, at the time of our study, French and Bulgarian citizens enjoyed the same legal rights with regard to residence and access to social welfare in other EU Member States under EU free movement law. These rights stem from EU citizenship and the principle of equal treatment laid down in Directive 2004/38/EC and subsequent case law, and they had applied uniformly to Bulgarian and French citizens for more than a decade at the time of data collection (European Commission, 2014). While Bulgaria was not yet fully integrated into the Schengen area at that point, Schengen membership concerns the removal of internal border controls and does not affect EU citizens’ rights to residence or access to social benefits, which apply independently of Schengen status. In addition, our profiles vary applicants’ gender (male vs. female), age (25 vs. 55), previous profession (salesperson vs. medical doctor) and duration of stay in Switzerland (1 vs. 4 years). Table 1 displays the conjoint design for Switzerland, and the Online appendix provides full operationalizations.

**Table 1.** Example conjoint design (Switzerland).

Please consider a scenario in which you work for the local government. you receive two information requests from two EU citizens who have already worked in your country and who ask about their eligibility to claim social assistance (*Sozialhilfe*). The conditions under which individuals without a Swiss passport can access this benefit are rather complex. A detailed and personalized answer that takes into account the specific situation of the individual as described in the request would help both individuals greatly. But you only have the time to provide one of the individuals with personalized assistance. The other will get a standardized email providing general information.		
	**Individual 1**	**Individual 2**
**Nationality**	French	Bulgarian
**Gender**	Female	Male
**Age**	25	55
**Previous profession**	Salesperson	Medical doctor
**Proficiency in either** **German, French, Italian, or Romansh**	Limited	Fluent
**Duration of stay (in Switzerland)**	4 Years	1 Year
**Job applications submitted last month**	0	5

In this study, discrimination captures preferential treatment of individual applicants, where we expect bureaucrats to be more likely to provide French citizens with personalized assistance than Bulgarian nationals. The study relies on a Choice-Based Conjoint Analysis with HB estimation to explore discriminatory behaviors among respondents (Fernández-i-Marín et al., 2021; Hainmueller et al., 2015). Our methodology assumes stability and no carryover effects, with profiles randomized accordingly. Choice-Based Conjoint Analysis with HB models offers several benefits over simple conjoint analysis. It is known for its enhanced predictive validity (Hein et al., 2022) and provides more precise estimates of individual-level preferences by utilizing information across respondents, resulting in robust and accurate outcomes, particularly when individual-level data is sparse. It further allows for the consideration of preference heterogeneity across different groups or segments, as it is done in this study, providing a deeper understanding of how preferences differ across various contexts or populations (Fernández-i-Marín et al., 2021; Halme and Kallio, 2011; Rossi et al., 2005; Sonnier et al., 2007; Villiger, 2022).

## Results

We analyze a potential bias that favors French citizens over Bulgarian citizens, and how effort-based factors influence bureaucrats’ treatment of applicants’ requests. In a traditional aggregate-level model the assumption is that all individuals share the same utilities, and the estimation of effects for different groups is performed using interaction terms or by running models by subgroups. Instead, the HB approach provides a more flexible approach where the first step is the estimation of utilities for each individual and each feature, generating a total of 19,224 parameters (2403 individuals × seven features in the design + one technical control for first shown vignette). This first step gives us estimations of discrimination in all features for each of the respondents. The second stage involves assessing whether individual-level variables (ideology, attitudes) are able to explain differences in the individual utilities, by usually comparing averages for each group, in a simple comparison of means. However, in both cases—traditional aggregated-level models and HB—we can report results in Average Marginal Component Effects (which we report in the Online appendix).

**Figure 1.**
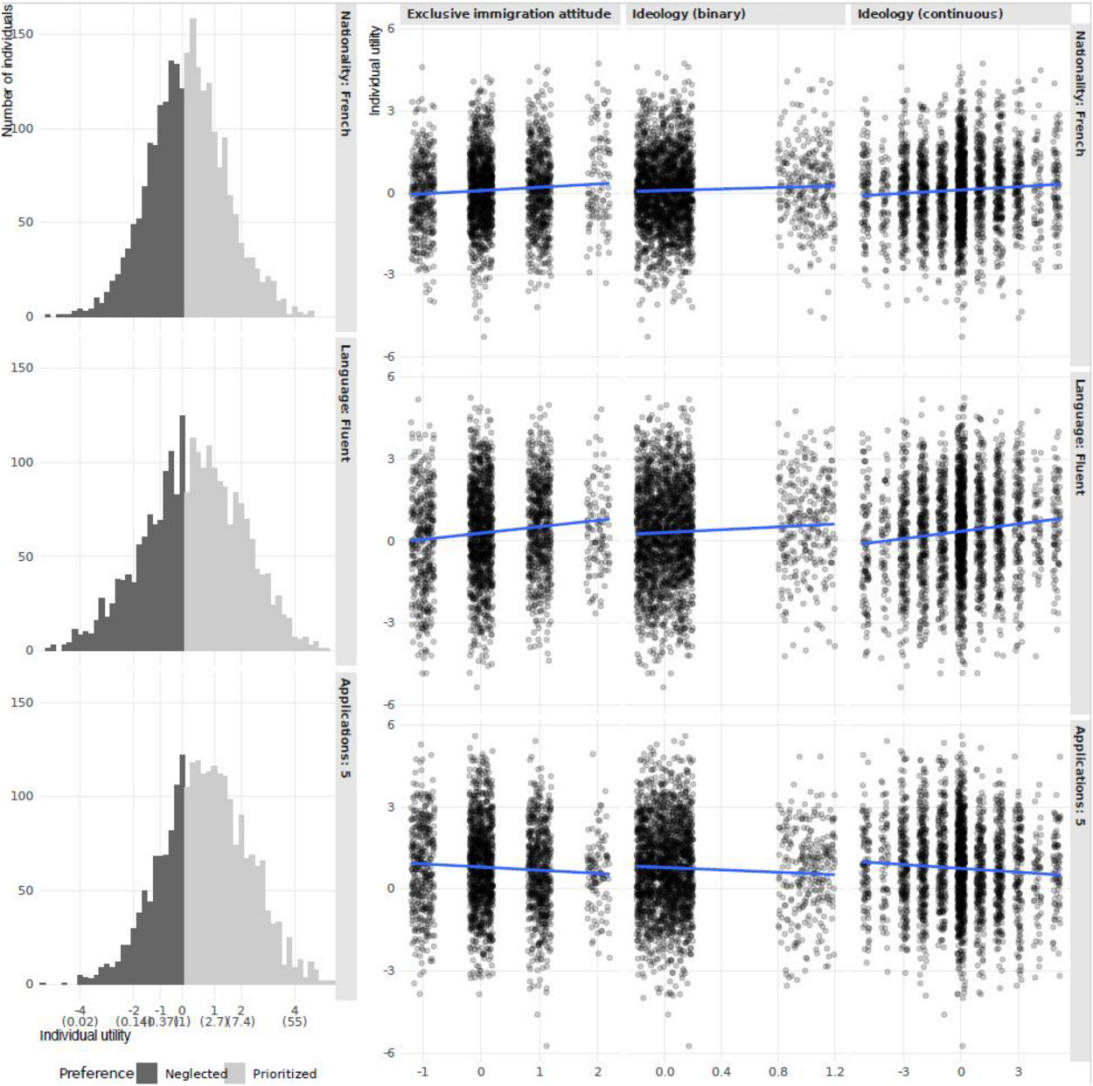
(a) (left). Histograms of part-worth utilities of each individual and (b) (right): Correlations between utilities and individual-level variables.

*Note:* Figure 1(a) shows histograms displaying the part-worth individual utilities (namely, the preferences of each surveyed individual towards prioritized or neglected treatment in each of the three features considered. **Figure 1(b)** shows correlations of the same individual utilities on the vertical axis (*y*) against three different individual-level variables (ideology and exclusive immigration attitude), dots are jittered for better visualization. Smoothed lines have been included. *N* (Ireland) = 600, *N* (Denmark) = 655, *N* (Spain) = 648, *N* (Switzerland) = 500.

In Figure 1(a), we provide the most basic and general representation of the findings, which is the histograms of the distributions of the part-worth utilities of each individual; namely, whether each individual favors a specific feature or not, and by how much. The horizontal axis is shown both in terms of part-worth utilities (logged odds) and odds (in parenthesis). Figure 1(b) represents the second stage of the analysis, where an individual-level variable (horizontal axis) is correlated with the utility (discrimination, vertical axis).

### Discrimination based on nationality

Our first hypothesis (H1) posits that bureaucrats would preferentially treat French over Bulgarian applicants. The results support this hypothesis. On average, French citizens are 10% (95% CI [3% to 16%]) more likely to receive favorable treatment compared to Bulgarian citizens (see the Online appendix).

The significant variation in individual “utilities,” as shown in our analysis, indicates that while some bureaucrats are strongly biased toward French citizens, others might even show a slight preference for Bulgarians (Figure 1(a), top row). Nevertheless, the overall trend of favoritism toward French nationals is clear, supporting the idea that discrimination is prevalent, even if it varies across individuals. These results lend support to H1, and they are consistent with previous research suggesting that individuals from culturally and economically more similar countries are often preferred in bureaucratic, and more generally in political and societal contexts (Fernández-i-Marín et al., 2021; Helbling and Kriesi, 2014; Koopmans et al., 2019).

## Influence of ideology and immigration attitudes

The first subhypothesis (H1a) examines whether right-wing ideology exacerbates discrimination against Bulgarian nationals. Findings corroborate this hypothesis. We operationalize ideology in two ways: (1) “Ideology (continuous)” considers ideology as continuous, centered at 5 with higher values moving to the right spectrum of ideology; and (2) “Ideology (binary)” is a dummy variable with 1 indicating a right-wind ideology (a 7 or more in the original 0 to 10 scale). We report the results both in tabular (Table 2) and visual (Figure 1(a) and (b) formats.

**Table 2.** Simple linear regression models.

		Ideology (continuous)	Ideology (binary)	Exclusive immigration attitude
**(Intercept)**		0.11	0.08	0.08
		[0.06, 0.17]	[0.02, 0.14]	[0.02, 0.14]
**Ideology (continuous)**		0.04		
		[0.02, 0.07]		
**Ideology (binary)**			0.19	
			[0.02, 0.37]	
**Exclusive immigration attitude**				0.14
				[0.06, 0.21]
**Num.Obs.**		2403	2403	2243
**R^2^**		0.004	0.002	0.006
**Log.Lik.**		−4248.999	−4251.724	−3964.493

*N*ote: Simple linear regression models on individual discriminations preferring French over Bulgarian profiles. Ninety-five percent credible intervals between square brackets. Each model containing one single variable. The model with a binary variable (Ideology (binary)) can be understood as a simple comparison of means. *N* (Ireland) = 600, *N* (Denmark) = 655, *N* (Spain) = 648, *N* (Switzerland) = 500.

**Figure 2.**
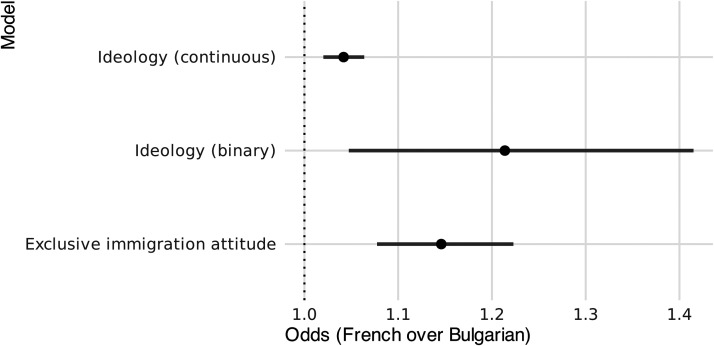
Effect of each of the three individual variables on the odds of prioritizing French over Bulgarian profiles.

*Note*: The dot represents the median of the posterior density, and the horizontal line covers the 95% credible interval. *N* (Ireland) = 600, *N* (Denmark) = 655, *N* (Spain) = 648, *N* (Switzerland) = 500.

Per Table 2 and Figure 2, ideological orientation significantly influences bureaucratic behavior, with individuals on the far-right spectrum being more likely to favor French over Bulgarian citizens. Both general and explicitly right-wing ideological orientations are significant predictors of preferential treatment for French over Bulgarian applicants. Concretely, a one-unit increase on the ideological scale to the right is associated with an increase in the likelihood of favoring French over Bulgarian by 4.2% (exp(0.041)), and the binary self-positioning on the far- and extreme-right spectrum of ideology increases the likelihood of favoring French over Bulgarian by 21% (exp(0.194)). This implies that conservative or nationalist ideologies play a role in discriminatory preferences, confirming H1a. This is further consistent with existing literature that links nationalist norms and conservative ideologies with a higher propensity for discriminatory practices (Villiger, 2022).

The second subhypothesis (H1b) tests whether antiimmigration attitudes reinforce discrimination against Bulgarian nationals. In line with our expectation, our analysis revealed that an exclusive attitude toward immigration has an effect. Bureaucrats who are more closed to immigration are more likely to discriminate against Bulgarian nationals, when compared to bureaucrats who prefer limiting immigration. An exclusive immigration attitude is associated with a preference for French applicants compared to Bulgarian applicants. Concretely, there was a 14% (exp(0.136) increase for each of the three categories of the variable. This suggests that individuals who support more exclusive immigration policies are more likely to display national-origin-based favoritism in their preferences. These findings support Hypothesis 1b.

## The role of effort in reducing discrimination

**Figure 3.**
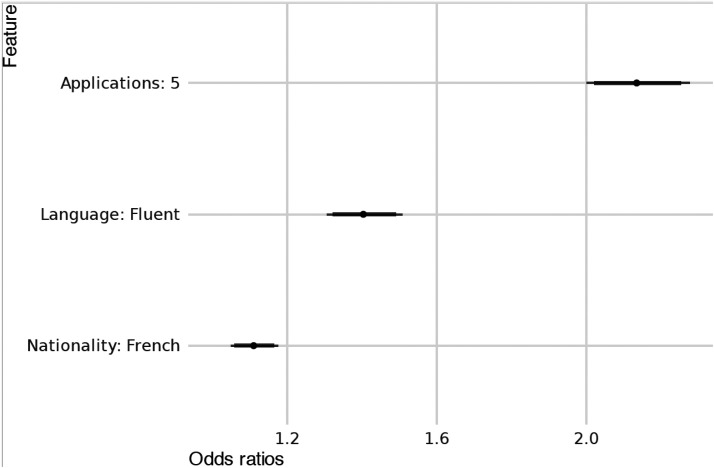
Effects of nationality and integration effort on the odds of prioritization.

*Note*: The dot represents the median of the posterior density, and the horizontal line covers the 95% credible interval. *N* (Ireland) = 600, *N* (Denmark) = 655, *N* (Spain) = 648, *N* (Switzerland) = 500.

Our second and third hypotheses explore whether increased effort, as indicated by factors such as language proficiency (H2) and the number of job applications (H3), could reduce discrimination. Impressively, the findings clearly exceed the estimates for nationality-based discrimination, suggesting that effort is a more relevant trigger for discrimination than nationality in the context of EU mobility. Regarding integration effort, language proficiency emerges as a significant marker, with bureaucrats on average being 40% (95% CI [31% to 51%]) more likely to favor individuals with fluent language skills, regardless of their nationality (see Figure 3 and the Online appendix). The effect of client effort for labor market integration, as measured by the number of job applications, is even more pronounced, with a 113% increase in the likelihood of receiving favorable treatment (or 2.13 times more likely, 95% CI [2.0 to 2.28], see Figure 3 and the Online appendix). However, while fluency is a marker of integration effort, a low number of applications may indicate problems regarding employability. Theoretically, low employability would indicate “needed deservingness,” which is empirically linked with positive rather than negative discrimination (Jilke and Tummers, 2018). Therefore, we consider it more plausible that our findings reflect perceived earned deservingness.

These findings lend strong support to Hypotheses 2 and 3. They highlight that while identity (i.e. nationality) is a trigger of bureaucratic decision-making, effort-related factors have an even stronger influence on administrative welfare decisions.

## Discussion

Our analysis of the effects of mobile EU citizens’ nationality and perceived efforts on their treatment by bureaucrats across different European countries produces three key findings.

## Key findings

First, *nationality matters*: not all EU citizens are treated the same. We find evidence for nationality-based discrimination, where bureaucrats systematically prefer French over Bulgarian nationals. This aligns with previous findings on the role of perceived cultural and economic similarity in shaping discriminatory behavior based on constructed ethnic hierarchies (Adam et al., 2021; Fernández-i-Marín et al., 2021; Helbling and Kriesi, 2014; Koopmans et al., 2019). The fact that French citizens were 10% more likely to receive favorable treatment suggests that even under the legal framework of equal EU citizenship and within the seemingly neutral bureaucratic sphere, nationality-based biases persist. These are likely influenced by the perceived cultural and economic distance of applicants and their phenotypical otherness. These disadvantages can be subtle, such as differential treatment in terms of the responsiveness and quality of service provided. Yet, nationality-based bureaucratic discrimination potentially violates both EU law and most national constitutions.

Second, we find that far from being a technical, neutral process, *the street-level implementation of EU social rights is politicized*. Bureaucrats with right-wing ideologies were significantly more likely to favor French citizens over Bulgarians, demonstrating how nationalist norms and conservative ideologies contribute to discriminatory practices. Conversely, those with more open attitudes towards immigration were less likely to discriminate. These findings are in line with the broader literature on migration attitudes (Ceobanu and Escandell, 2010; Green et al., 2011; Hainmueller and Hiscox, 2007; Kende et al., 2022; Meuleman et al., 2009) and highlight the impact of individual socio-political beliefs on bureaucratic behavior. Bureaucrats are people, after all (Adam et al., 2021)—and by implication, welfare policy delivery is a continuation of the politics surrounding EU integration “on the ground.”

Third, we find that *client effort matters more than client identity*. Findings confirm that effort-related factors significantly influence bureaucratic decision-making—and more so than identity factors. This is in line with previous research on street-level bureaucracy, which indicates that street-level bureaucrats who have no choice but to prioritize first and foremost apply professional reasoning based on notions of deservingness that they derive from clients’ efforts (Fekjaer et al., 2023; Jilke and Tummers, 2018; Lotta and Kirschbaum, 2021). We consider this finding to be a double-edged sword. On the one hand, there is something empowering about the fact that mobile EU citizens can “earn” their deservingness through making efforts. On the other hand, if bureaucrats unconsciously or consciously reward those who they perceive as making a better effort by providing better service to them, it has complex implications. In practice, it can create administrative burdens especially for those client groups with lower social capital who are less able to integrate but might be most in need of assistance (Herd et al., 2023; Moynihan et al., 2015).

## Robustness test and limitations

To test the external validity of our experimental findings, we conducted separate national analyses for each country (see the Online appendix) that allow us to assess the results’ generalizability across diverse national contexts. While the overall trends align with the presented findings, these analyses reveal some important nuances. For the “Exclusive immigration attitude” variable, the aggregated result across all countries (“all”) represents a summary of the individual country estimates, showcasing consistency. However, for the two ideological variables, the overall effect is greater than the estimates obtained from individual countries. This suggests that the general trends are robust when considering bureaucrats as a single group, but variations across countries reveal that bureaucrats differ significantly in their ideological and attitudinal composition. While the direction of results remains consistent across countries (see Online appendix), the variability underscores the need to interpret findings with caution.

This cross-country analysis underscores the challenges of generalizing findings in multinational studies. While our aggregated results are robust and align with theoretical expectations, country-level variations reveal the heterogeneous nature of bureaucratic populations, offering nuanced insights. The Online appendix provides descriptive statistics that shed light on cross-country variability. For instance, Denmark and Spain have twice as many right-leaning (8 or higher on a 10-point scale) respondents as Ireland. However, Spanish respondents *on average* exhibit a more left-leaning ideology and greater openness toward immigrants than Denmark or Switzerland. These differences may result either from sampling strategies or intrinsic differences in bureaucratic populations. Sampling biases, if present, cannot be retroactively corrected, while national differences emphasize the importance of context in cross-country analyses.

Our bureaucrat samples were oversamples from larger, representative surveys of the general population in each country. However, they are not representative of all bureaucrats within their countries, as no data exists on the full composition of these groups. This highlights the need for better access to register data to create representative bureaucrat samples in surveys.

Another limitation of the study is that our respondents are not the exact subset of street-level officials tasked with evaluating welfare claims from mobile EU citizens. Engaging such officials in research of this kind is notoriously challenging. This likely reflects the heavy workloads and structural pressures typical in these roles. Such constraints are themselves relevant to our argument, as they may heighten the likelihood of the kind of time-pressured, imperfect decision-making observed here, suggesting that the results reported here are conservative estimates.

Nevertheless, the fact that all our respondents are employed in the public administration and bound by the same professional norms of impartiality, legality, and procedural fairness, means they are far closer in orientation and experience to welfare bureaucrats than members of the general public, who often lack direct exposure to bureaucratic processes. This enhances the internal validity of our findings relative to studies relying on citizen samples, where assumptions must be made about how nonbureaucrats would behave in administrative settings. The choice of this sample thus represents a pragmatic but defensible balance between ideal case targeting and feasible recruitment in multicountry experimental research. Improved data access would enable researchers to provide clarity on the matter and construct representative bureaucrat samples to address these challenges. Despite these limitations, our results have enhanced internal validity as they are based on a sample of actual bureaucrats.

## Conclusions

Our study contributes to the growing body of literature on bureaucratic discrimination (Adam et al., 2021; Fernández-i-Marín et al., 2021; Hemker and Rink, 2017; Thomann and Rapp, 2018) by providing robust evidence on the so far neglected *relative* importance of different types of bias, ascriptive (nationality based) versus effort-based (language skills and job-seeking efforts), in explaining bureaucratic discrimination. For the context of mobile EU citizens seeking information about welfare eligibility, we find—as expected—that the bureaucrats in our four countries display both types of biases. They preferentially treat French over Bulgarian requests, as well as those from fluent speakers and individuals showing labor market integration effort. However, our results reveal that effort-based discrimination is much more substantial than nationality-based discrimination. Bureaucrats in our study focus significantly on what mobile EU citizens do—more than on who they are. These findings have broader social and political implications for intra-EU mobility and integration in Europe (Heath and Cheung, 2007).

First, the preferential bureaucratic treatment of certain EU nationalities indicates incomplete EU integration at the street-level (Adam et al., 2021; Gago, 2021). It can contribute to tensions between member states and perceptions that the promise of equal EU citizenship is a false one in practice (Heath and Cheung, 2007; Heindlmaier and Blauberger, 2017; Thierry and Martinsen, 2018). Among those who are systematically disadvantaged, it can produce feelings of resentment and alienation and hinder their ability to integrate and achieve economic stability (OECD, 2025; Platt et al., 2021). Second, our results underscore the importance of considering the socio-political context in which bureaucrats operate (Borrelli, 2021; Epp et al., 2017; Scheibelhofer et al., 2021). The fact that bureaucrats’ ideology and attitudes toward migration influence how they treat mobile EU citizens may have implications for public accountability and the adoption of interventions to reduce bureaucratic discrimination (Thomann et al., 2025), which may need to be tailored to address the bureaucrats’ specific ideological and attitudinal profiles.

Third, the finding that bureaucrats prioritize clients based on what they do much more than on who they are begs for a normative appraisal which is difficult to provide. Contrary to nationality-based discrimination, this form of street-level coping is not in obvious violation of legal norms. It moreover demonstrates that street-level bureaucrats are not just bias-prone discriminators in need of disciplining, but professionals who take discretionary decisions under pressure to help clients (Jilke and Tummers, 2018). Indeed, street-level bureaucracy theory has always stressed that the ideal of identical treatment of all clients cannot be realized in bureaucratic practice characterized by inevitable resource and time constraints (Lipsky, 1980/2010). Simultaneously, the preferential treatment of applicants who demonstrate effort has significant implications for those vulnerable mobile EU citizens who may face language barriers, lack local networks, or encounter structural disadvantages in the labor market (Arnholtz and Leschke, 2023). For them, the complexity of welfare systems can be particularly challenging (OECD, 2025; Platt et al., 2021). The lack of tailored support from bureaucrats exacerbates administrative burden (Herd et al., 2023; Moynihan et al., 2015) that penalizes them for not conforming to the expected norms of effort, potentially leading to a cycle of exclusion and disadvantage.

These finding thus suggest fruitful areas for further research in three particular areas. First, more research is needed on the longer-term impacts of bureaucratic discrimination on the social and economic integration of mobile EU citizens (OECD, 2025). Second, further investigation into the effectiveness of targeted antidiscrimination interventions in different national contexts would provide valuable insights for policymakers and practitioners aiming to create more equitable public service (Moseley and Thomann, 2021; Thomann et al., 2025). Third, future research should test if the pattern that effort-based discrimination appears more pronounced than ethnic discrimination extends to the context of international migration, too. Research suggests that ethnic discrimination is more likely when it comes to international migrants, as opposed to European migrants or mobile EU citizens (Hemker and Rink, 2017; Thomann and Rapp, 2018;). Finally, future research should explore the complex interplay between national context, individual-level factors, and bureaucratic behavior. Specifically, more cross-national comparative studies are needed to better understand how different sociopolitical environments shape discriminatory practices in public administration.

## Supplemental Material

sj-docx-1-eup-10.1177_14651165261423087 - Supplemental material for Identity- versus effort-based bureaucratic discrimination among mobile European Union citizens: Evidence from conjoint experiments Supplemental material, sj-docx-1-eup-10.1177_14651165261423087 for Identity- versus effort-based bureaucratic discrimination among mobile European Union citizens: Evidence from conjoint experiments by Jana Gómez Díaz, Eva Thomann, Anita Manatschal and Xavier Fernández-i-Marín in European Union Politics

sj-zip-2-eup-10.1177_14651165261423087 - Supplemental material for Identity- versus effort-based bureaucratic discrimination among mobile European Union citizens: Evidence from conjoint experiments Supplemental material, sj-zip-2-eup-10.1177_14651165261423087 for Identity- versus effort-based bureaucratic discrimination among mobile European Union citizens: Evidence from conjoint experiments by Jana Gómez Díaz, Eva Thomann, Anita Manatschal and Xavier Fernández-i-Marín in European Union Politics

sj-zip-3-eup-10.1177_14651165261423087 - Supplemental material for Identity- versus effort-based bureaucratic discrimination among mobile European Union citizens: Evidence from conjoint experiments Supplemental material, sj-zip-3-eup-10.1177_14651165261423087 for Identity- versus effort-based bureaucratic discrimination among mobile European Union citizens: Evidence from conjoint experiments by Jana Gómez Díaz, Eva Thomann, Anita Manatschal and Xavier Fernández-i-Marín in European Union Politics

## Notes

1. Specifically, 600 bureaucrats from Ireland, 655 from Denmark, 648 from Spain and 500 from Switzerland.

2. The study was preregistered on AsPredicted (#149527, https://aspredicted.org/b3zv-kyhs.pdf). Preregistration details, as well as the full preregistration PDF are shown in the Online appendix.
