# Supplementary material for: Identity- versus effort-based bureaucratic discrimination among mobile European Union citizens: Evidence from conjoint experiments
Source: Eur Union Polit. 2026 Mar 4;27(2):301–24. doi: 10.1177/14651165261423087 (PMC13218594; doi:10.1177/14651165261423087)
Supplement: sj-docx-1-eup-10.1177_14651165261423087 - Supplemental material for Identity- versus effort-based bureaucratic discrimination among mobile European Union citizens: Evidence from conjoint experiments [file sj-docx-1-eup-10.1177_14651165261423087.docx]

# Online Appendix

**Identity- versus effort-based bureaucratic discrimination among mobile European Union citizens: Evidence from conjoint experiments**

Jana Gómez Díaz, Eva Thomann, Anita Manatschal and Xavier Fernández-i-Marín

Table of Contents

[Appendix 1: Operationalization, Descriptive Statistics and Average Odds i](#_Toc216381918)

[*Table A1: Operationalization and measurement of outcome and independent moderator variables* i](#_Toc216381919)

[*Table A2: Descriptive Statistics* i](#_Toc216381920)

[*Table A3: Average odds of discrimination favoring French over Bulgarian citizens.* i](#_Toc216381921)

[*Table A4: Average odds of discrimination favoring profiles with fluent language.* ii](#_Toc216381922)

[*Table A5: Average odds of discrimination favoring profiles with 5 work applications.* ii](#_Toc216381923)

[*Figure A1: Effect of each of the three individual variables on the odds of prioritizing French over Bulgarian profiles, by country and overall* ii](#_Toc216381924)

[Appendix 2: Preregistration iii](#_Toc216381925)

[Appendix 3: Results reported in Average Marginal Component Effects vi](#_Toc216381926)

[*Table A6: AMCE. No country differences.* vi](#_Toc216381927)

[*Table A7: AMCE. No country differences. By ideology.* vi](#_Toc216381928)

[*Table A8: AMCE. Country differences.* vi](#_Toc216381929)

[Appendix 4: Results reported in Average Marginal Component Effects using an aggregated-level simple approach viii](#_Toc216381930)

[*Table A9: Estimated Average Marginal Component Effects. No country differences.* viii](#_Toc216381931)

[*Table A10: Estimated Average Marginal Component Effects: No country differences. Nationality preferences by ideology.* viii](#_Toc216381932)

[*Table A11: Estimated Average Marginal Component Effects: Country differences.* ix](#_Toc216381933)

[Appendix 5: Assessment of differential French preference by integration efforts x](#_Toc216381934)

[*Table A12: Simple linear regression models on individual discriminations preferring French over Bulgarian profiles based on integration efforts (language and/or applications)* x](#_Toc216381935)

# Appendix 1: Operationalization, Descriptive Statistics and Average Odds

## *Table A1: Operationalization and measurement of outcome and independent moderator variables*

| **Variable** | **Survey question** | **Answer categories** |
| --- | --- | --- |
| **Outcome** | For which individual will you write a detailed personalized answer? | Individual 1  Individual 2 |
| **Ideology** | In political matters, people talk often about "the left" and "the right". On a scale from 0 (left) to 10 (right), how would you place your views on this scale? | Scale from 0-10. The “Ideology (binary)” variables taking the value 1 for cases where Ideology is strictly greater than 7, 0 otherwise. |
| **Attitudes toward migration** | To what extent do you think Switzerland [Spain, Ireland, Denmark] should allow people of a different race or ethnic group than most Swiss [Spanish, Irish, Danish] people to come and live here? | 1. Allow many  2. Allow some  3. Allow a few  4. Allow none  5. Don’t know. Removed from the comparison. |

## *Table A2: Descriptive Statistics*

|  | **Ireland N=600** | **Denmark N=655** | **Spain N=648** | **Switzerland N=500** | **p.overall** |
| --- | --- | --- | --- | --- | --- |
| **Gender:** |  |  |  |  | 0.026 |
| **Female** | 395 (65.8%) | 400 (61.1%) | 378 (58.3%) | 292 (58.4%) |  |
| **Male** | 205 (34.2%) | 255 (38.9%) | 270 (41.7%) | 208 (41.6%) |  |
| **Age** | 41.5 (12.0) | 44.6 (12.7) | 45.0 (11.5) | . (.) | <0.001 |
| **Education:** |  |  |  |  | . |
| **Other** | 0 (0.00%) | 0 (0.00%) | 3 (0.50%) | 0 (.%) |  |
| **No Education** | 6 (1.00%) | 17 (2.60%) | 1 (0.17%) | 1 (0.20%) |  |
| **Primary** | 6 (1.00%) | 20 (3.05%) | 4 (0.67%) | 5 (1.00%) |  |
| **Secondary** | 270 (45.0%) | 359 (54.8%) | 253 (42.5%) | 335 (67.0%) |  |
| **Tertiary** | 318 (53.0%) | 259 (39.5%) | 387 (59.7%) | 159 (31.8%) |  |
| **Ideology (continuous)** | -0.22 (1.93) | 0.07 (2.26) | -0.42 (2.55) | -0.04 (2.14) | 0.001 |
| **Right-wing ideology (binary)** | 0.07 (0.26) | 0.15 (0.36) | 0.13 (0.34) | 0.11 (0.31) | <0.001 |
| **Immigration openness** | 2.80 (0.81) | 2.76 (0.78) | 2.95 (0.79) | 2.64 (0.74) | <0.001 |

## *Table A3: Average odds of discrimination favoring French over Bulgarian citizens.*

| Mean | Low | High |
| --- | --- | --- |
| 1.1 | 1.03 | 1.16 |

*95% CI. N(Ireland)=600, N(Denmark)=655, N(Spain)=648, N(Switzerland)=500.*

## *Table A4: Average odds of discrimination favoring profiles with fluent language.*

| Mean | Low | High |
| --- | --- | --- |
| 1.40 | 1.31 | 1.51 |

*95% CI. N(Ireland)=600, N(Denmark)=655, N(Spain)=648, N(Switzerland)=500.*

## *Table A5: Average odds of discrimination favoring profiles with 5 work applications.*

| Mean | Low | High |
| --- | --- | --- |
| 2.13 | 2.00 | 2.28 |

*95% CI. N(Ireland)=600, N(Denmark)=655, N(Spain)=648, N(Switzerland)=500.*

## *Figure A1: Effect of each of the three individual variables on the odds of prioritizing French over Bulgarian profiles, by country and overall*


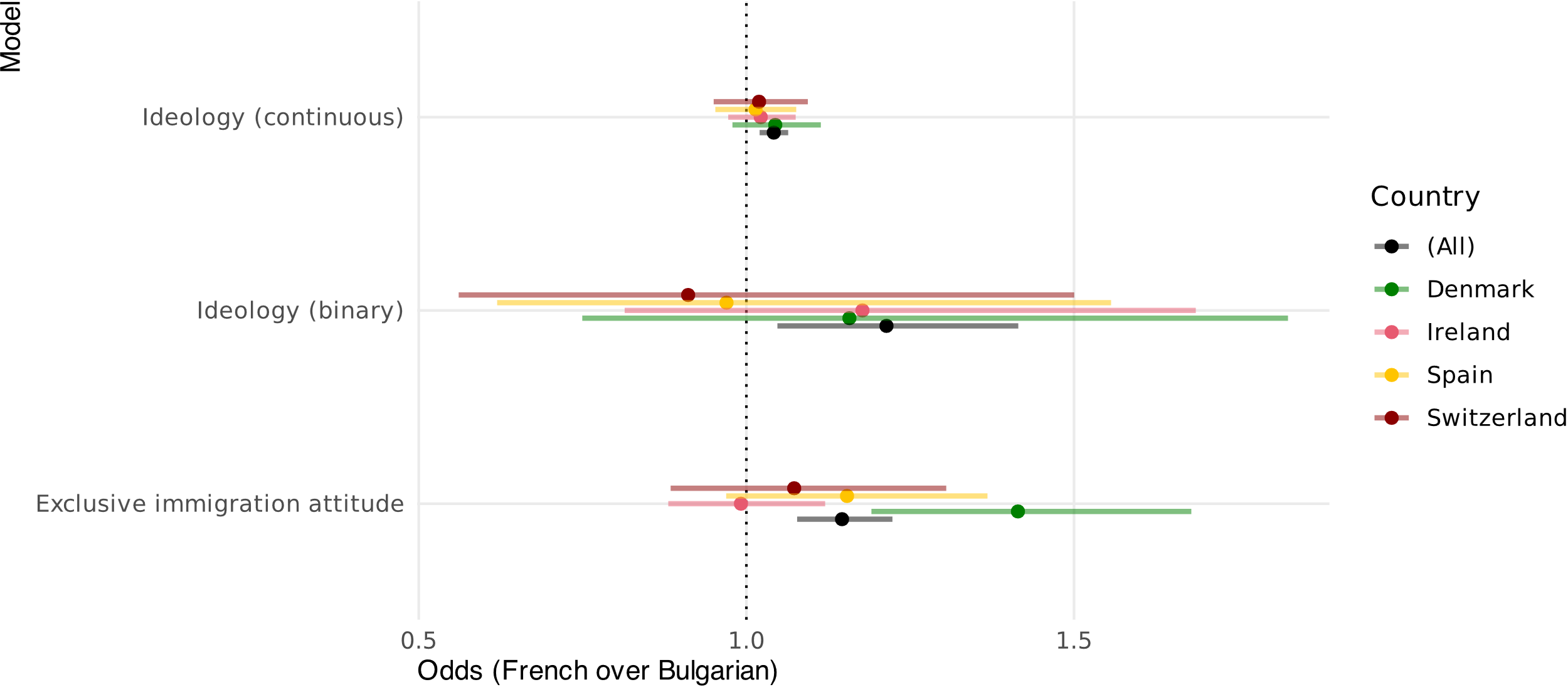


*Note: The dot represents the median of the posterior density, and the horizontal line covers the 95% credible interval.*

# Appendix 2: Preregistration

The preregistration of this research project details all hypotheses, the study design, and analysis plan prior to data collection. We commit to our preregistration in terms of the empirical sample (bureaucrats) and analysis plan. Note, that the hypotheses listed in this preregistration will be tested in other studies. The hypotheses on nationality (H1) and language skills (H2) that we test in this particular paper have been developed based on earlier preregistered work (and hypotheses) by the authors (see Adam et al., 2021). Our hypotheses on heterogeneous treatment effects (H1a, H1b) and on job-seeking efforts (H3) have not been preregistered, but they are developed from the relevant literature. However, point 8 in our preregistration for this project already declares our interest in exploring heterogeneous treatment effects of the kind of H1a, H1b. Taken together, our hypotheses allow us to systematically test the relative importance of identity versus effort-based signals in shaping bureaucrats’ real-world behavior.

The full preregistration PDF is included below in this appendix for reference.

*
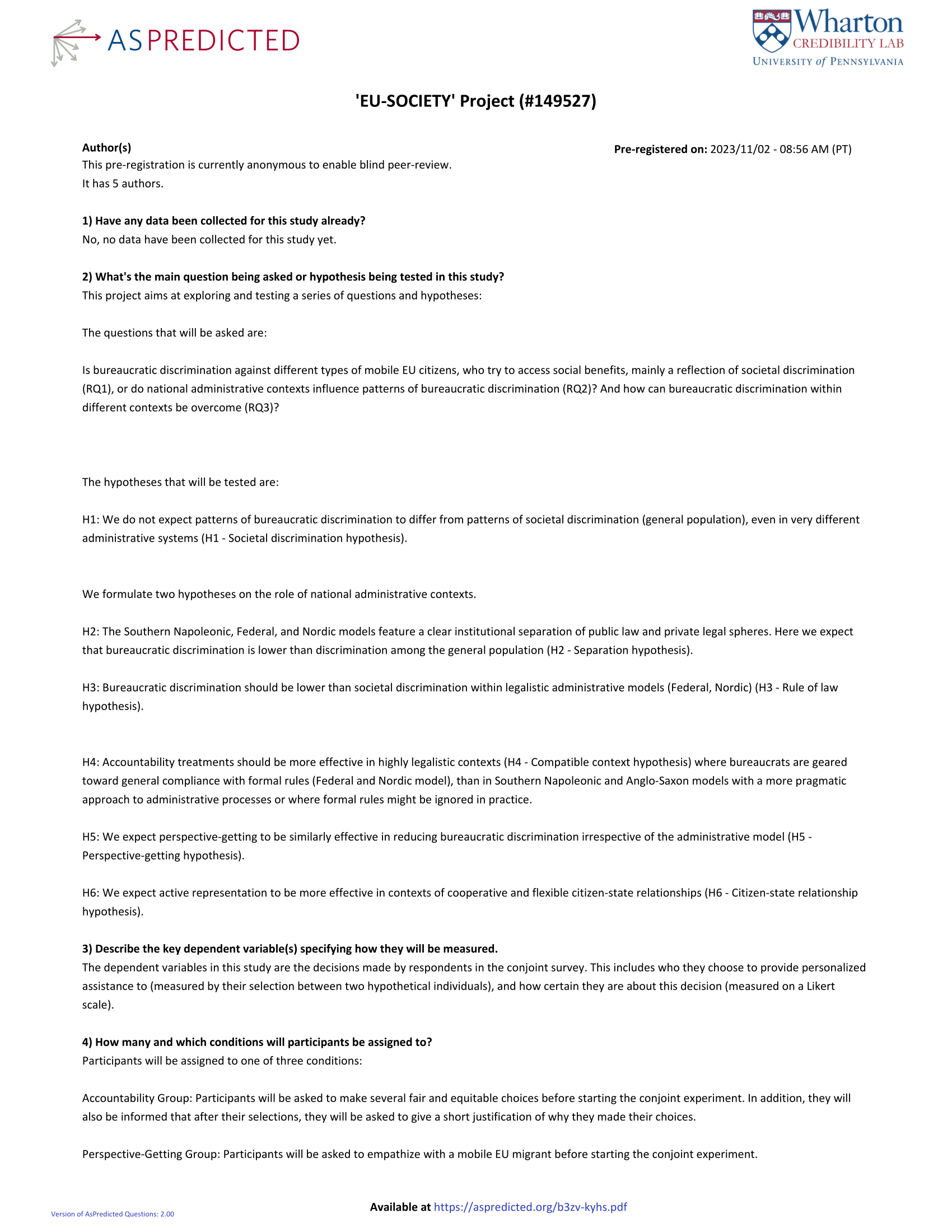
*

*
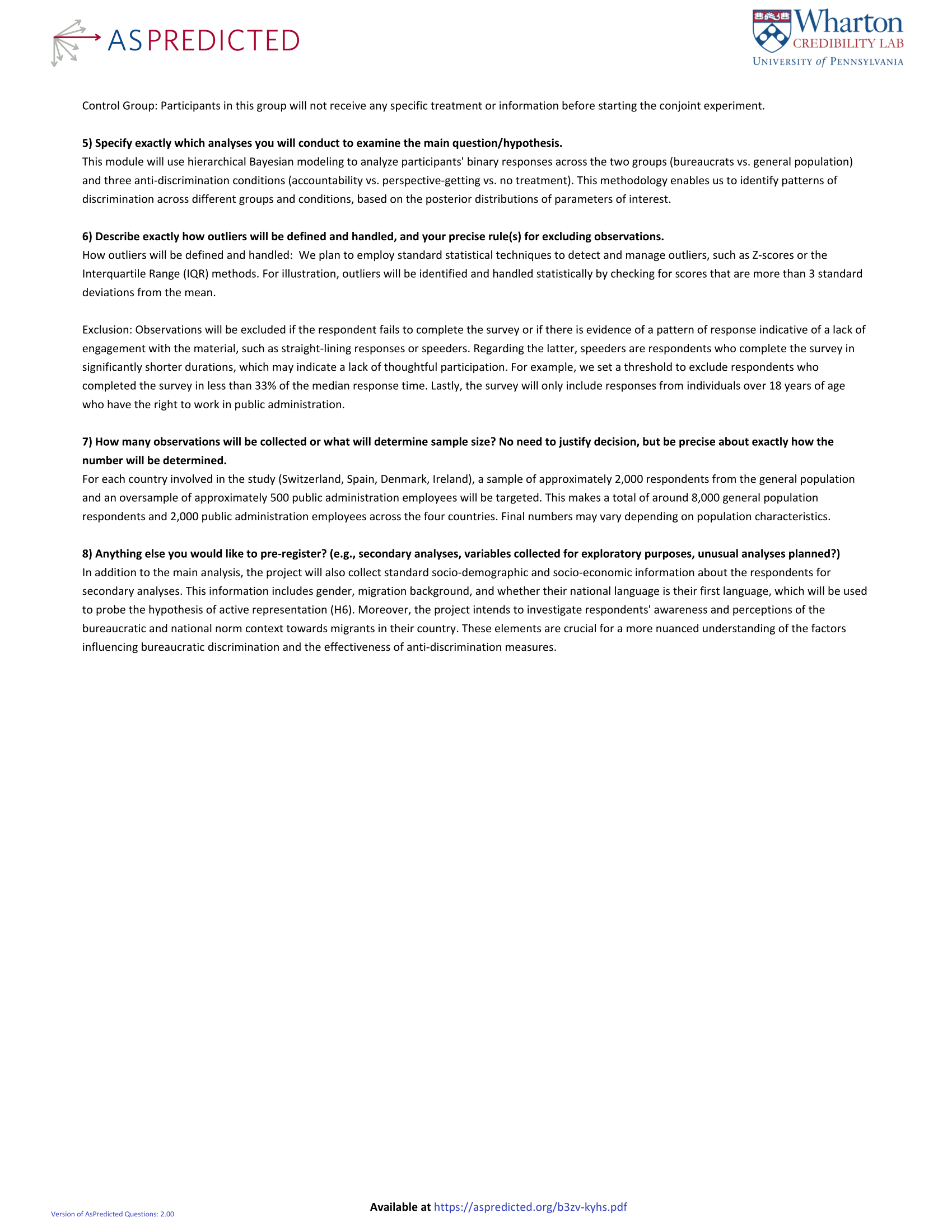
*

# Appendix 3: Results reported in Average Marginal Component Effects

## *Table A6: AMCE. No country differences.*

| **Feature** | **AMCE** |
| --- | --- |
| Nationality: French | 0.0126 |
| Gender: Male | -0.0393 |
| Profession: Sales Person | -0.0562 |
| Language: Limited | -0.0530 |
| Age: 55 | 0.0053 |
| Applications: 5 | 0.1228 |
| Stay: 4 Years | 0.0644 |
| Shown: Second | -0.0325 |

## *Table A7: AMCE. No country differences. By ideology.*

| **Feature** | **Ideology** | **AMCE** |
| --- | --- | --- |
| Nationality: French | No right-wing | 0.0088 |
| Nationality: French | Right-wing | 0.0406 |
| Gender: Male | No right-wing | -0.0437 |
| Gender: Male | Right-wing | -0.0072 |
| Profession: Sales Person | No right-wing | -0.0521 |
| Profession: Sales Person | Right-wing | -0.0878 |
| Language: Limited | No right-wing | -0.0451 |
| Language: Limited | Right-wing | -0.1121 |
| Age: 55 | No right-wing | 0.0071 |
| Age: 55 | Right-wing | -0.0087 |
| Applications: 5 | No right-wing | 0.1263 |
| Applications: 5 | Right-wing | 0.0982 |
| Stay: 4 Years | No right-wing | 0.0635 |
| Stay: 4 Years | Right-wing | 0.0711 |
| Shown: Second | No right-wing | -0.0326 |
| Shown: Second | Right-wing | -0.0319 |

## *Table A8: AMCE. Country differences.*

| **Feature** | **Country** | **AMCE** |
| --- | --- | --- |
| Nationality: French | Denmark | 0.0219 |
| Nationality: French | Ireland | 0.0014 |
| Nationality: French | Spain | 0.0204 |
| Nationality: French | Switzerland | 0.0066 |
| Gender: Male | Denmark | -0.0366 |
| Gender: Male | Ireland | -0.0382 |
| Gender: Male | Spain | -0.0486 |
| Gender: Male | Switzerland | -0.0335 |
| Language: Limited | Denmark | -0.1066 |
| Language: Limited | Ireland | -0.0131 |
| Language: Limited | Spain | -0.0190 |
| Language: Limited | Switzerland | -0.0747 |
| Profession: Sales Person | Denmark | -0.1037 |
| Profession: Sales Person | Ireland | -0.0867 |
| Profession: Sales Person | Spain | -0.0290 |
| Profession: Sales Person | Switzerland | 0.0070 |
| Age: 55 | Denmark | -0.0255 |
| Age: 55 | Ireland | -0.0029 |
| Age: 55 | Spain | 0.0393 |
| Age: 55 | Switzerland | 0.0111 |
| Applications: 5 | Denmark | 0.1319 |
| Applications: 5 | Ireland | 0.1371 |
| Applications: 5 | Spain | 0.0862 |
| Applications: 5 | Switzerland | 0.1420 |
| Stay: 4 Years | Denmark | 0.0563 |
| Stay: 4 Years | Ireland | 0.0670 |
| Stay: 4 Years | Spain | 0.0719 |
| Stay: 4 Years | Switzerland | 0.0624 |
| Shown: Second | Denmark | -0.0219 |
| Shown: Second | Ireland | -0.0346 |
| Shown: Second | Spain | -0.0403 |
| Shown: Second | Switzerland | -0.0349 |

# Appendix 4: Results reported in Average Marginal Component Effects using an aggregated-level simple approach

## *Table A9: Estimated Average Marginal Component Effects. No country differences.*

| **Feature** | **Level** | **AMCE Estimate** | **SE** | **p-value** | **CI** |
| --- | --- | --- | --- | --- | --- |
| Nationality | French | 0.0126 | 0.0050 | 0.0108 | [0.0029 : 0.022] |
| Gender | Male | -0.0393 | 0.0047 | 0.0000 | [-0.048 : -0.03] |
| Language | Limited | -0.0530 | 0.0066 | 0.0000 | [-0.066 : -0.04] |
| Profession | Sales Person | -0.0562 | 0.0059 | 0.0000 | [-0.068 : -0.045] |
| Age | 55 | 0.0053 | 0.0051 | 0.2995 | [-0.0047 : 0.015] |
| Applications | 5 | 0.1228 | 0.0057 | 0.0000 | [0.11 : 0.13] |
| Stay | 4 Years | 0.0644 | 0.0054 | 0.0000 | [0.054 : 0.075] |
| Shown | Second | -0.0325 | 0.0069 | 0.0000 | [-0.046 : -0.019] |

## *Table A10: Estimated Average Marginal Component Effects: No country differences. Nationality preferences by ideology.*

| **Ideology** | **Feature** | **Level** | **AMCE Estimate** | **SE** | **p-value** | **CI** |
| --- | --- | --- | --- | --- | --- | --- |
| No right-wing | Nationality | French | 0.0088 | 0.0053 | 0.0955 | [-0.0015 : 0.019] |
| Right-wing | Nationality | French | 0.0406 | 0.0147 | 0.0058 | [0.012 : 0.069] |
| No right-wing | Gender | Male | -0.0437 | 0.0049 | 0.0000 | [-0.053 : -0.034] |
| Right-wing | Gender | Male | -0.0072 | 0.0148 | 0.6259 | [-0.036 : 0.022] |
| No right-wing | Language | Limited | -0.0451 | 0.0070 | 0.0000 | [-0.059 : -0.031] |
| Right-wing | Language | Limited | -0.1121 | 0.0193 | 0.0000 | [-0.15 : -0.074] |
| No right-wing | Profession | Sales Person | -0.0521 | 0.0063 | 0.0000 | [-0.064 : -0.04] |
| Right-wing | Profession | Sales Person | -0.0878 | 0.0178 | 0.0000 | [-0.12 : -0.053] |
| No right-wing | Age | 55 | 0.0071 | 0.0054 | 0.1877 | [-0.0034 : 0.018] |
| Right-wing | Age | 55 | -0.0087 | 0.0159 | 0.5831 | [-0.04 : 0.022] |
| No right-wing | Applications | 5 | 0.1263 | 0.0061 | 0.0000 | [0.11 : 0.14] |
| Right-wing | Applications | 5 | 0.0982 | 0.0158 | 0.0000 | [0.067 : 0.13] |
| No right-wing | Stay | 4 Years | 0.0635 | 0.0057 | 0.0000 | [0.052 : 0.075] |
| Right-wing | Stay | 4 Years | 0.0711 | 0.0162 | 0.0000 | [0.039 : 0.1] |
| No right-wing | Shown | Second | -0.0326 | 0.0073 | 0.0000 | [-0.047 : -0.018] |
| Right-wing | Shown | Second | -0.0319 | 0.0205 | 0.1197 | [-0.072 : 0.0083] |

## *Table A11: Estimated Average Marginal Component Effects: Country differences.*

| **Country** | **Feature** | **Level** | **AMCE Estimate** | **SE** | **p-value** | **CI** |
| --- | --- | --- | --- | --- | --- | --- |
| Denmark | Nationality | French | 0.0219 | 0.0094 | 0.0193 | [0.0036 : 0.04] |
| Ireland | Nationality | French | 0.0014 | 0.0099 | 0.8906 | [-0.018 : 0.021] |
| Spain | Nationality | French | 0.0204 | 0.0095 | 0.0320 | [0.0018 : 0.039] |
| Switzerland | Nationality | French | 0.0066 | 0.0109 | 0.5429 | [-0.015 : 0.028] |
| Denmark | Gender | Male | -0.0366 | 0.0091 | 0.0001 | [-0.054 : -0.019] |
| Ireland | Gender | Male | -0.0382 | 0.0089 | 0.0000 | [-0.056 : -0.021] |
| Spain | Gender | Male | -0.0486 | 0.0089 | 0.0000 | [-0.066 : -0.031] |
| Switzerland | Gender | Male | -0.0335 | 0.0107 | 0.0017 | [-0.054 : -0.013] |
| Denmark | Language | Limited | -0.1066 | 0.0134 | 0.0000 | [-0.13 : -0.08] |
| Ireland | Language | Limited | -0.0131 | 0.0127 | 0.3026 | [-0.038 : 0.012] |
| Spain | Language | Limited | -0.0190 | 0.0130 | 0.1439 | [-0.044 : 0.0065] |
| Switzerland | Language | Limited | -0.0747 | 0.0126 | 0.0000 | [-0.1 : -0.05] |
| Denmark | Profession | Sales Person | -0.1037 | 0.0107 | 0.0000 | [-0.12 : -0.083] |
| Ireland | Profession | Sales Person | -0.0867 | 0.0119 | 0.0000 | [-0.11 : -0.063] |
| Spain | Profession | Sales Person | -0.0290 | 0.0109 | 0.0081 | [-0.05 : -0.0075] |
| Switzerland | Profession | Sales Person | 0.0070 | 0.0136 | 0.6053 | [-0.02 : 0.034] |
| Denmark | Age | 55 | -0.0255 | 0.0092 | 0.0057 | [-0.044 : -0.0074] |
| Ireland | Age | 55 | -0.0029 | 0.0102 | 0.7723 | [-0.023 : 0.017] |
| Spain | Age | 55 | 0.0393 | 0.0100 | 0.0001 | [0.02 : 0.059] |
| Switzerland | Age | 55 | 0.0111 | 0.0114 | 0.3284 | [-0.011 : 0.033] |
| Denmark | Applications | 5 | 0.1319 | 0.0111 | 0.0000 | [0.11 : 0.15] |
| Ireland | Applications | 5 | 0.1371 | 0.0112 | 0.0000 | [0.12 : 0.16] |
| Spain | Applications | 5 | 0.0862 | 0.0111 | 0.0000 | [0.064 : 0.11] |
| Switzerland | Applications | 5 | 0.1420 | 0.0123 | 0.0000 | [0.12 : 0.17] |
| Denmark | Stay | 4 Years | 0.0563 | 0.0099 | 0.0000 | [0.037 : 0.076] |
| Ireland | Stay | 4 Years | 0.0670 | 0.0113 | 0.0000 | [0.045 : 0.089] |
| Spain | Stay | 4 Years | 0.0719 | 0.0102 | 0.0000 | [0.052 : 0.092] |
| Switzerland | Stay | 4 Years | 0.0624 | 0.0118 | 0.0000 | [0.039 : 0.086] |
| Denmark | Shown | Second | -0.0219 | 0.0127 | 0.0862 | [-0.047 : 0.0031] |
| Ireland | Shown | Second | -0.0346 | 0.0143 | 0.0154 | [-0.063 : -0.0066] |
| Spain | Shown | Second | -0.0403 | 0.0135 | 0.0028 | [-0.067 : -0.014] |
| Switzerland | Shown | Second | -0.0349 | 0.0149 | 0.0194 | [-0.064 : -0.0056] |

# Appendix 5: Assessment of differential French preference by integration efforts

## *Table A12: Simple linear regression models on individual discriminations preferring French over Bulgarian profiles based on integration efforts (language and/or applications). 95 percent credible intervals between square brackets.*

|  | **Language** | **Applications** | **Language + Applications** |
| --- | --- | --- | --- |
| **(Intercept)** | 0.147 | 0.212 | 0.275 |
|  | [0.091, 0.205] | [0.150, 0.275] | [0.213, 0.336] |
| **Language Fluent** | -0.125 |  | -0.141 |
|  | [-0.155, -0.093] |  | [-0.173, -0.109] |
| **Applications 5** |  | -0.142 | -0.161 |
|  |  | [-0.178, -0.106] | [-0.195, -0.127] |
| **Num.Obs.** | 2403 | 2403 | 2403 |
| **R2** | 0.025 | 0.026 | 0.058 |
| **Log.Lik.** | -4224.196 | -4221.905 | -4182.916 |
