# Supplementary material for: Identity- versus effort-based bureaucratic discrimination among mobile European Union citizens: Evidence from conjoint experiments
Source: Eur Union Polit. 2026 Mar 4;27(2):301–24. doi: 10.1177/14651165261423087 (PMC13218594; doi:10.1177/14651165261423087)
Supplement: sj-zip-3-eup-10.1177_14651165261423087 - Supplemental material for Identity- versus effort-based bureaucratic discrimination among mobile European Union citizens: Evidence from conjoint experiments [file sj-zip-3-eup-10.1177_14651165261423087.zip › weave-p7.pdf]

JANA GÓMEZ-DÍAZ, EVA THOMANN, ANITA MANATSCHAL,

XAVIER FERNÁNDEZ-I-MARÍN

IDENTITY VERSUS EFFORT-BASED BUREAUCRATIC DISCRIMINATION AMONG MOBILE EUROPEAN UNION CITIZENS: A CONJOINT EXPERIMENT

Contents

1 *Paper 7:* 5

2 *Estimation of individual utilities: Hierarchical Bayes through JAGS, N(0, 2.5)* 7

3 *Paper 7: on the bureaucrats subsample* 19

Programming environment 53

1
Paper 7:

```r
source("load_packages.R")
source("functions.R")
load("data-weave.RData")

library(brms)
library(marginaleffects)
```

2

Estimation of individual utilities: Hierarchical Bayes through JAGS, N(0, 2.5)

Use the whole dataset, and compare the effectiveness of treatment to decrease discriminatory patterns.

```
d ← E %>%
  select(id, Decision,
         Nationality, Gender, Language, Profession, Age, Applications, Stay,
         Shown,
         Population,
         Treatment,
         Country) %>%
  mutate(idi = as.integer(as.factor(id)))


nId ← length(unique(d$id))
Y ← d$Decision
nO ← length(Y)

X ← d %>%
  mutate(`Nationality: French` = ifelse(Nationality == "French", 1, 0)) %>%
  mutate(`Gender: Male` = ifelse(Gender == "Male", 1, 0)) %>%
  mutate(`Gender: Female` = ifelse(Gender == "Male", 0, 1)) %>%
  mutate(`Profession: Medical Doctor` = ifelse(Profession == "Medical Doctor", 1, 0)) %>%
  mutate(`Language: Fluent` = ifelse(Language == "Fluent", 1, 0)) %>%
  mutate(`Age: 25` = ifelse(Age == "25", 1, 0)) %>%
  mutate(`Age: 55` = ifelse(Age == "55", 1, 0)) %>%
  mutate(`Applications: 5` = ifelse(Applications == "5", 1, 0)) %>%
  mutate(`Stay: 4 Years` = ifelse(Stay == "4 Years", 1, 0)) %>%
  mutate(`First shown` = ifelse(Shown == "First", 1, 0)) %>%
  mutate(`Shown: First` = ifelse(Shown == "First", 1, 0)) %>%
  select(`Nationality: French`,
         `Gender: Female`,
         `Profession: Medical Doctor`,
         `Language: Fluent`,
         `Age: 55`,
         `Applications: 5`,
         `Stay: 4 Years`,
```

```
         `Shown: First`) %>%
  as.matrix()
nF ← dim(X)[[2]]
feature.label ← dimnames(X)[[2]]

b0 ← rep(0, nF)
B0 ← B0O ← diag(nF)
diag(B0) ← 2.5^-2
diag(B0O) ← 1^-2
diag(B0O)[length(diag(B0O))] ← 0.5^-2 # first shown

D ← list(
  nO = nO,
  X = unname(X), nF = nF, b0 = b0, B0 = B0, B0O = B0O,
  id = d$idi,
  nId = nId,
  Y = Y)
```

This model estimates approximately the following number of parameters:

```
# beta
(nId * nF) +
# sigma_beta
(nF) +
# theta
#(nP * nF * nCov) +
## Theta
#(nOutcome * nF) +
# Omega
(nF) #+

→ [1] 82400

# sigma_theta
#(nOutcome * nF) +
# sigma_Theta
#(nF) +
# v-cov
#(nF * nF)

M ← "HB-JAGS with N(0, 2.5)"
M.lab ← "hb-jags-normal-2dot5"
m ← "
model {
  for (o in 1:nO) {
    Y[o] ~ dbern(p[o])
    logit(p[o]) ← alpha + inprod(beta[id[o],1:nF], X[o,1:nF])
  }
  # Priors for effects
  alpha ~ dnorm(0, 2.5^-2)
```

```
  for (f in 1:nF) {
    for (id in 1:nId) {
      #beta[id,f] ~ dnorm(mu[id,f], 2.5^-2)
      beta[id,f] ~ dnorm(0, 2.5^-2)
      #beta[id,f] ~ dnorm(0, 1^-2)T(-3,3)
      #beta[id,f] ~ dnorm(0, 1^-2)
      #beta[id,f] ~ dnorm(Omega[f], 1^-2)
      #beta[id,f] ~ dnorm(Omega[f], tau_beta[f])
      #beta[id,f] ~ dt(0, 1^-2, 3)
      #mu[id,f] <- Omega[f] + inprod(theta[f,id_treatment[id],1:nCov], C[id,1:nCov])
      #mu[id,f] <- Omega[f] + inprod(theta[f,1:nCov], C[id,1:nCov])
      mu[id,f] <- Omega[f]
    }
    tau_beta[f] ~ dgamma(1, 1)
    sigma_beta[f] <- 1 / sqrt(tau_beta[f])
    #for (cov in 1:nCov) {
    #  tau_theta[f,cov] <- pow(sigma_theta[f,cov], -2)
    #  sigma_theta[f,cov] ~ dnorm(0, 2.0^-2)T(0,)
    #  theta[f,cov] ~ dnorm(0, 2.5^-2)
    #}
  }
  tau_Theta <- pow(sigma_Theta,- 2)
  sigma_Theta ~ dt(0, 1^-2, 3)T(0,)
  Omega ~ dmnorm(b0, Tau_Omega)
  Tau_Omega ~ dwish(B0O, nF + 1)
  Sigma_Omega <- inverse(Tau_Omega)
}
"
write(m, file = paste("models/model-", M.lab, ".bug", sep = ""))
par <- NULL
par <- c(par, "theta")
par <- c(par, "Theta", "sigma_theta")
par <- c(par, "sigma_Theta")
par <- c(par, "Sigma_Omega")
par <- c(par, "Omega")
par <- c(par, "sigma_beta")
par <- c(par, "alpha")
par.beta <- c("beta")
par.pcp <- c("p")
adapt <- 1e3
burnin <- 5e3#2e3#2e4
run <- 2e3
run.beta <- 2e3#400
run.p <- 50
chains <- 3
method <- "parallel"
thin <- 10
adapt <- 500; burnin <- 1000; run <- 1e3; run.beta <- 400; run.p <- 50; thin <- 5
adapt <- 200; burnin <- 1e3; run <- 1e3; run.beta <- 800; run.p <- 50; thin <- 1 # 16'
```

```
inits.beta <- array(0, dim = c(nId, nF))


inits <- list(
  list(
    .RNG.name = "base::Super-Duper", .RNG.seed = 1,
    beta = inits.beta),
  list(
    .RNG.name = "base::Super-Duper", .RNG.seed = 2,
    beta = inits.beta),
  list(
    .RNG.name = "base::Wichmann-Hill", .RNG.seed = 3,
    beta = inits.beta))

load(file = paste("ci_beta-", M.lab, ".RData", sep = ""))
```

2.1 *Part-worth contributions*

```
ci.beta %>%
  mutate(Side = ifelse(median < 0, "Negative", "Positive")) %>%
  ggplot(aes(x = median, color = Side, fill = Side)) +
  geom_histogram(data = . %>% filter(Side == "Positive"), binwidth = 0.02) +
  geom_histogram(data = . %>% filter(Side == "Negative"), binwidth = 0.02) +
  facet_grid(Feature ~ .) +
  xlab("Utility") + ylab("n") +
  theme(strip.text.y = element_text(angle = 0, hjust = 0))


ci.beta %>%
  left_join(select(I, id, Population)) %>%
  filter(Population == "Public administration") %>%
  filter(Treatment %in% c("Control", "Perspective-getting")) %>%
  mutate(Side = ifelse(median < 0, "Negative", "Positive")) %>%
  ggplot(aes(x = median, color = Side, fill = Side)) +
  geom_histogram(data = . %>% filter(Side == "Positive"), binwidth = 0.02) +
  geom_histogram(data = . %>% filter(Side == "Negative"), binwidth = 0.02) +
  facet_grid(Feature ~ .) +
  xlab("Utility") + ylab("n") +
  theme(strip.text.y = element_text(angle = 0, hjust = 0))


ci.beta.w <- ci.beta %>%
  select(-c(Model, Country)) %>%
  pivot_wider(names_from = Feature, values_from = median)# %>%
#  left_join(select(I, id, Treatment))

my_dens <- function(data, mapping, ..., low = "#132B43", high = "#56B1F7") {
  ggplot(data = data, mapping=mapping) +
         geom_density(..., alpha=0.3)
}
```

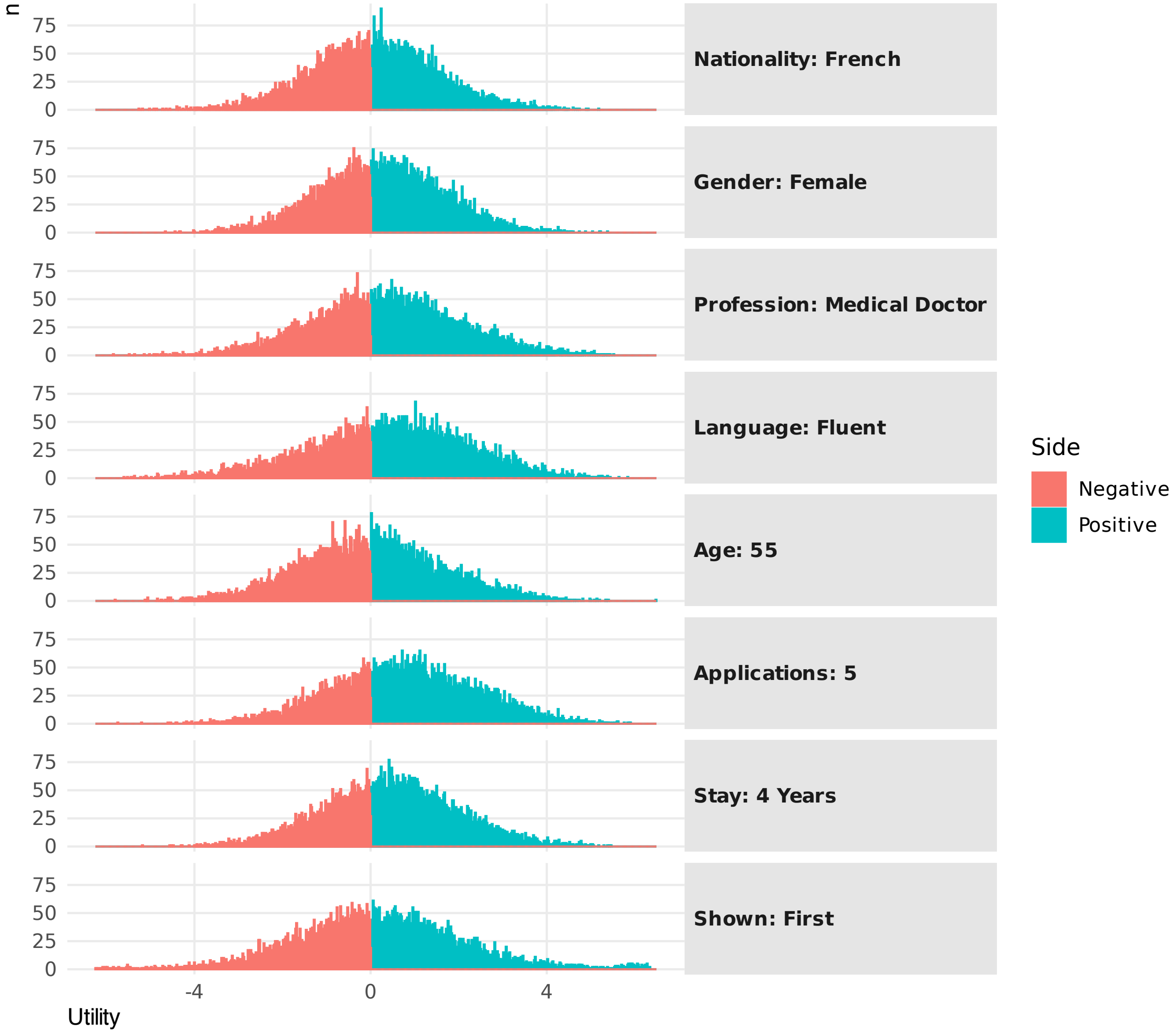

Figure 2.1: Distribution of the mean values for each individuals' utilities. Colors represent positive/negative utilities.

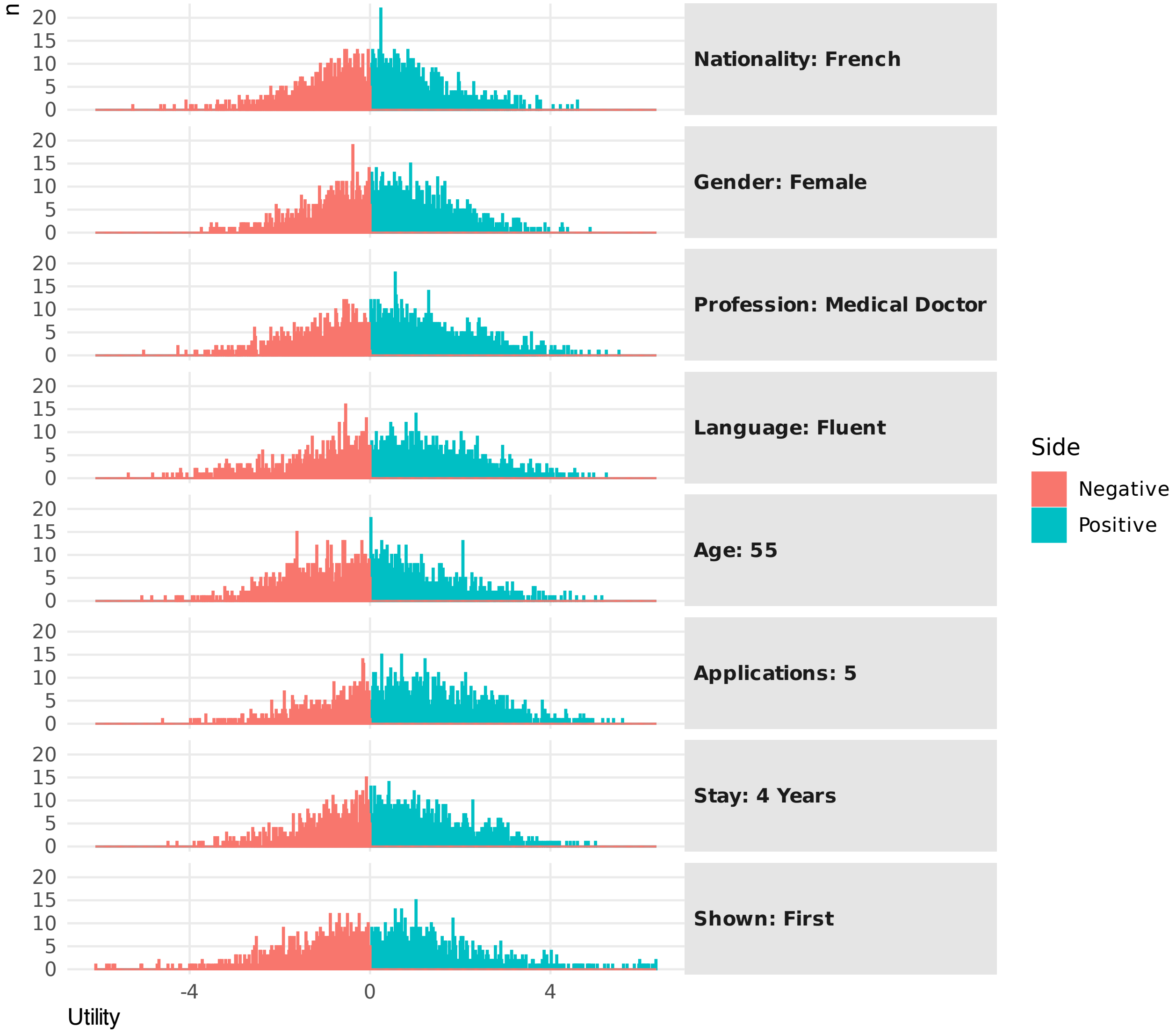

Figure 2.2: Distribution of the mean values for each individuals' utilities. Colors represent positive/negative utilities.

```
my_points ← function(data, mapping, ... , low = "#132B43", high = "#56B1F7") {
  ggplot(data = data, mapping=mapping) +
         geom_point( ... , alpha=0.1) +
         geom_smooth(method = "lm")
}

ggpairs(select(ci.beta.w, -id),
  #lower = list(continuous = wrap("points", alpha = 0.1)),
  lower = list(continuous = my_points),
  diag = list(continuous = my_dens),
  mapping = aes(color = Treatment))
```

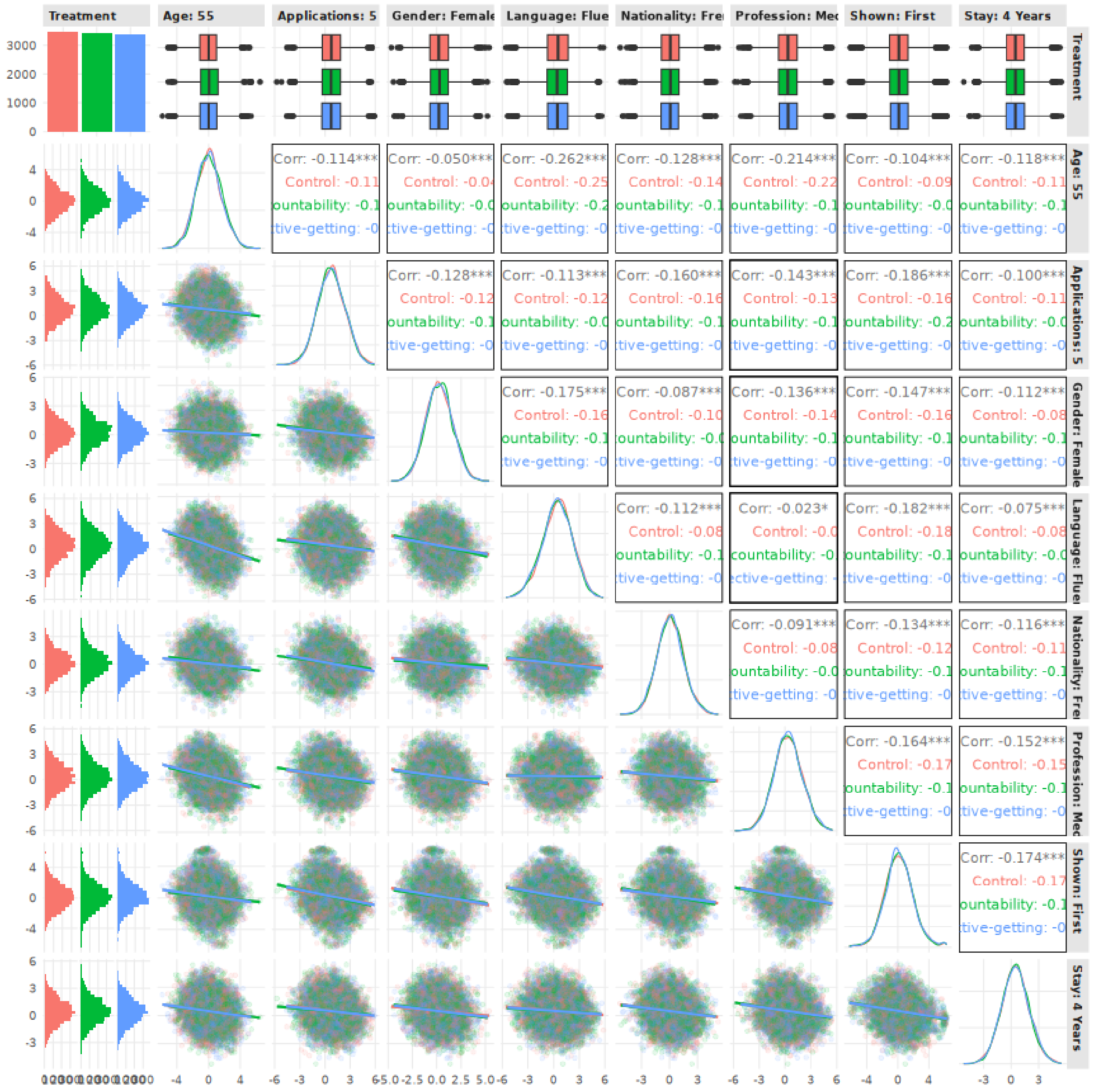

Figure 2.3: Comparison of individual utilities of every feature.

```
ci.beta.w ← ci.beta %>%
```

```
  select(-c(Model, Treatment)) %>%
  pivot_wider(names_from = Feature, values_from = median)# %>%
#  left_join(select(I, id, Treatment))

my_dens ← function(data, mapping,  ... , low = "#132B43", high = "#56B1F7") {
  ggplot(data = data, mapping=mapping) +
         geom_density( ... , alpha=0.3)
}
my_points ← function(data, mapping,  ... , low = "#132B43", high = "#56B1F7") {
  ggplot(data = data, mapping=mapping) +
         geom_point( ... , alpha=0.1) +
         geom_smooth(method = "lm")
}

ggpairs(select(ci.beta.w, -id),
  #lower = list(continuous = wrap("points", alpha = 0.1)),
  lower = list(continuous = my_points),
  diag = list(continuous = my_dens),
  mapping = aes(color = Country))


convergence.beta$effective %>%
  ggplot(aes(x = Effective)) +
  geom_histogram() +
  expand_limits(x = 0)


convergence.beta$rhat%>%
  ggplot(aes(x = Rhat)) +
  geom_histogram(binwidth = 0.001) +
  expand_limits(x = c(1, 1.5))


p.out ← convergence.beta$geweke %>%
  summarize(pOut = length(which(Geweke < -2 | Geweke > 2)) / n()) %>%
  select(pOut) %>%
  unlist(., use.names = FALSE)

convergence.beta$geweke%>%
  ggplot(aes(x = Geweke)) +
  geom_histogram(aes(y = ..density..), bins = 80) +
  geom_vline(xintercept = c(-2, 2), lty = 3) +
  stat_function(fun = dnorm, args = list(mean = 0, sd = 1), color = "blue", size = 1) +
  annotate("text", x = -Inf, y = Inf, label = paste("p(out) = ", round(p.out, 3)), hjust = 0, vjust = 1)

source("load_packages.R")
source("functions.R")
load("data-weave.RData")

library(brms)
library(rstanarm)
```

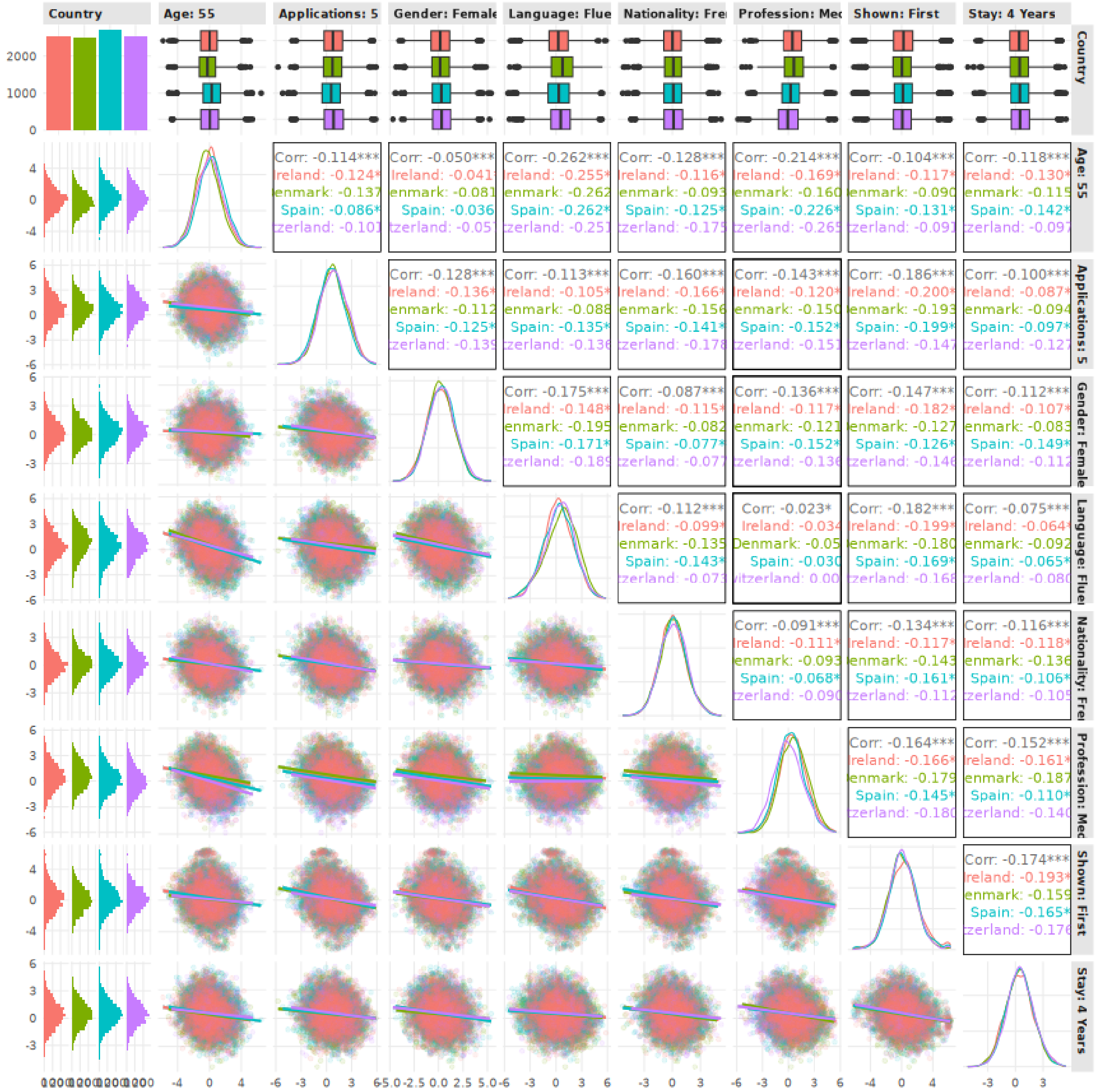

Figure 2.4: Comparison of individual utilities of every feature.

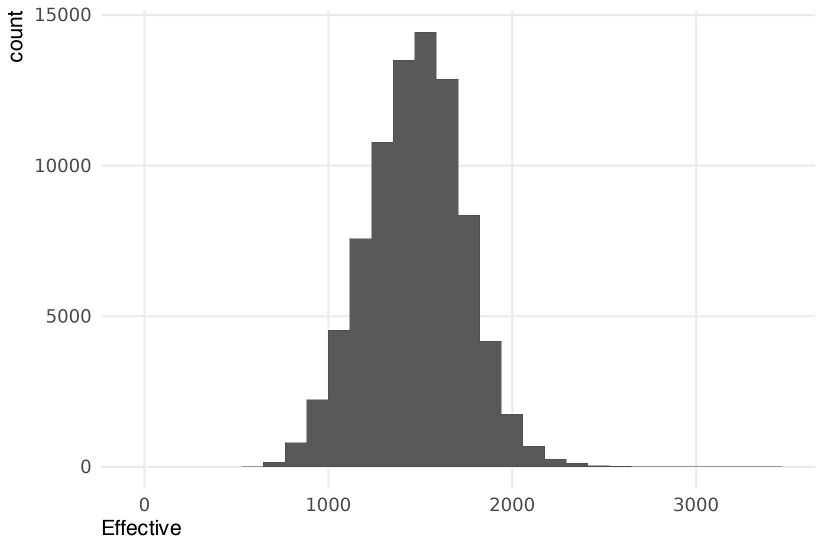

Figure 2.5: Convergence diagnostics: distribution of the number of effective samples for all parameters.

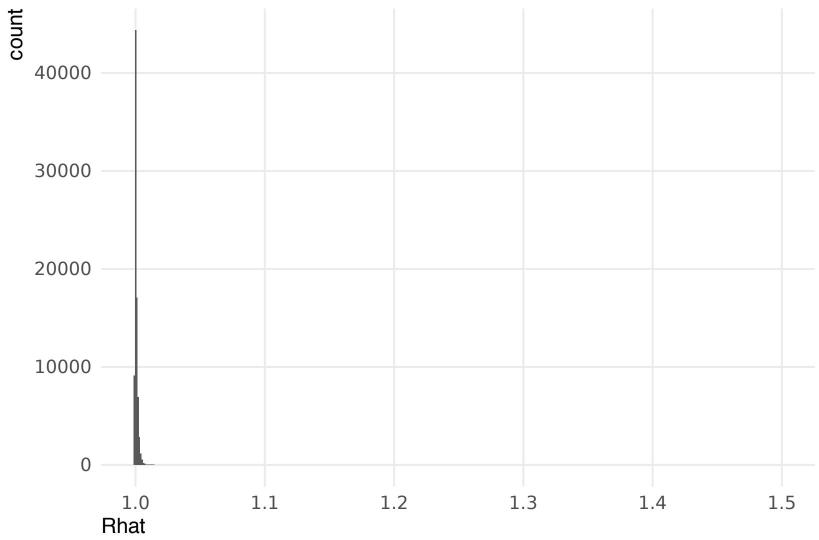

Figure 2.6: Convergence diagnostics: distribution of Rhat values for all parameters.

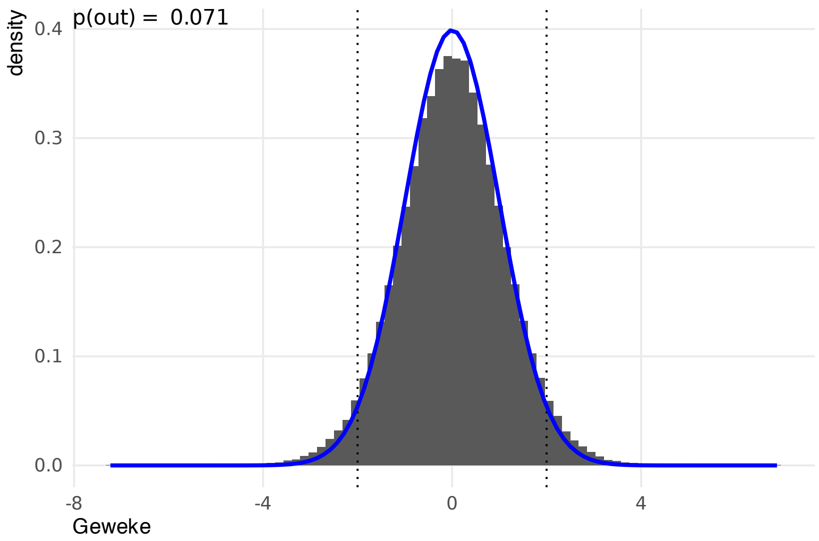

Figure 2.7: Convergence diagnostics: distribution of Geweke z-scores values for all parameters.

```
library(marginaleffects)
library(broom.mixed)
library(modelsummary)
```

3
Paper 7: on the bureaucrats subsample

Use the bureaucrats subsample. Do it using individual utilities.

```
d ← E %>%
  select(id, Decision,
         Nationality, Gender, Language, Profession, Age, Applications, Stay,
         Shown,
         Population,
         Treatment,
         Country) %>%
  mutate(idi = as.integer(as.factor(id)))

############## CHOOSE THE HB SOURCE

# Load bulk estimation
M.lab ← "hb-jags-normal-2dot5"
#M.lab ← "hb-jags-t-1-3"
load(file = paste("ci_beta-", M.lab, ".RData", sep = ""))



############## END CHOOSE THE HB SOURCE


# Restrict to bureaucrats
d ← d %>%
  filter(Population == "Public administration") %>%
  droplevels()
ci.beta ← ci.beta %>%
  filter(id %in% d$id) %>%
  droplevels() %>%
  left_join(select(I, id,
                   Country,
                   Migration,
                   `Immigration openess`,
                   Ideology, `Right-wing ideology`)) %>%
  rename(`Ideology (continuous)` = Ideology) %>%
  rename(`Ideology (binary)` = `Right-wing ideology`) %>%
  mutate(`Immigration openess` = `Immigration openess` - median(`Immigration openess`, na.rm = TRUE)) %>%
```

```
  mutate(`Exclusive immigration attitude` = -`Immigration openess` - median(-`Immigration openess`, na.rm =

d %>%
  select(id, Country, Population) %>%
  distinct() %>%
  count(Country, Population) %>%
  mykbl("Sample description.")
```

Table 3.1: Sample description.

Country	Population	n
Ireland	Public administration	600
Denmark	Public administration	655
Spain	Public administration	648
Switzerland	Public administration	500

3.1 *H1: There is discrimination favouring French over Bulgarian citizens*

```
ci.beta %>%
  filter(Feature == "Nationality: French") %>%
  mutate(Odds = exp(median)) %>%
  ggplot(aes(x = Odds)) +
  geom_histogram() +
  scale_x_continuous(trans = "log2") +
  geom_vline(xintercept = 1, lty = 3)
```

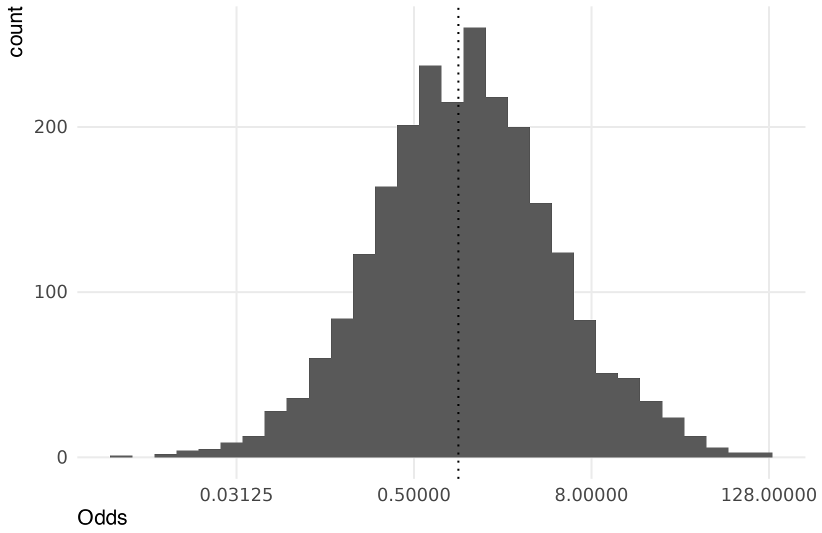

Figure 3.1: Individual utilities for prioritization of French citizens over Bulgarian (odds).

```
ci.beta %>%
  filter(Feature == "Nationality: French") %>%
  reframe(getci(median)) %>%
  pivot_longer(everything(), names_to = "Variable", values_to = "value") %>%
  mutate(value = exp(value)) %>%
  pivot_wider(names_from = Variable, values_from = value) %>%
  mykbl("Average odds of discrimination favouring French over Bulgarian citizens.")
```

Table 3.2: Average odds of discrimination favouring French over Bulgarian citizens.

Mean	low	high	Low	High
1.11	1.05	1.18	1.06	1.16

```
ci.beta %>%
  filter(Feature == "Nationality: French") %>%
  group_by(Country) %>%
  reframe(getci(median)) %>%
  ungroup() %>%
  pivot_longer(-Country, names_to = "Variable", values_to = "value") %>%
  mutate(value = exp(value)) %>%
  pivot_wider(names_from = Variable, values_from = value) %>%
  mykbl("Average odds of discrimination favouring French over Bulgarian citizens, by country.")
```

Table 3.3: Average odds of discrimination favouring French over Bulgarian citizens, by country.

Country	Mean	low	high	Low	High
Ireland	1.03	0.92	1.15	0.93	1.13
Denmark	1.09	0.97	1.22	0.99	1.20
Spain	1.17	1.05	1.31	1.07	1.29
Switzerland	1.16	1.02	1.31	1.05	1.29

```
lb <- c(1, 1.2, 1.5, 2)
lbl <- c("1", "1.2", "1.5", "2")
ci.beta %>%
  filter(Feature == "Nationality: French") %>%
  group_by(Country) %>%
  reframe(getci(median)) %>%
  ungroup() %>%
  mutate(Mean = exp(Mean), low = exp(low), high = exp(high)) %>%
  mutate(Low = exp(Low), High = exp(High)) %>%
  ggplot(aes(x = Mean, y = Country, color = Country)) +
  geom_vline(xintercept = 1, lty = 3) +
  geom_point(size = 1.2, position = position_dodge(width = 0.2)) +
  geom_linerange(aes(xmin = low, xmax = high),
                 alpha = 0.5,
                 position = position_dodge(width = 0.2)) +
  geom_linerange(aes(xmin = Low, xmax = High),
                 alpha = 0.5, size = 1,
                 position = position_dodge(width = 0.2)) +
  scale_color_manual(values = palette.countries) +
  coord_trans(x = "log10") +
  scale_x_continuous(breaks = lb, labels = lbl) +
  xlab("Odds") +
  theme(legend.position = "bottom")
```

3.2 *H2(a): Sedentary and nationalist norms increase discrimination based on nationality*

The higher the:

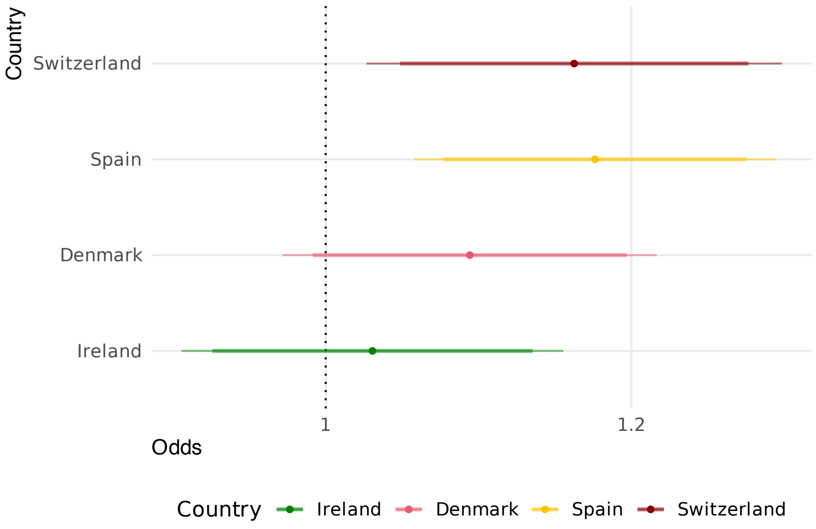

Figure 3.2: Odds of favouring French profiles, by country.

- Anti-immigration attitudes
- Right-wing ideology
- Not migrant status

... the higher the discrimination based on nationality.

```
ci.beta %>%
  filter(Feature == "Nationality: French") %>%
  ggplot(aes(x = median, y = factor(`Ideology (continuous)`), , )) +
#  ggplot(aes(x = median, y = factor(Ideology), , fill = factor(stat(quantile)))) +
#  coord_flip() +
  geom_density_ridges(quantile_lines = TRUE, quantiles = 2)
```

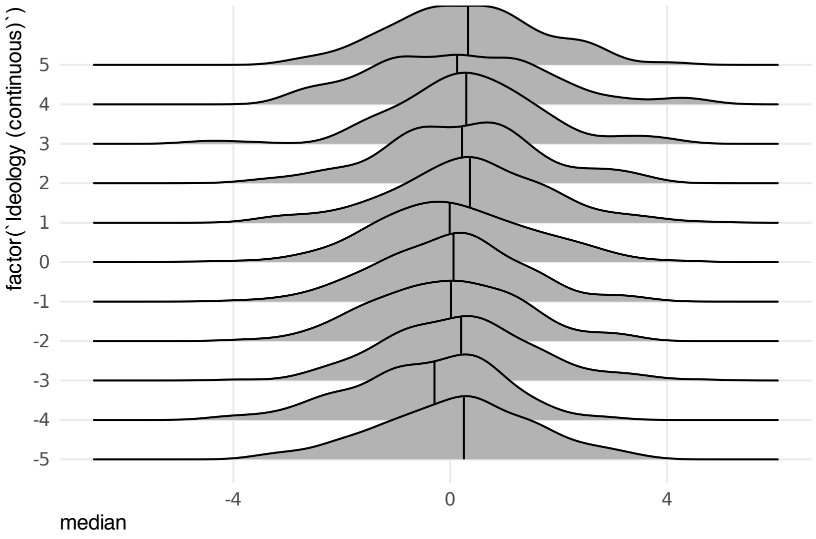

Figure 3.3: Individual utilities distribution for discrimination based on nationality, by Ideology.

```
#  stat_density_ridges(
#    geom = "density_ridges_gradient",
#    calc_ecdf = TRUE,
```

```r
#     quantiles = c(0.025, 0.975)
#   ) +
#   scale_fill_manual(
#     name = "Probability", values = c("#FF0000A0", "#A0A0A0A0", "#0000FFA0"),
#     labels = c("(0, 0.025]", "(0.025, 0.975]", "(0.975, 1]")
#   )

ci.beta %>%
  filter(Feature == "Nationality: French") %>%
  ggplot(aes(x = median, y = factor(`Ideology (binary)`))) +
  geom_density_ridges(quantile_lines = TRUE, quantiles = 2)
```

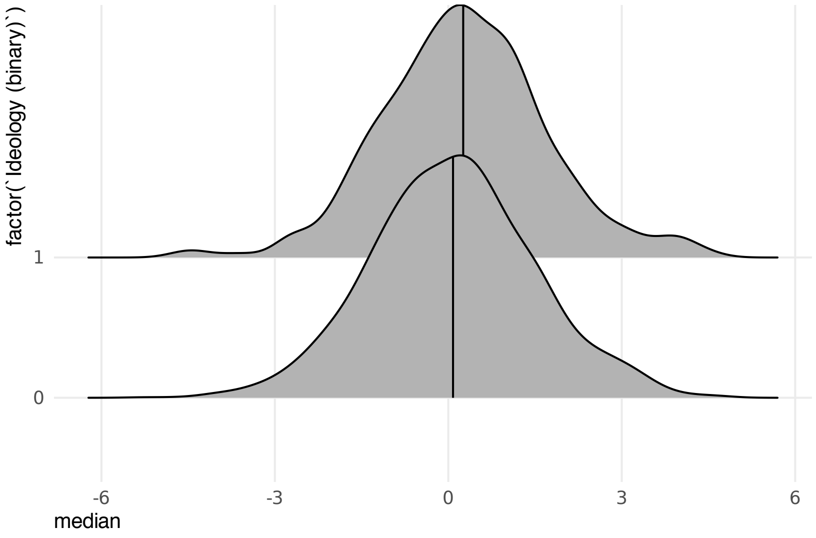

Figure 3.4: Individual utilities distribution for discrimination based on nationality, by right-wing ideology.

```r
ci.beta %>%
  filter(Feature == "Nationality: French") %>%
  ggplot(aes(x = median, y = factor(`Immigration openess`))) +
  geom_density_ridges(quantile_lines = TRUE, quantiles = 2)

ci.beta %>%
  filter(Feature == "Nationality: French") %>%
  ggplot(aes(x = median, y = Migration)) +
  geom_density_ridges(quantile_lines = TRUE, quantiles = 2)

set.seed(14718)
d.model <- ci.beta %>%
  filter(Feature == "Nationality: French") %>%
  rename(y = median)
m.ideology <- stan_glm(y ~ `Ideology (continuous)`, data = d.model,
              family = gaussian())
m.rw.ideology <- stan_glm(y ~ `Ideology (binary)`, data = d.model,
              family = gaussian())
m.attitudes <- stan_glm(y ~ Openess,
```

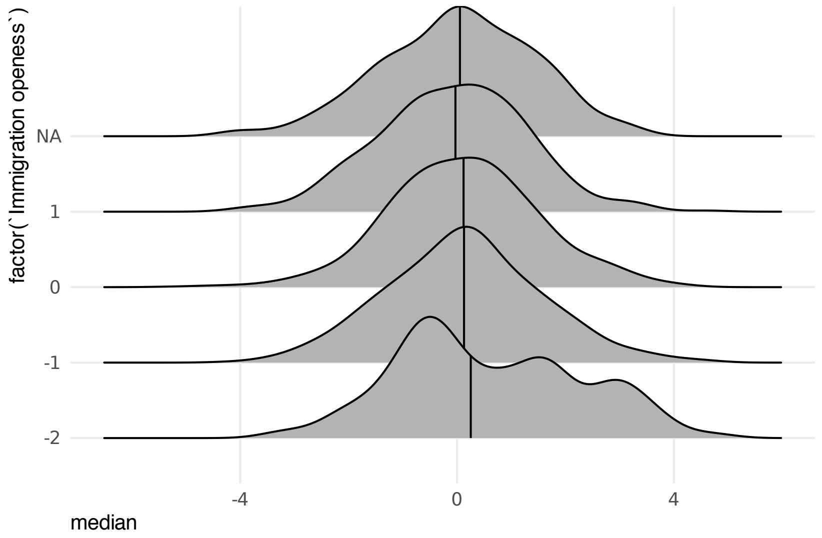

Figure 3.5: Individual utilities distribution for discrimination based on nationality, by immmigration openess.

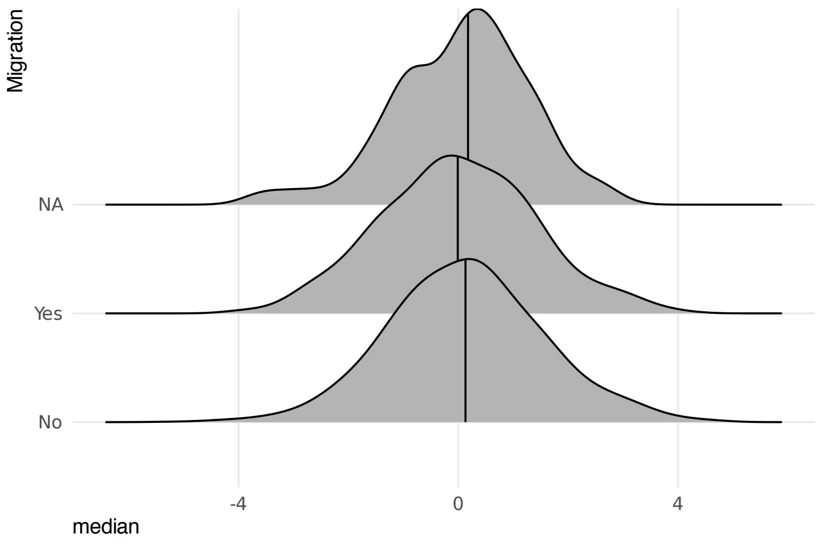

Figure 3.6: Individual utilities distribution for discrimination based on nationality, by migration background.

```
                      data = na.omit(d.model %>%
                                     select(y, `Immigration openess`) %>%
                                     rename(Openess = `Immigration openess`)),
             family = gaussian())
m.attitudes.exclusive ← stan_glm(y ~ `Exclusive immigration attitude`,
                      data = na.omit(d.model %>%
                                     select(y, `Exclusive immigration attitude`)),
             family = gaussian())
m.migration ← stan_glm(y ~ Migration, data = d.model,
             family = gaussian())

→ in 2:                 0.158 seconds (Sampling)
→ Chain 2:                 0.17 seconds (Total)
→ Chain 2:
→ Chain 3: Iteration: 2000 / 2000 [100%]  (Sampling)
→ Chain 3:
→ Chain 3:  Elapsed Time: 0.013 seconds (Warm-up)
→ Chain 3:                0.161 seconds (Sampling)
→ Chain 3:                0.174 seconds (Total)
→ Chain 3:

# For some strange reason, "Right-wing ideology" is a valid name for a variable
# but not "Immigration openess", and therefore we need to do a rename()
# and call it "Openess" only, instead.
#
modelsummary(list("Ideology (continuous)" = m.ideology,
                  "Ideology (binary)" = m.rw.ideology,
                  "Immigration openess" = m.attitudes,
                  "Migration background" = m.migration),
             title = "Simple models on individual discriminations prefering French over Bulgarian profiles.
             metrics = c("R2"), statistic = "conf.int") # RMSE


modelsummary(list("Ideology (continuous)" = m.ideology,
                  "Ideology (binary)" = m.rw.ideology,
                  "Exclusive immigration attitude" = m.attitudes.exclusive),
             title = "Simple linear regression models on individual discriminations prefering French over B
             escape = FALSE,
             metrics = "R2",
#             gof_map = c("nobs", "r.squared"),
             statistic = "conf.int",
  #output = "table-p7-h2.html")
  output = "table-p7-h2.docx")

s.models.all ← bind_rows(
  tidy(m.ideology, conf.int = TRUE) %>%
    mutate(Model = "Ideology (continuous)"),
  tidy(m.rw.ideology, conf.int = TRUE) %>%
    mutate(Model = "Ideology (binary)"),
  tidy(m.attitudes.exclusive, conf.int = TRUE) %>%
    mutate(Model = "Exclusive immigration attitude"))#,
```

Table 3.4: Simple models on individual discriminations prefering French over Bulgarian profiles.

	Ideology (continuous)	Ideology (binary)	Immigration openess	Migration background
(Intercept)	0.110	0.082	0.082	0.133
	[0.056, 0.165]	[0.021, 0.141]	[0.019, 0.144]	[0.070, 0.197]
‘Ideology (continuous)‘	0.041			
	[0.016, 0.066]			
‘Ideology (binary)‘		0.194		
		[0.019, 0.372]		
Openess			−0.136	
			[−0.210, −0.061]	
MigrationYes				−0.143
				[−0.286, 0.002]
Num.Obs.	2403	2403	2243	2350
R2	0.004	0.002	0.006	0.002
Log.Lik.	−4248.999	−4251.724	−3964.509	−4165.111

```
#  tidy(m.migration, conf.int = TRUE) %>%
#    mutate(Model = "Migration background"))

s.models.all %>%
  filter(term ≠ "(Intercept)") %>%
  ggplot(aes(x = estimate, y = Model)) +
  geom_point(size = 1.2) +
  geom_linerange(aes(xmin = conf.low, xmax = conf.high), alpha = 0.5)
```

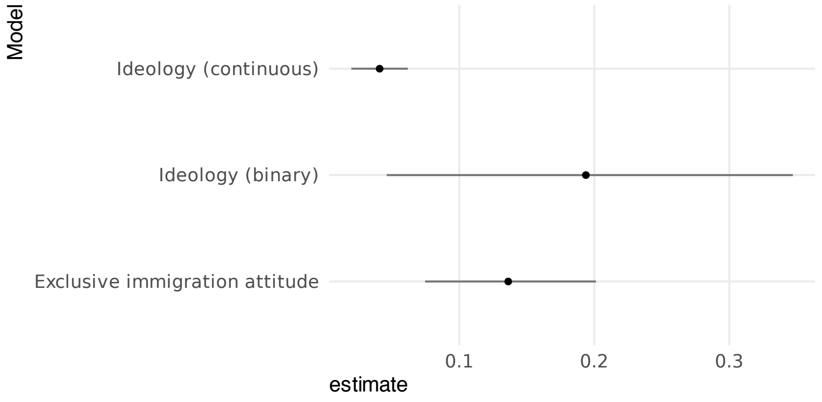

Figure 3.7: Sedentary and nationalist norms increase discrimination based on nationality.

```
### FIGURE 2
lb ← c(1, 1.2, 1.5, 2)
lbl ← c("1", "1.2", "1.5", "2")

s.models.all %>%
  filter(term ≠ "(Intercept)") %>%
```

```
  ggplot(aes(x = exp(estimate), y = Model)) +
  geom_vline(xintercept = 1, lty = 3) +
  geom_point(size = 2) +
  geom_linerange(aes(xmin = exp(conf.low), xmax = exp(conf.high)), alpha = 0.5, size = 1) +
  xlab("Odds (French over Bulgarian)") +
  expand_limits(x = 1)
```

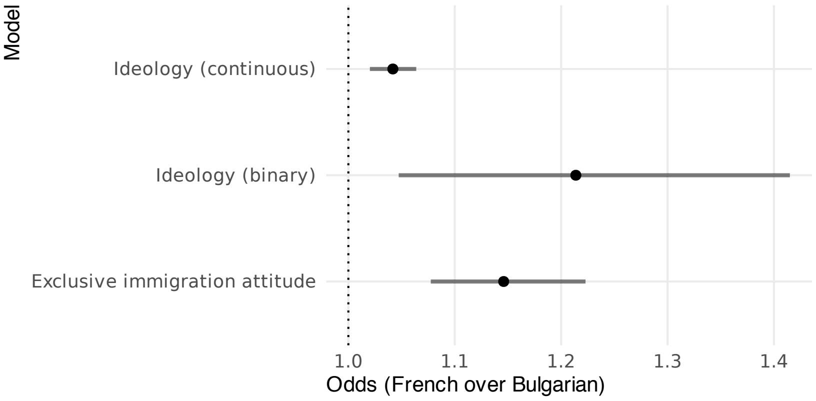

Figure 3.8: Sedentary and nationalist norms increase discrimination based on nationality (favouring French profiles over Bulgarian).

```
# Report results in terms of odds.
ci.beta %>%
  filter(Feature == "Nationality: French") %>%
  reframe(getci.or(median, factor(`Ideology (binary)`))) %>%
  mykbl("Average odds of discrimination favouring French over Bulgarian citizens. Rewrite of basic model ap
```

Table 3.5: Average odds of discrimination favouring French over Bulgarian citizens. Rewrite of basic model approach using getci.or().

OR	or.low	or.high	or.Low	or.High	pc	Prob
1.22	1.02	1.46	1.04	1.41	21.92	0.02

3.3 *H2(b): The pattern in H2(a) varies by country*

```
set.seed(14718)
d.model ← ci.beta %>%
  filter(Feature == "Nationality: French") %>%
  rename(y = median)
m.ideology ← stan_glm(y ~ `Ideology (continuous)` * Country, data = d.model,
            family = gaussian())
m.rw.ideology ← stan_glm(y ~ `Ideology (binary)` * Country, data = d.model,
            family = gaussian())
```

```
→ arm-up)
→ Chain 3:                0.19 seconds (Sampling)
→ Chain 3:                0.223 seconds (Total)
→ Chain 3:
→ Chain 1: Iteration: 2000 / 2000 [100%]  (Sampling)
→ Chain 1:
```

```
→ Chain 1:  Elapsed Time: 0.033 seconds (Warm-up)
→ Chain 1:                0.193 seconds (Sampling)
→ Chain 1:                0.226 seconds (Total)
→ Chain 1:
```

```
m.attitudes ← stan_glm(y ~ Openess * Country,
                        data = na.omit(d.model %>%
                                       select(y, `Immigration openess`, Country) %>%
                                       rename(Openess = `Immigration openess`)),
              family = gaussian())
```

```
→
→ arm-up)
→ Chain 3:                0.19 seconds (Sampling)
→ Chain 3:                0.223 seconds (Total)
→ Chain 3:
→ Chain 1: Iteration: 2000 / 2000 [100%]  (Sampling)
→ Chain 1:
→ Chain 1:  Elapsed Time: 0.033 seconds (Warm-up)
→ Chain 1:                0.193 seconds (Sampling)
→ Chain 1:                0.226 seconds (Total)
→ Chain 1:
```

```
m.attitudes.exclusive ← stan_glm(y ~ `Exclusive immigration attitude` * Country,
                        data = na.omit(d.model %>%
                                       select(y, `Exclusive immigration attitude`, Country)),
              family = gaussian())
```

```
→
→
→ arm-up)
→ Chain 3:                0.19 seconds (Sampling)
→ Chain 3:                0.223 seconds (Total)
→ Chain 3:
→ Chain 1: Iteration: 2000 / 2000 [100%]  (Sampling)
→ Chain 1:
→ Chain 1:  Elapsed Time: 0.033 seconds (Warm-up)
→ Chain 1:                0.193 seconds (Sampling)
→ Chain 1:                0.226 seconds (Total)
→ Chain 1:
```

```
m.migration ← stan_glm(y ~ Migration * Country, data = d.model,
              family = gaussian())
```

```
→
→ arm-up)
→ Chain 3:                0.19 seconds (Sampling)
→ Chain 3:                0.223 seconds (Total)
→ Chain 3:
→ Chain 1: Iteration: 2000 / 2000 [100%]  (Sampling)
→ Chain 1:
```

```
→ Chain 1:  Elapsed Time: 0.033 seconds (Warm-up)
→ Chain 1:                0.193 seconds (Sampling)
→ Chain 1:                0.226 seconds (Total)
→ Chain 1:
```

```
s.models.countries ← bind_rows(
  tidy(m.ideology, conf.int = TRUE) %>%
    mutate(Model = "Ideology (continuous)"),
  tidy(m.rw.ideology, conf.int = TRUE) %>%
    mutate(Model = "Ideology (binary)"),
  tidy(m.attitudes.exclusive, conf.int = TRUE) %>%
    mutate(Model = "Exclusive immigration attitude")) %>%
#  tidy(m.migration, conf.int = TRUE) %>%
#    mutate(Model = "Migration background")) %>%
  mutate(term = ifelse(term == "`Ideology (continuous)`", "`Ideology (continuous)`:CountryIreland", term))
  mutate(term = ifelse(term == "MigrationYes", "CountryIreland:MigrationYes", term)) %>%
  mutate(term = ifelse(term == "`Exclusive immigration attitude`", "`Exclusive immigration attitude`:Countr
  mutate(term = ifelse(term == "`Ideology (binary)`", "`Ideology (binary)`:CountryIreland", term)) %>%
  mutate(Country = str_replace(term, "Country", "")) %>%
  mutate(Country = str_replace(Country, "^.+:", "")) %>%
  mutate(Covariate = ifelse(str_detect(term, ":"), "Covariate", "Intercept")) %>%
  mutate(Country = ifelse(Country == "(Intercept)", "Ireland", Country))

s.models.countries %>%
  filter(Model %in% c("Ideology (continuous)",
                      "Ideology (binary)",
                      "Exclusive immigration attitude")) %>%
  filter(term ≠ "(Intercept)") %>%
  filter(Covariate == "Covariate") %>%
  ggplot(aes(x = estimate, y = Model, color = Country)) +
  geom_vline(xintercept = 0, lty = 3) +
  geom_point(size = 1.2, position = position_dodge(width = 0.2)) +
  geom_linerange(aes(xmin = conf.low, xmax = conf.high),
               alpha = 0.5,
               position = position_dodge(width = 0.2)) +
  scale_color_manual(values = palette.countries) +
  xlab("HPD")


s.models.countries %>%
#  filter(term ≠ "(Intercept)") %>%
  filter(Covariate == "Intercept") %>%
  ggplot(aes(x = estimate, y = Model, color = Country)) +
  geom_vline(xintercept = 0, lty = 3) +
  geom_point(size = 1.2, position = position_dodge(width = 0.2)) +
  geom_linerange(aes(xmin = conf.low, xmax = conf.high),
               alpha = 0.5,
               position = position_dodge(width = 0.2)) +
  scale_color_manual(values = palette.countries) +
  ggtitle("(Intercepts)")
```

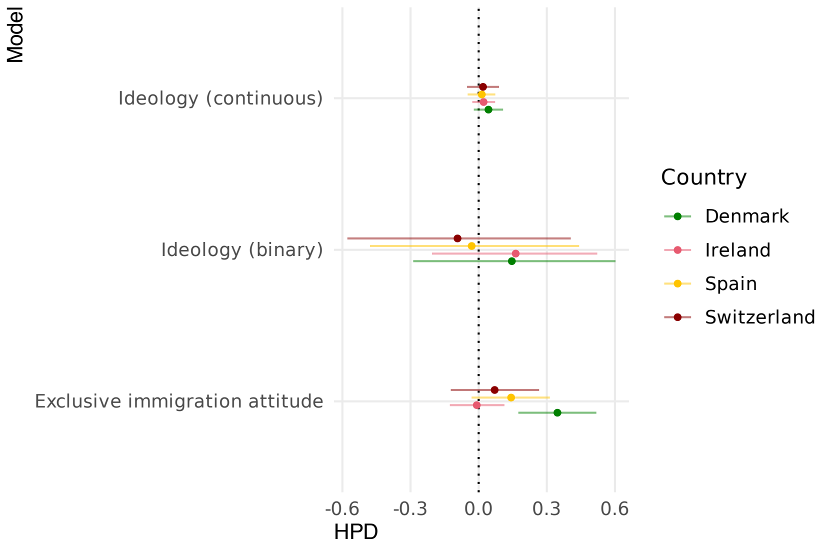

Figure 3.9: Sedentary and nationalist norms increase discrimination based on nationality, by country.

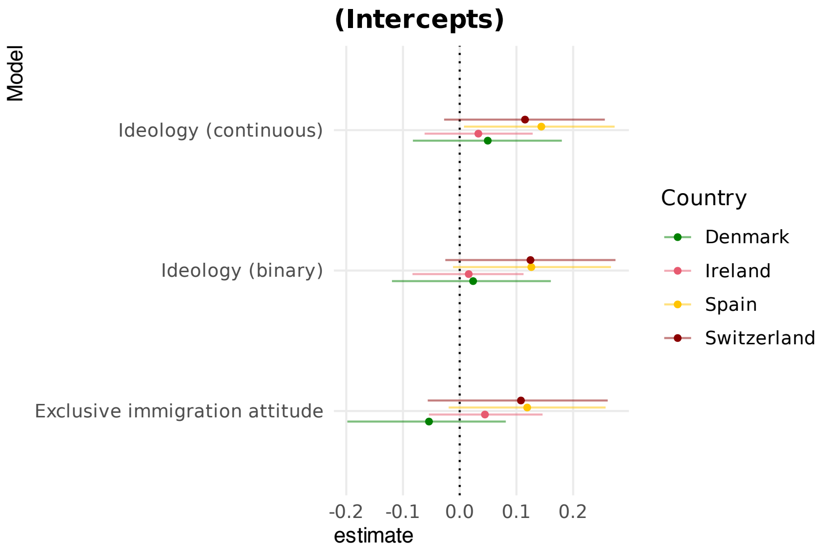

Figure 3.10: Country intercepts of discrimination based on nationality.

```
s.models.and.all <- bind_rows(s.models.countries,
                              s.models.all %>%
                                mutate(Country = "(All)"))

s.models.and.all %>%
  filter(term != "(Intercept)") %>%
  filter(Covariate == "Covariate" | Country == "(All)") %>%
  ggplot(aes(x = exp(estimate), y = Model, color = Country)) +
  geom_vline(xintercept = 1, lty = 3) +
  geom_point(size = 2, position = position_dodge(width = 0.2)) +
  geom_linerange(aes(xmin = exp(conf.low), xmax = exp(conf.high)),
                 alpha = 0.5,
                 size = 1,
                 position = position_dodge(width = 0.2)) +
  xlab("Odds (French over Bulgarian)") +
  scale_color_manual(values = c("black", palette.countries))
```

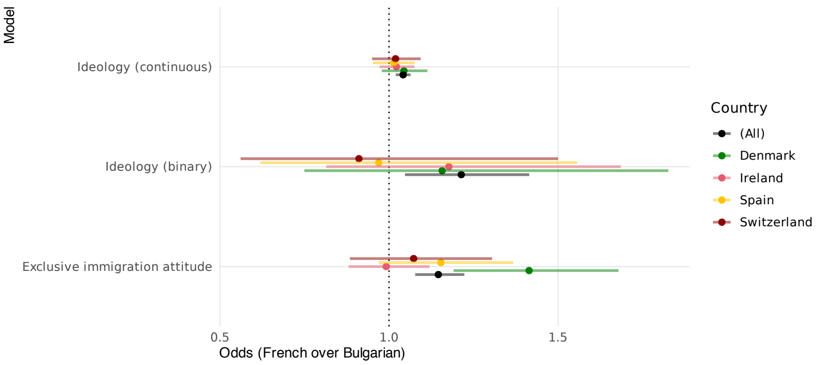

Figure 3.11: Sedentary and nationalist norms increase discrimination based on nationality (favouring French profiles over Bulgarian).

3.4 *H3: The higher the effort, the lowest the discrimination*

Effort as considering: - Language - Application for jobs

```
ci.beta %>%
  filter(Feature == "Language: Fluent") %>%
  mutate(Odds = exp(median)) %>%
  ggplot(aes(x = Odds)) +
  geom_histogram() +
  scale_x_continuous(trans = "log2") +
  geom_vline(xintercept = 1, lty = 3)

#ci.beta %>%
#  filter(Feature == "Language: Fluent") %>%
#  summarize(P = length(which(median > 0)) / n()) %>%
#  mykbl("Probability of the evidence of discrimination favouring fluent language profiles.")

ci.beta %>%
```

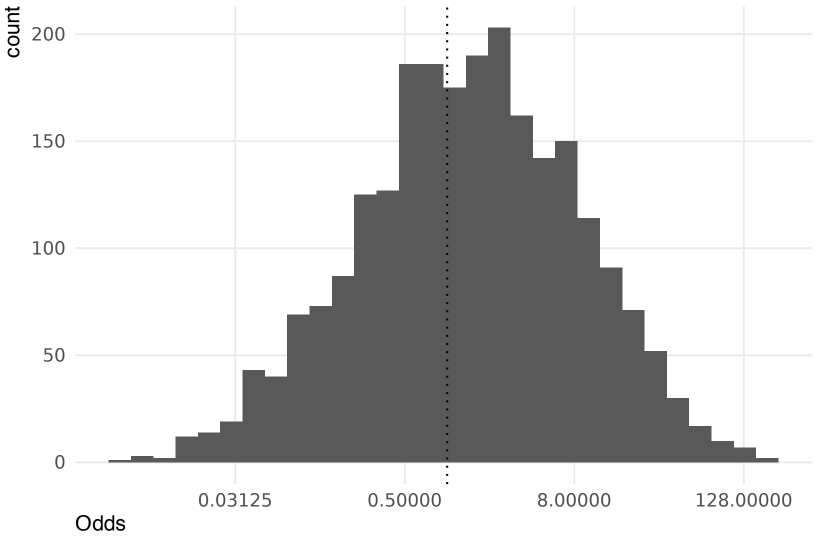

Figure 3.12: Individual utilities for prioritization of profiles with fluent language capacity.

```
  filter(Feature == "Language: Fluent") %>%
  reframe(getci(median)) %>%
  pivot_longer(everything(), names_to = "Variable", values_to = "value") %>%
  mutate(value = exp(value)) %>%
  pivot_wider(names_from = Variable, values_from = value) %>%
  mykbl("Average odds of discrimination favouring profiles with fluent language.", digits = 3)
```

Table 3.6: Average odds of discrimination favouring profiles with fluent language.

Mean	low	high	Low	High
1.403	1.307	1.507	1.322	1.49

```
ci.beta %>%
  filter(Feature == "Applications: 5") %>%
  mutate(Odds = exp(median)) %>%
  ggplot(aes(x = Odds)) +
  geom_histogram() +
  scale_x_continuous(trans = "log2") +
  geom_vline(xintercept = 1, lty = 3)
```

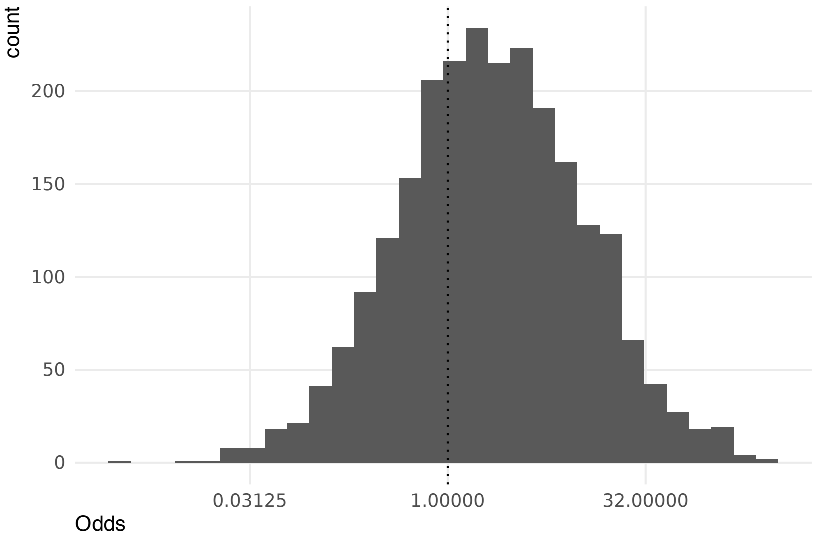

Figure 3.13: Individual utilities for prioritization of profiles with more job applications.

```
#ci.beta %>%
#   filter(Feature == "Applications: 5") %>%
#   summarize(P = length(which(median > 0)) / n()) %>%
```

Table 3.7: Average odds of discrimination favouring profiles with 5 work applications.

Mean	low	high	Low	High
2.134	2.001	2.277	2.022	2.253

3.5 H4: Efforts vs. Identity matter differently for different types of individuals

Importance is the proportion of discrimination that each individual assigns to each feature.

We calculate the importance of each feature by calculating the proportion that the absolute value of each individuals' utility represents against all the utilities.

```
d.importance <- ci.beta %>%
  filter(Feature %in% order.feature.variables) %>%
  droplevels() %>%
  # Calculate importance for each feature
  group_by(id) %>%
  mutate(Importance = abs(median) / sum(abs(median))) %>%
  ungroup() %>%
  # Group features in two types
  mutate(Type = case_when(
    Feature == "Nationality: French" ~ "Identity",
    Feature %in% c("Language: Fluent", "Applications: 5") ~ "Effort",
    TRUE ~ NA_character_)) %>%
  filter(!is.na(Type)) %>%
  # Importance is now the sum of the types of features
  group_by(id, Country, Type,
         Migration,
         `Exclusive immigration attitude`,
         `Ideology (continuous)`,
         `Ideology (binary)`) %>%
  summarize(Importance = sum(Importance)) %>%
  ungroup() %>%
  pivot_wider(names_from = Type, values_from = Importance)

d.importance %>%
  ggplot(aes(x = Identity, y = Effort)) +
  geom_point() +
  scale_x_continuous(labels = percent_format()) +
  scale_y_continuous(labels = percent_format())
```

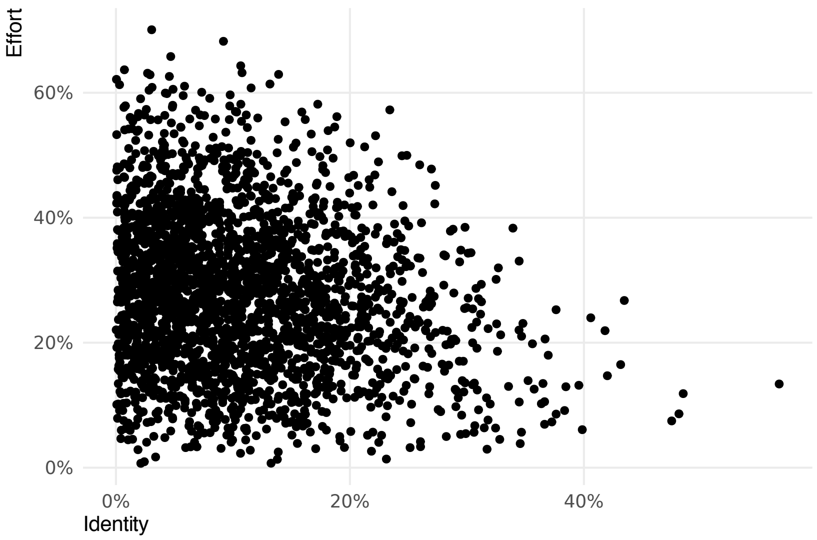


```r
d.importance %>%
  ggplot(aes(x = Identity, y = Effort, color = Country)) +
  geom_point(alpha = 0.5) +
  scale_x_continuous(labels = percent_format()) +
  scale_y_continuous(labels = percent_format()) +
  geom_smooth(se = FALSE, alpha = 0.5) +
  scale_color_manual(values = palette.countries)# +
```

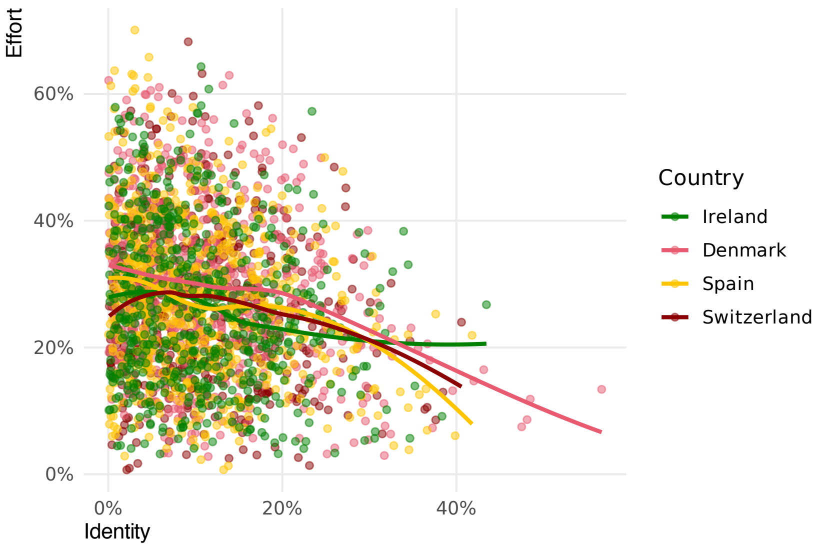

Figure 3.14: Importance by country.

```r
#  facet_wrap(~ Country)

d.importance %>%
  ggplot(aes(x = Identity, y = Effort, color = Country)) +
  geom_point(alpha = 0.5) +
  scale_x_continuous(labels = percent_format()) +
  scale_y_continuous(labels = percent_format()) +
  geom_smooth(se = FALSE, alpha = 0.5) +
```

```
  scale_color_manual(values = palette.countries) +
  facet_wrap(~ Country)
```

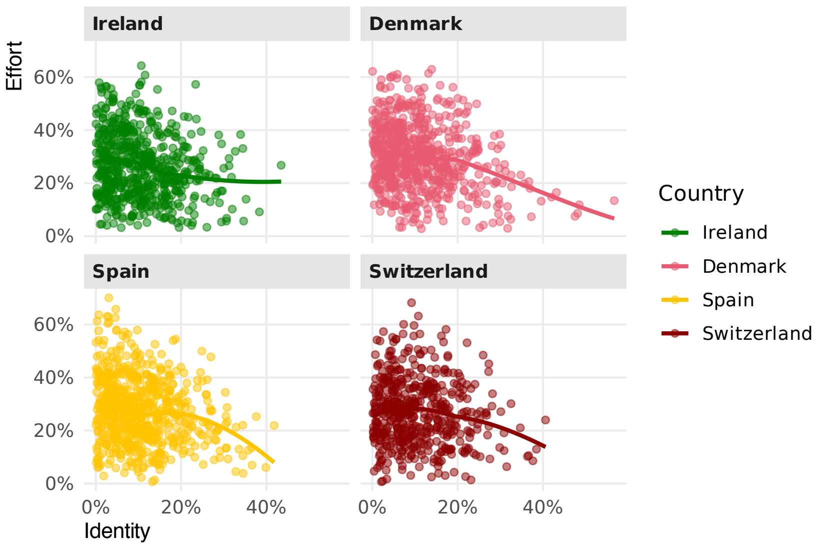

Figure 3.15: Importance by country.

```
d.importance %>%
  ggplot(aes(x = Identity, y = Effort)) +
  geom_point(alpha = 0.5) +
  scale_x_continuous(labels = percent_format()) +
  scale_y_continuous(labels = percent_format()) +
  #geom_smooth(method = "lm", se = FALSE, alpha = 0.5) +
  geom_smooth(se = FALSE, alpha = 0.5) +
  facet_wrap(~ `Ideology (continuous)`)
```

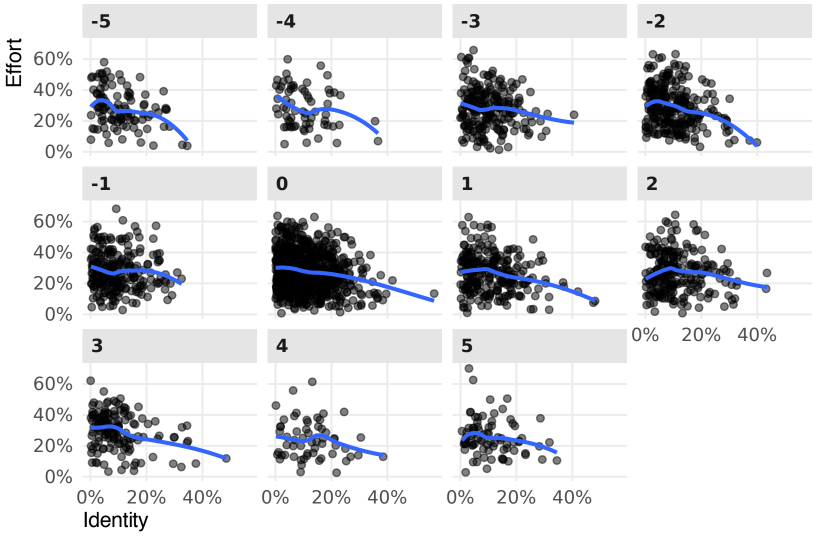

Figure 3.16: Importance by Ideology.

```
d.importance %>%
  ggplot(aes(x = Identity, y = Effort)) +
```

```
  geom_point(alpha = 0.5) +
  scale_x_continuous(labels = percent_format()) +
  scale_y_continuous(labels = percent_format()) +
  geom_smooth(se = FALSE, alpha = 0.5) +
  facet_wrap(~ `Ideology (binary)`)
```

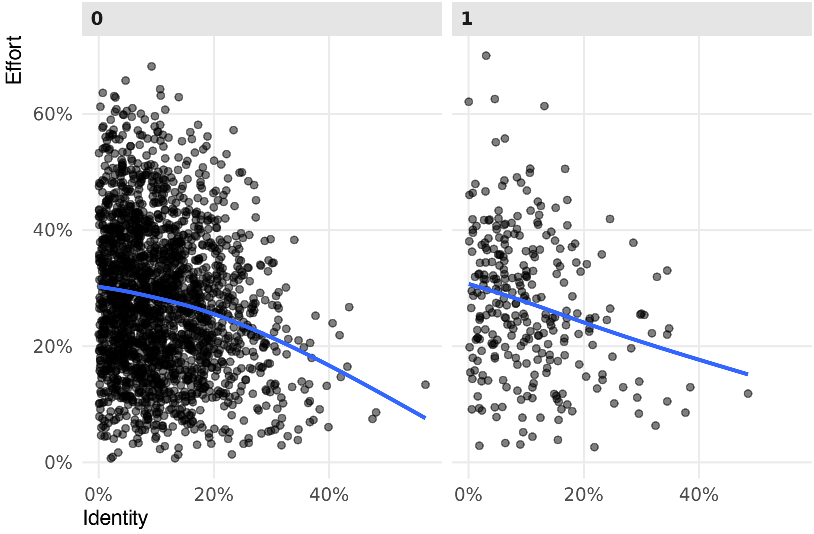

Figure 3.17: Importance by right-wing ideology.

```
d.importance %>%
  ggplot(aes(x = Identity, y = Effort)) +
  geom_point(alpha = 0.5) +
  scale_x_continuous(labels = percent_format()) +
  scale_y_continuous(labels = percent_format()) +
  geom_smooth(se = FALSE, alpha = 0.5) +
  facet_wrap(~ `Exclusive immigration attitude`)
```

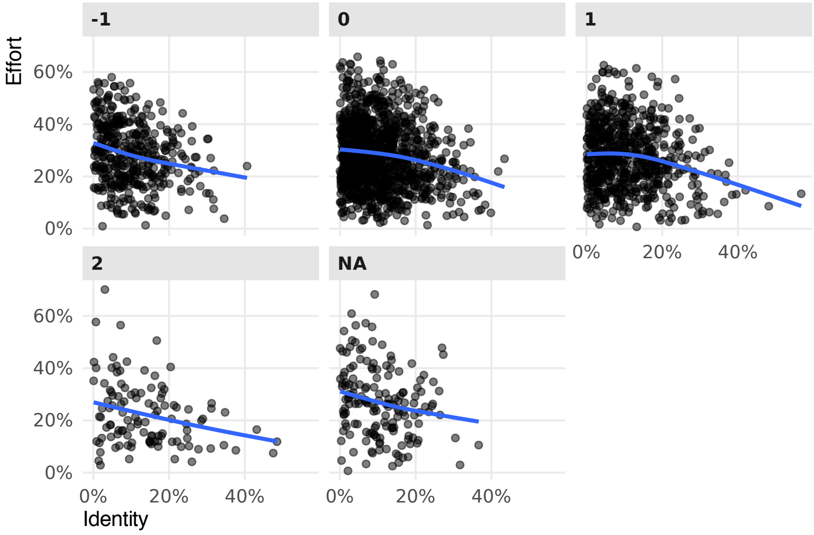

Figure 3.18: Importance by openess to immigration.

```
d.importance %>%
  ggplot(aes(x = Identity, y = Effort)) +
  geom_point(alpha = 0.5) +
  scale_x_continuous(labels = percent_format()) +
  scale_y_continuous(labels = percent_format()) +
  geom_smooth(se = FALSE, alpha = 0.5) +
  facet_wrap(~ Migration)
```

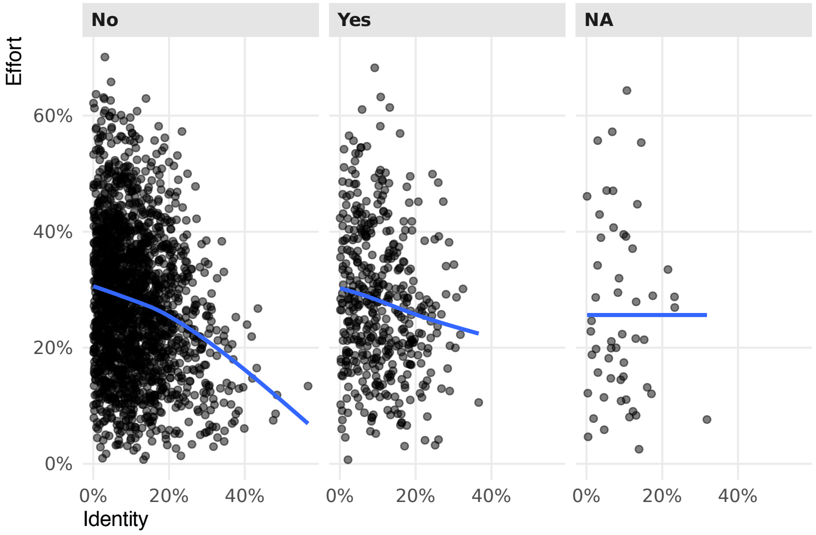

Figure 3.19: Importance by openess to immigration.

3.6 *Relevant figures*

```
order.beta.mean <- ci.beta %>%
 group_by(Feature) %>%
 summarize(Mean = mean(median)) %>%
 arrange(desc(Mean)) %>%
 mutate(Feature = as.character(Feature)) %>%
 select(Feature) %>%
 unlist(use.names = FALSE)

ci.beta.bins <- ci.beta %>%
  mutate(interval = cut(median, breaks = seq(-8, 8, 0.2))) %>%
  group_by(Feature, interval) %>%
  count() %>%
  mutate(value = ifelse(str_detect(interval, "-"), "Negative", "Positive")) %>%
  ungroup() %>%
  mutate(Feature = factor(as.character(Feature), levels = order.beta.mean)) %>%
  mutate(Preference = factor(ifelse(value == "Positive", "Prioritized", "Neglected"))) %>%
  mutate(Preference = fct_relevel(Preference, "Neglected"))

ci.beta.averages <- ci.beta %>%
  group_by(Feature) %>%
  summarize(Average = mean(median))
```

```
f1 <- ci.beta.bins %>%
  filter(Feature %in% c("Nationality: French", "Applications: 5",
                        "Language: Fluent")) %>%
  mutate(Feature = fct_relevel(Feature, rev(levels(Feature)))) %>%
  ggplot(aes(x = interval, y = n, color = Preference, fill = Preference)) +
  geom_bar(stat = "identity") +
  facet_grid(Feature ~ .) +
  scale_color_manual(values = c("gray40", "gray80")) +
  scale_fill_manual(values = c("gray40", "gray80")) +
#  scale_x_discrete(breaks = c("(-6,-5.8]", "(-4,-3.8]", "(-2,-1.8]", "(-0.2,0]", "(2,2.2]", "(4,4.2]", "(6
#                   labels = as.character(round(exp(seq(-6, 6, by = 2)), digits = 2))) +
  scale_x_discrete(breaks = c("(-4,-3.8]", "(-2,-1.8]", "(-1,-0.8]", "(-0.2,0]", "(1,1.2]", "(2,2.2]", "(4,
                  #labels = as.character(round((c(-4, -2, -1, 0, 1, 2, 4)), digits = 2))) +
                  labels = as.character(c("-4\n(0.02)", "-2\n(0.14)",
                                          "-1\n(0.37)", "0\n(1)", "1\n(2.7)",
                                          "2\n(7.4)", "4\n(55)"))) +
  xlab("Individual utility") + ylab("Number of individuals") +
  theme(legend.position = "bottom")
print(f1)

ci.beta.bins %>%
  filter(Feature %in% c("Nationality: French", "Applications: 5",
                        "Language: Fluent")) %>%
  ggplot(aes(x = interval, y = n, color = Preference, fill = Preference)) +
  geom_bar(stat = "identity") +
  facet_grid(Feature ~ .) +
  scale_color_manual(values = pal.prioritization) +
  scale_fill_manual(values = pal.prioritization) +
#  scale_x_discrete(breaks = c("(-6,-5.8]", "(-4,-3.8]", "(-2,-1.8]", "(-0.2,0]", "(2,2.2]", "(4,4.2]", "(6
#                   labels = as.character(round(exp(seq(-6, 6, by = 2)), digits = 2))) +
  scale_x_discrete(breaks = c("(-4,-3.8]", "(-2,-1.8]", "(-1,-0.8]", "(-0.2,0]", "(1,1.2]", "(2,2.2]", "(4,
                  labels = as.character(round(exp(c(-4, -2, -1, 0, 1, 2, 4)), digits = 2))) +
  xlab("Individual utility (odds)") + ylab("Number of individuals")

f2 <- ci.beta %>%
  filter(Feature == "Nationality: French") %>%
  ggplot(aes(x = jitter(`Ideology (continuous)`), y = median)) +
  geom_point(alpha = 0.5) +
  geom_smooth(method = "lm") +
  xlab("Ideology (continuous)") + ylab("Individual utilities\n(Nationality: French)")


f3 <- ci.beta %>%
  filter(Feature == "Nationality: French") %>%
  ggplot(aes(x = jitter(`Ideology (binary)`), y = median)) +
  geom_point(alpha = 0.5) +
  geom_smooth(method = "lm") +
  xlab("Ideology (binary)") + ylab("Individual utilities\n(Nationality: French)")
```

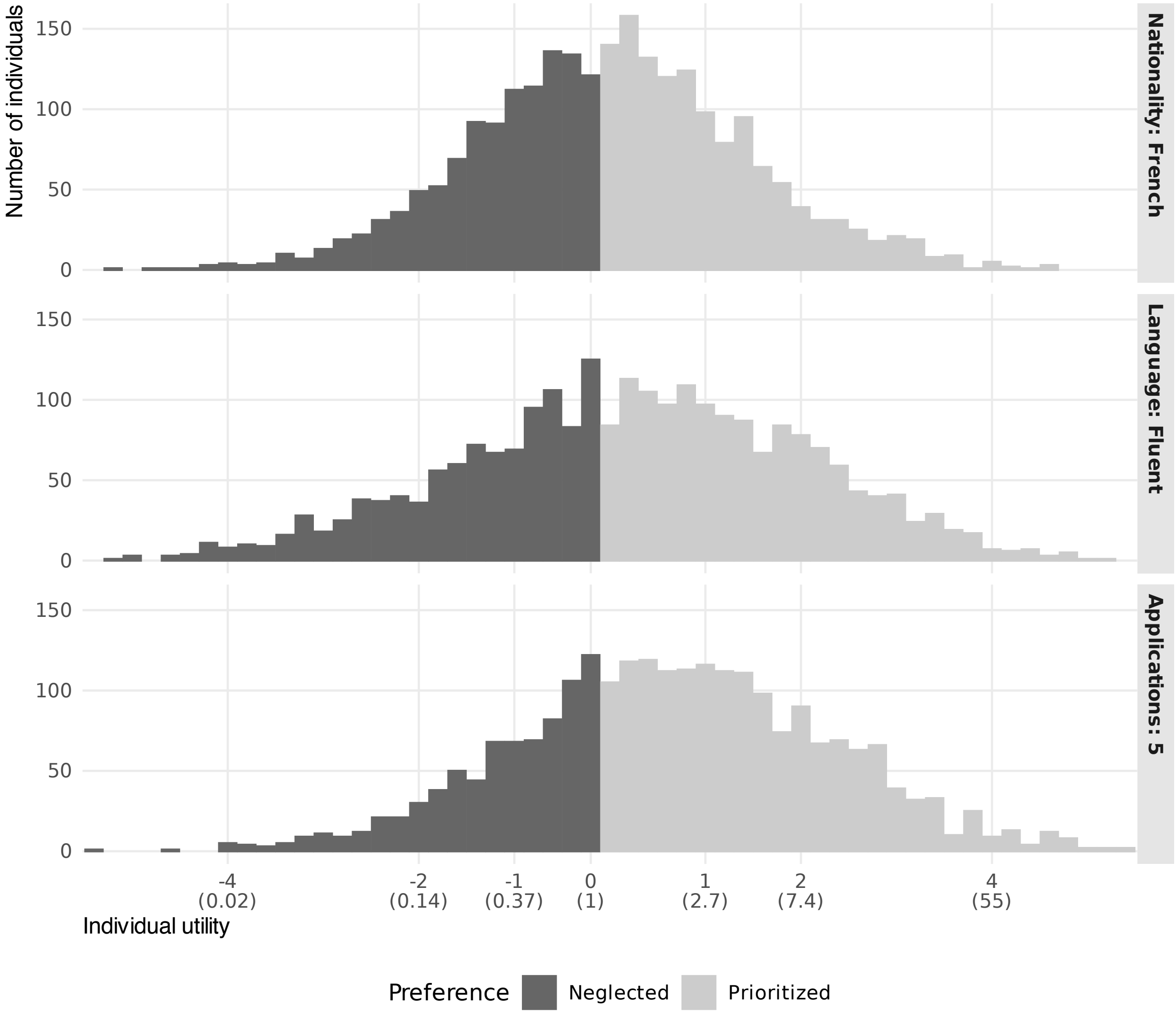

Figure 3.20: Distribution of the mean values for each individuals' utilities. Colors represent positive/negative utilities.

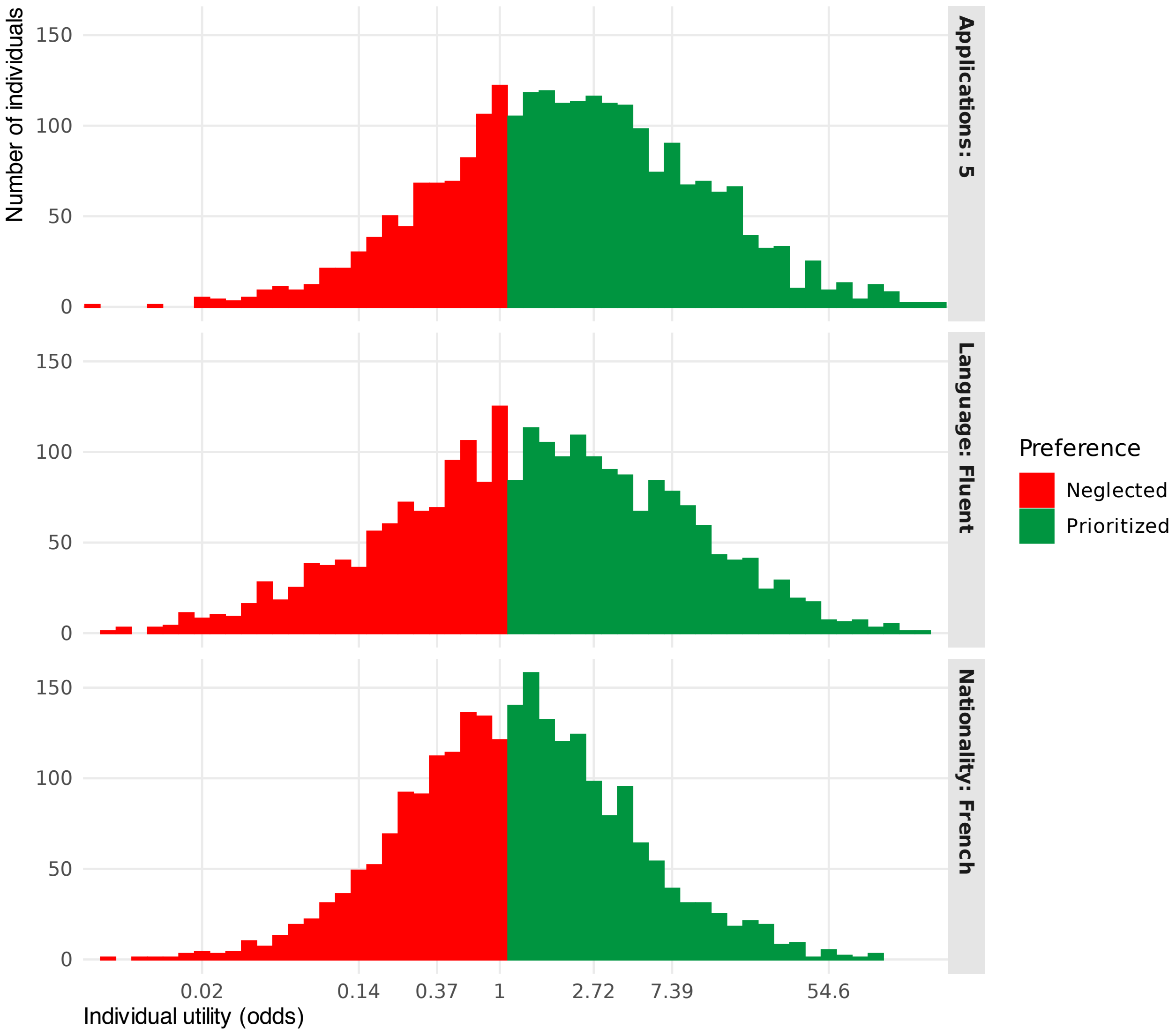

Figure 3.21: Distribution of the mean values for each individuals' utilities (odds). Colors represent positive/negative utilities.

```
f4 <- ci.beta %>%
  filter(Feature == "Nationality: French") %>%
  ggplot(aes(x = jitter(`Exclusive immigration attitude`), y = median)) +
  geom_point(alpha = 0.5) +
  geom_smooth(method = "lm") +
  xlab("Exclusive immigration attitude") + ylab("Individual utilities\n(Nationality: French)")
```

```
cowplot::plot_grid(f1, cowplot::plot_grid(f2, f3, f4, ncol = 1), ncol = 2)
```

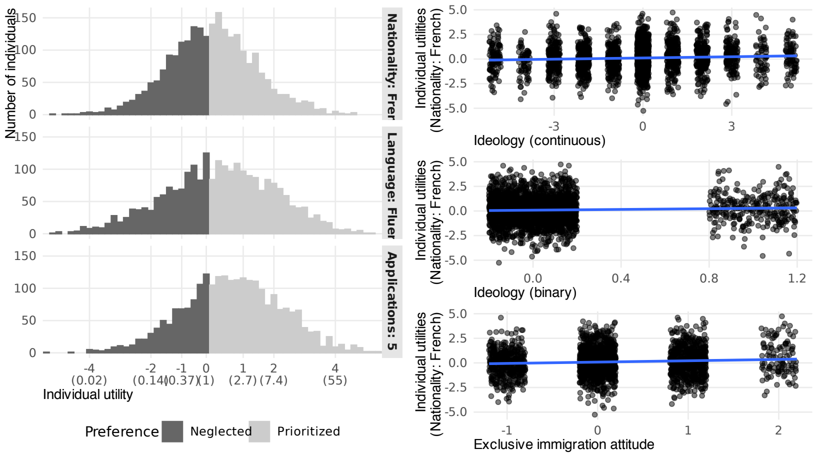

Figure 3.22: Individual utilities with preferences for each individual and attribute.

```
f.grid <- ci.beta %>%
  filter(Feature %in% c("Nationality: French", "Applications: 5",
                        "Language: Fluent")) %>%
  rename(Attribute = Feature) %>%
  select(Attribute, median,
         `Ideology (continuous)`,
         `Ideology (binary)`,
         `Exclusive immigration attitude`) %>%
  pivot_longer(-c(Attribute, median), names_to = "Covariate", values_to =
               "value") %>%
  ggplot(aes(x = jitter(value), y = median)) +
  geom_point(alpha = 0.2) +
  geom_smooth(method = "lm", se = FALSE) +
  facet_grid(Attribute ~ Covariate, scales = "free_x") +
  xlab("") + ylab("Individual utility")
print(f.grid)
```

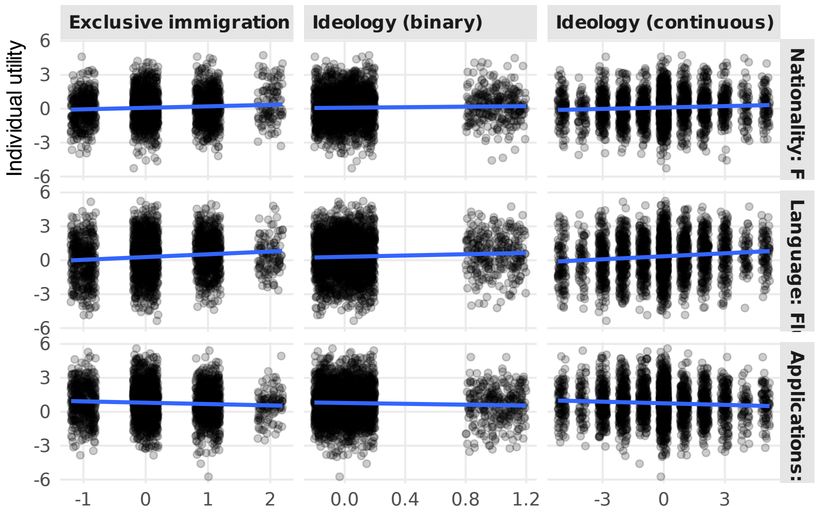


```
### FIGURE 1
cowplot::plot_grid(f1, f.grid, ncol = 2, rel_widths = c(1, 2))

f.grid ← ci.beta %>%
  filter(Feature %in% c("Nationality: French", "Applications: 5",
                        "Language: Fluent")) %>%
  rename(Attribute = Feature) %>%
  select(Country, Attribute, median,
         `Ideology (continuous)`,
         `Ideology (binary)`,
         `Exclusive immigration attitude`) %>%
  pivot_longer(-c(Country, Attribute, median), names_to = "Covariate", values_to =
               "value") %>%
  ggplot(aes(x = jitter(value), y = median, color = Country)) +
  geom_point(alpha = 0.2) +
  geom_smooth(method = "lm", se = FALSE) +
  facet_grid(Attribute ~ Covariate, scales = "free_x") +
  scale_color_manual(values = palette.countries) +
  xlab("") + ylab("Individual utility")
print(f.grid)

### FIGURE 3
ci.beta %>%
  filter(Feature %in% c("Nationality: French",
                        "Language: Fluent",
                        "Applications: 5")) %>%
  group_by(Feature) ▷
  reframe(getci(median)) %>%
  pivot_longer(-Feature, names_to = "Variable", values_to = "value") ▷
  mutate(value = exp(value)) %>%
  pivot_wider(names_from = Variable, values_from = value) %>%
  ggplot(aes(x = Mean, y = Feature)) +
  geom_point(size = 1.2) +
  geom_linerange(aes(xmin = low, xmax = high),
```

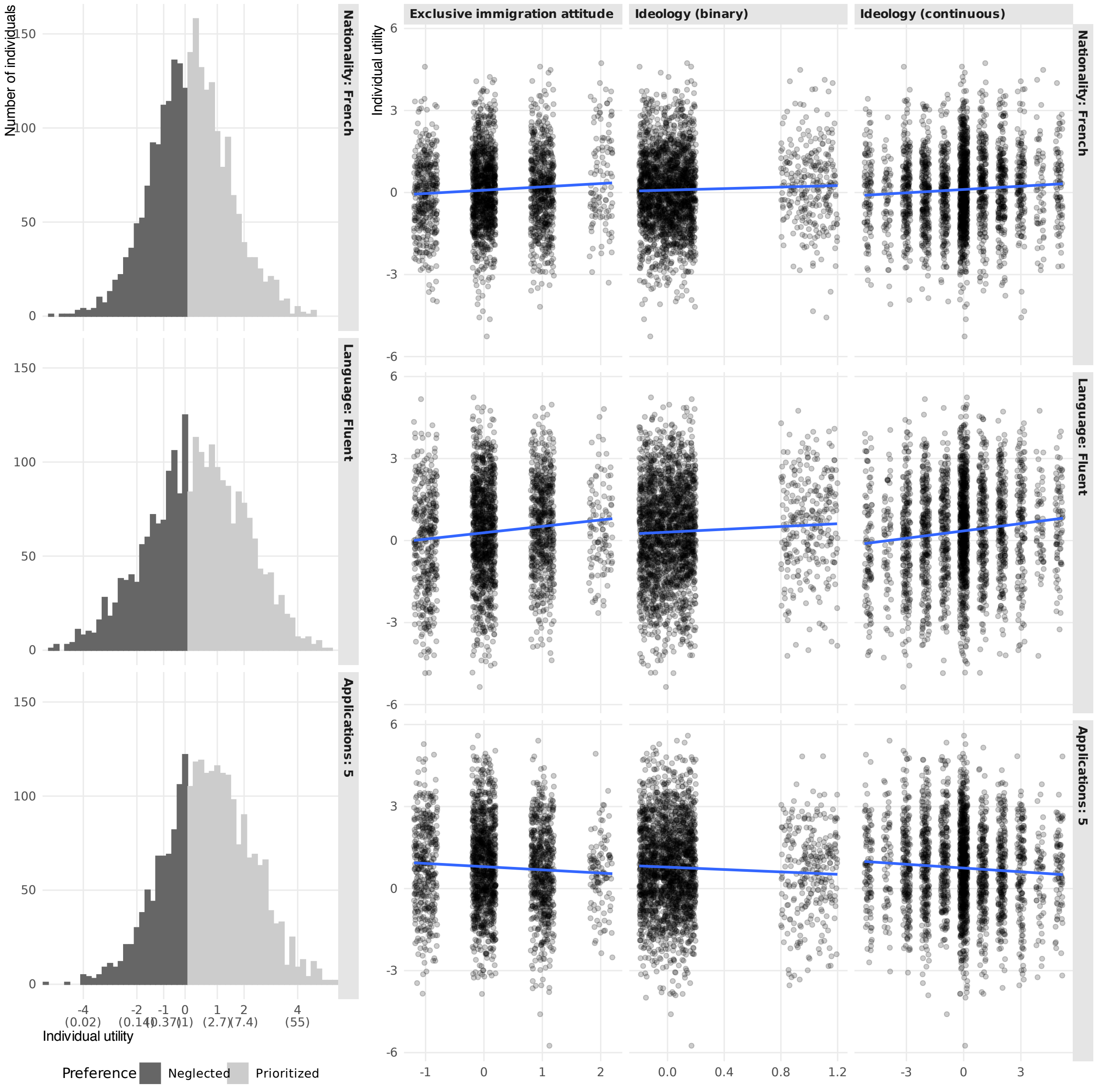

Figure 3.23: Individual utilities with preferences for each individual and attribute.

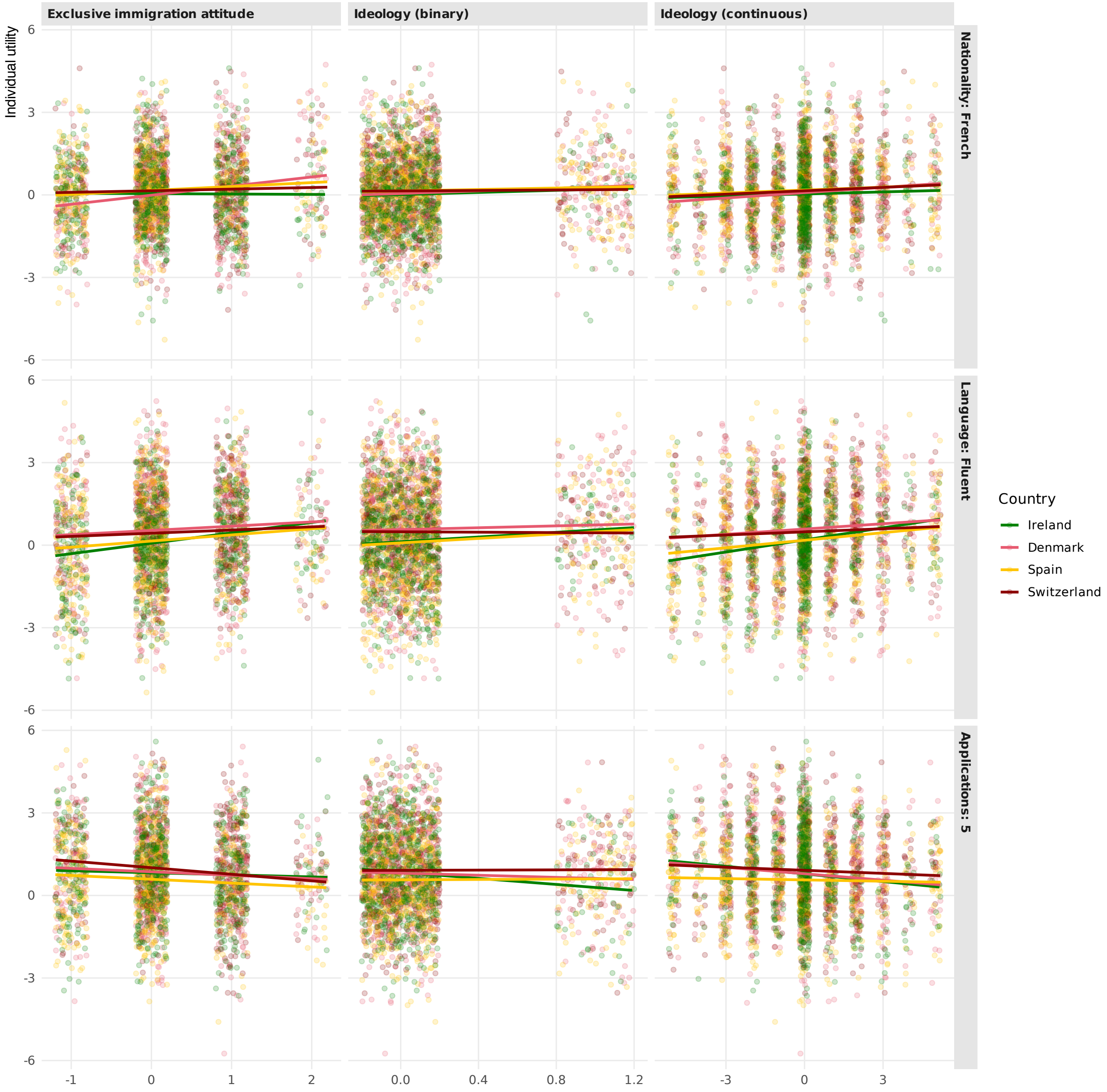

Figure 3.24: Individual utilities with preferences for each individual and attribute, by country.

```
               alpha = 0.5) +
  geom_linerange(aes(xmin = Low, xmax = High),
               alpha = 0.5, size = 1) +
  expand_limits(x = 1) +
  xlab("Odds ratios")
```

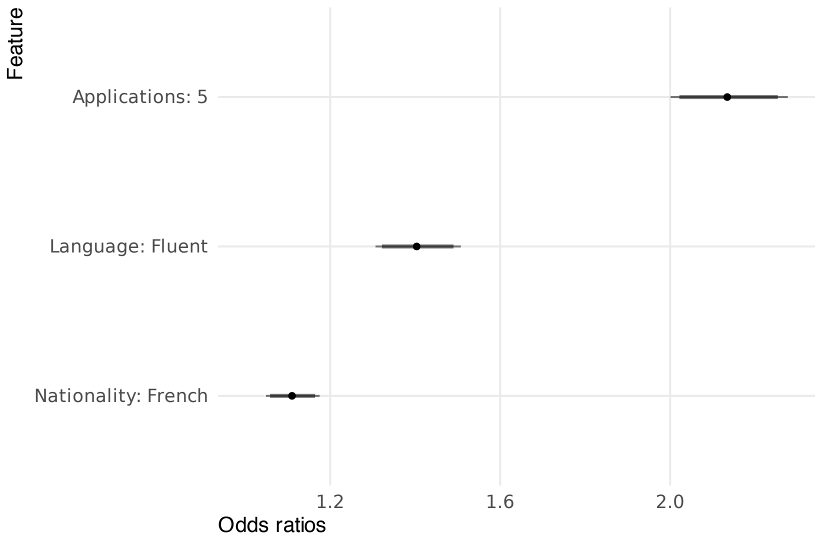

Figure 3.25: Odds of favouring profiles, based on nationality, language ability and applications.

3.7 *Provide classical AMCEs*

Replicate analysis with AMCE reporting for no country difference and country differences. In the no-country differences, also perform a variation to control by ideology.

```
library(cregg)
library(xtable)


######### NO COUNTRY, NO IDEOLOGY
amce <- cj(
           id = ~id,
   data = d,
   formula = Decision ~ Nationality + Gender + Language + Profession + Age +
     Applications + Stay + Shown,
   estimate = "amce",
   )


tc <- "Estimated Average Marginal Component Effects. No country differences."
tb <- amce %>%
  filter(!is.na(std.error)) %>%
  mutate(CI = paste0("[", signif(lower, 2), " : ", signif(upper, 2), "]")) %>%
  select(Feature = feature, Level = level, `AMCE Estimate` = estimate, SE = std.error, `p-value` = p, CI)
```

```
print(xtable(tb,
             caption = tc,
             digits = 4,
             label = "tab:amce_nocountry"),
  file = "tab-p7-amce_nocountry.tex",
  include.rownames = FALSE,
  caption.placement = "top",
  sanitize.text.function = identity)

mykbl(tb, tc, digits = 4)
```

Table 3.8: Estimated Average Marginal Component Effects. No country differences.

Feature	Level	AMCE Estimate	SE	p-value	CI
Nationality	French	0.0126	0.0050	0.0108	[0.0029 : 0.022]
Gender	Male	-0.0393	0.0047	0.0000	[-0.048 : -0.03]
Language	Limited	-0.0530	0.0066	0.0000	[-0.066 : -0.04]
Profession	Sales Person	-0.0562	0.0059	0.0000	[-0.068 : -0.045]
Age	55	0.0053	0.0051	0.2995	[-0.0047 : 0.015]
Applications	5	0.1228	0.0057	0.0000	[0.11 : 0.13]
Stay	4 Years	0.0644	0.0054	0.0000	[0.054 : 0.075]
Shown	Second	-0.0325	0.0069	0.0000	[-0.046 : -0.019]

```
######### NO COUNTRY, IDEOLOGY variation linear
d.ideology ← d %>%
  left_join(select(I, id, `Right-wing ideology`)) %>%
  mutate(Ideology = ifelse(`Right-wing ideology` == 1, "Right-wing", "No right-wing")) %>%
  mutate(Ideology = fct_relevel(Ideology, c("No right-wing", "Right-wing")))

amce ← cj(
          id = ~id,
          by = ~ Ideology,
   data = d.ideology,
   formula = Decision ~ Nationality + Gender + Language + Profession + Age +
     Applications + Stay + Shown,
   estimate = "amce",
   )


tc ← "Estimated Average Marginal Component Effects. No country differences. Nationality preferences by Ide
tb ← amce %>%
  filter(!is.na(std.error)) %>%
  mutate(CI = paste0("[", signif(lower, 2), " : ", signif(upper, 2), "]")) %>%
  select(Ideology, Feature = feature, Level = level, `AMCE Estimate` = estimate, SE = std.error, `p-value`
  arrange(Feature, Ideology)


print(xtable(tb,
             caption = tc,
             digits = 4,
             label = "tab:amce_nocountry_ideology"),
```

```
  file = "tab-p7-amce_nocountry_ideology.tex",
  include.rownames = FALSE,
  caption.placement = "top",
  sanitize.text.function = identity)

mykbl(tb, tc, digits = 4)
```

Table 3.9: Estimated Average Marginal Component Effects. No country differences. Nationality preferences by Ideology.

Ideology	Feature	Level	AMCE Estimate	SE	p-value	CI
No right-wing	Nationality	French	0.0088	0.0053	0.0955	[-0.0015 : 0.019]
Right-wing	Nationality	French	0.0406	0.0147	0.0058	[0.012 : 0.069]
No right-wing	Gender	Male	-0.0437	0.0049	0.0000	[-0.053 : -0.034]
Right-wing	Gender	Male	-0.0072	0.0148	0.6259	[-0.036 : 0.022]
No right-wing	Language	Limited	-0.0451	0.0070	0.0000	[-0.059 : -0.031]
Right-wing	Language	Limited	-0.1121	0.0193	0.0000	[-0.15 : -0.074]
No right-wing	Profession	Sales Person	-0.0521	0.0063	0.0000	[-0.064 : -0.04]
Right-wing	Profession	Sales Person	-0.0878	0.0178	0.0000	[-0.12 : -0.053]
No right-wing	Age	55	0.0071	0.0054	0.1877	[-0.0034 : 0.018]
Right-wing	Age	55	-0.0087	0.0159	0.5831	[-0.04 : 0.022]
No right-wing	Applications	5	0.1263	0.0061	0.0000	[0.11 : 0.14]
Right-wing	Applications	5	0.0982	0.0158	0.0000	[0.067 : 0.13]
No right-wing	Stay	4 Years	0.0635	0.0057	0.0000	[0.052 : 0.075]
Right-wing	Stay	4 Years	0.0711	0.0162	0.0000	[0.039 : 0.1]
No right-wing	Shown	Second	-0.0326	0.0073	0.0000	[-0.047 : -0.018]
Right-wing	Shown	Second	-0.0319	0.0205	0.1197	[-0.072 : 0.0083]

```
######### COUNTRY VARIATIONS
amce.dk <- cj(
           id = ~id,
  data = d %>% filter(Country == "Denmark"),
  formula = Decision ~ Nationality + Gender + Language + Profession + Age + Applications + Stay + Shown, e
amce.ie <- cj(
           id = ~id,
  data = d %>% filter(Country == "Ireland"),
  formula = Decision ~ Nationality + Gender + Language + Profession + Age + Applications + Stay + Shown, e
amce.sp <- cj(
           id = ~id,
  data = d %>% filter(Country == "Spain"),
  formula = Decision ~ Nationality + Gender + Language + Profession + Age + Applications + Stay + Shown, e
amce.ch <- cj(
           id = ~id,
  data = d %>% filter(Country == "Switzerland"),
  formula = Decision ~ Nationality + Gender + Language + Profession + Age + Applications + Stay + Shown, e


amces <- bind_rows(
  amce.ie %>% mutate(Country = "Ireland", .before = 1),
  amce.dk %>% mutate(Country = "Denmark", .before = 1),
  amce.sp %>% mutate(Country = "Spain", .before = 1),
  amce.ch %>% mutate(Country = "Switzerland", .before = 1) )
```

```
tc ← "Estimated Average Marginal Component Effects. Country differences."
tb ← amces %>%
  filter(!is.na(std.error)) %>%
  mutate(CI = paste0("[", signif(lower, 2), " : ", signif(upper, 2), "]")) %>%
  select(Country, Feature = feature, Level = level, `AMCE Estimate` = estimate, SE = std.error, `p-value` =
  arrange(Feature, Country)


print(xtable(tb,
             caption = tc,
             digits = 4,
             label = "tab:amce_country"),
  file = "tab-p7-amce_country.tex",
  include.rownames = FALSE,
  caption.placement = "top",
  sanitize.text.function = identity)

mykbl(tb, tc, digits = 4)
```

Table 3.10: Estimated Average Marginal Component Effects. Country differences.

Country	Feature	Level	AMCE Estimate	SE	p-value	CI
Denmark	Nationality	French	0.0219	0.0094	0.0193	[0.0036 : 0.04]
Ireland	Nationality	French	0.0014	0.0099	0.8906	[-0.018 : 0.021]
Spain	Nationality	French	0.0204	0.0095	0.0320	[0.0018 : 0.039]
Switzerland	Nationality	French	0.0066	0.0109	0.5429	[-0.015 : 0.028]
Denmark	Gender	Male	-0.0366	0.0091	0.0001	[-0.054 : -0.019]
Ireland	Gender	Male	-0.0382	0.0089	0.0000	[-0.056 : -0.021]
Spain	Gender	Male	-0.0486	0.0089	0.0000	[-0.066 : -0.031]
Switzerland	Gender	Male	-0.0335	0.0107	0.0017	[-0.054 : -0.013]
Denmark	Language	Limited	-0.1066	0.0134	0.0000	[-0.13 : -0.08]
Ireland	Language	Limited	-0.0131	0.0127	0.3026	[-0.038 : 0.012]
Spain	Language	Limited	-0.0190	0.0130	0.1439	[-0.044 : 0.0065]
Switzerland	Language	Limited	-0.0747	0.0126	0.0000	[-0.1 : -0.05]
Denmark	Profession	Sales Person	-0.1037	0.0107	0.0000	[-0.12 : -0.083]
Ireland	Profession	Sales Person	-0.0867	0.0119	0.0000	[-0.11 : -0.063]
Spain	Profession	Sales Person	-0.0290	0.0109	0.0081	[-0.05 : -0.0075]
Switzerland	Profession	Sales Person	0.0070	0.0136	0.6053	[-0.02 : 0.034]
Denmark	Age	55	-0.0255	0.0092	0.0057	[-0.044 : -0.0074]
Ireland	Age	55	-0.0029	0.0102	0.7723	[-0.023 : 0.017]
Spain	Age	55	0.0393	0.0100	0.0001	[0.02 : 0.059]
Switzerland	Age	55	0.0111	0.0114	0.3284	[-0.011 : 0.033]
Denmark	Applications	5	0.1319	0.0111	0.0000	[0.11 : 0.15]
Ireland	Applications	5	0.1371	0.0112	0.0000	[0.12 : 0.16]
Spain	Applications	5	0.0862	0.0111	0.0000	[0.064 : 0.11]
Switzerland	Applications	5	0.1420	0.0123	0.0000	[0.12 : 0.17]
Denmark	Stay	4 Years	0.0563	0.0099	0.0000	[0.037 : 0.076]
Ireland	Stay	4 Years	0.0670	0.0113	0.0000	[0.045 : 0.089]
Spain	Stay	4 Years	0.0719	0.0102	0.0000	[0.052 : 0.092]
Switzerland	Stay	4 Years	0.0624	0.0118	0.0000	[0.039 : 0.086]
Denmark	Shown	Second	-0.0219	0.0127	0.0862	[-0.047 : 0.0031]
Ireland	Shown	Second	-0.0346	0.0143	0.0154	[-0.063 : -0.0066]
Spain	Shown	Second	-0.0403	0.0135	0.0028	[-0.067 : -0.014]
Switzerland	Shown	Second	-0.0349	0.0149	0.0194	[-0.064 : -0.0056]

3.8 *Provide AMCE distribution using HB*

We don't fully work with the full distribution, since the object contains the medians of the posteriors by individual.

```
tb ← ci.beta %>%
  mutate(prob.diff = plogis(median) - 0.5) %>%
  group_by(Feature) %>%
  summarize(AMCE = mean(prob.diff)) %>%
#  summarize(AMCE = mean(prob.diff),
#            SD = sd(prob.diff),
#            CI = paste0("[", signif(quantile(prob.diff, 0.025), 4), " : ", signif(quantile(prob.diff, 0.09
  ungroup()

print(xtable(tb,
             caption = "AMCE. No country differences.",
             digits = 4,
             label = "tab:hb_amce_country"),
  file = "tab-p7-hb_amce_nocountry.tex",
  include.rownames = FALSE,
  caption.placement = "top",
  sanitize.text.function = identity)


tb ← ci.beta %>%
  mutate(prob.diff = plogis(median) - 0.5) %>%
  left_join(select(I, id, `Right-wing ideology`)) %>%
  mutate(Ideology = ifelse(`Right-wing ideology` == 1, "Right-wing", "No right-wing")) %>%
  mutate(Ideology = fct_relevel(Ideology, c("No right-wing", "Right-wing"))) %>%
  group_by(Feature, Ideology) %>%
  summarize(AMCE = mean(prob.diff)) %>%
#  summarize(AMCE = mean(prob.diff),
#            SD = sd(prob.diff),
#            CI = paste0("[", signif(quantile(prob.diff, 0.025), 4), " : ", signif(quantile(prob.diff, 0.09
  ungroup() %>%
  arrange(Feature, Ideology)

print(xtable(tb,
             caption = "AMCE. No country differences. Ideology.",
             digits = 4,
             label = "tab:hb_amce_nocountry_ideology"),
  file = "tab-p7-hb_amce_nocountry_ideology.tex",
  include.rownames = FALSE,
  caption.placement = "top",
  sanitize.text.function = identity)


tb ← ci.beta %>%
  mutate(prob.diff = plogis(median) - 0.5) %>%
  group_by(Feature, Country) %>%
  summarize(AMCE = mean(prob.diff)) %>%
#  summarize(AMCE = mean(prob.diff),
#            SD = sd(prob.diff),
#            CI = paste0("[", signif(quantile(prob.diff, 0.025), 4), " : ", signif(quantile(prob.diff, 0.09
```

```
  ungroup() %>%
  arrange(Feature, Country)

print(xtable(tb,
             caption = "AMCE. Country differences.",
             digits = 4,
             label = "tab:hb_amce_country"),
  file = "tab-p7-hb_amce_country.tex",
  include.rownames = FALSE,
  caption.placement = "top",
  sanitize.text.function = identity)
```

3.9 *Do French profiles have an extra bonus for integration efforts*

```
set.seed(14718)
d.model ← ci.beta %>%
  filter(Feature %in% c("Nationality: French", "Language: Fluent", "Applications: 5")) %>%
  select(id, Feature, median, Country) %>%
  pivot_wider(names_from = Feature, values_from = median) %>%
  rename(`Nationality French` = `Nationality: French`,
         `Language Fluent` = `Language: Fluent`,
         `Applications 5` = `Applications: 5`)


m.language ← stan_glm(`Nationality French` ~ `Language Fluent`, data = d.model,
              family = gaussian())
m.applications ← stan_glm(`Nationality French` ~ `Applications 5`, data = d.model,
              family = gaussian())
m.language.applications ← stan_glm(`Nationality French` ~ `Language Fluent` + `Applications 5`, data = d.m
              family = gaussian())


modelsummary(list("Language" = m.language,
                  "Applications" = m.applications,
                  "Language + Applications" = m.language.applications),
             title = "Simple models on individual discriminations prefering French over Bulgarian profiles
             metrics = c("R2"), statistic = "conf.int") # RMSE

modelsummary(list("Language" = m.language,
                  "Applications" = m.applications,
                  "Language + Applications" = m.language.applications),
             title = "Simple linear regression models on individual discriminations prefering French over B
             escape = FALSE,
             metrics = "R2",
#             gof_map = c("nobs", "r.squared"),
             statistic = "conf.int",
  #output = "table-p7-hextra.html")
  output = "table-p7-hextra.docx")
```

Table 3.11: Simple models on individual discriminations prefering French over Bulgarian profiles based on integration efforts (language and/or applications).

	Language	Applications	Language + Applications
(Intercept)	0.147	0.212	0.275
	[0.091, 0.205]	[0.150, 0.275]	[0.213, 0.336]
‘Language Fluent‘	−0.125		−0.141
	[−0.155, −0.093]		[−0.173, −0.109]
‘Applications 5‘		−0.142	−0.161
		[−0.178, −0.106]	[−0.195, −0.127]
Num.Obs.	2403	2403	2403
R2	0.025	0.026	0.058
Log.Lik.	−4224.196	−4221.905	−4182.916

Programming environment

```
sessionInfo()
```

```
→ R version 4.5.2 (2025-10-31)
→ Platform: aarch64-unknown-linux-gnu
→ Running under: 'Gentoo Linux'
→
→ Matrix products: default
→ BLAS/LAPACK: FlexiBLAS OPENBLAS;  LAPACK version 3.12.0
→
→ locale:
→  [1] LC_CTYPE=ca_AD.UTF-8       LC_NUMERIC=C               LC_TIME=ca_AD.UTF-8
→  [4] LC_COLLATE=ca_AD.UTF-8     LC_MONETARY=ca_AD.UTF-8    LC_MESSAGES=ca_AD.UTF-8
→  [7] LC_PAPER=ca_AD.UTF-8       LC_NAME=C                  LC_ADDRESS=C
→ [10] LC_TELEPHONE=C             LC_MEASUREMENT=ca_AD.UTF-8 LC_IDENTIFICATION=C
→
→ time zone: Europe/Andorra
→ tzcode source: system (glibc)
→
→ attached base packages:
→ [1] parallel  grid      stats     graphics  grDevices utils     datasets  methods   base
→
→ other attached packages:
→  [1] xtable_1.8-4           cregg_0.4.0            rstan_2.32.7           StanHeaders_2.32.10
→  [5] modelsummary_2.5.0     broom.mixed_0.2.9.6    rstanarm_2.32.2        ggmcmc_1.5.1.2
→  [9] marginaleffects_0.31.0 brms_2.23.0            Rcpp_1.1.0             ggh4x_0.3.1
→ [13] ggridges_0.5.7         scales_1.4.0           stringr_1.6.0          GGally_2.4.0
→ [17] gridExtra_2.3          devtools_2.4.6         usethis_3.2.1          ggthemes_5.2.0
→ [21] colorspace_2.1-2       forcats_1.0.1          kableExtra_1.4.0       runjags_2.2.2-5
→ [25] rjags_4-17             coda_0.19-4.1          tufte_0.14.0           tikzDevice_0.12.6
→ [29] rmarkdown_2.30         knitr_1.51             ggplot2_4.0.1          tidyr_1.3.2
→ [33] dplyr_1.1.4            extrafont_0.20         colorout_1.2-2
→
→ loaded via a namespace (and not attached):
→   [1] fs_1.6.6              matrixStats_1.5.0     httr_1.4.7            RColorBrewer_1.1-3
→   [5] insight_1.4.4         tools_4.5.2           backports_1.5.0       R6_2.6.1
→   [9] DT_0.34.0             mgcv_1.9-3            withr_3.0.2           Brobdingnag_1.2-9
→  [13] cli_3.6.5             textshaping_1.0.4     performance_0.15.3    shinyjs_2.1.0
→  [17] sandwich_3.1-1        labeling_0.4.3        mvtnorm_1.3-3         S7_0.2.1
→  [21] readr_2.1.6           tables_0.9.33         proxy_0.4-29          QuickJSR_1.8.1
→  [25] systemfonts_1.3.1     svglite_2.2.2         dichromat_2.0-0.1     parallelly_1.46.0
```

```
→  [29] sessioninfo_1.2.3    readxl_1.4.5           rstudioapi_0.17.1      generics_0.1.4
→  [33] gtools_3.9.5         crosstalk_1.2.2        distributional_0.5.0   inline_0.3.21
→  [37] loo_2.9.0            Matrix_1.7-4           DescTools_0.99.60      abind_1.4-8
→  [41] lifecycle_1.0.4      multcomp_1.4-29        yaml_2.3.12            ggstance_0.3.7
→  [45] promises_1.5.0       miniUI_0.1.2           lattice_0.22-7         haven_2.5.5
→  [49] cowplot_1.2.0        magick_2.9.0           pillar_1.11.1          boot_1.3-32
→  [53] gld_2.6.8            estimability_1.5.1     shinystan_2.7.0        future.apply_1.20.1
→  [57] codetools_0.2-20     glue_1.8.0             V8_8.0.1               data.table_1.18.0
→  [61] remotes_2.5.0        vctrs_0.6.5            Rdpack_2.6.4           testthat_3.3.1
→  [65] cellranger_1.1.0     gtable_0.3.6           datawizard_1.3.0       cachem_1.1.0
→  [69] xfun_0.55            rbibutils_2.4          mime_0.13              survey_4.4-8
→  [73] reformulas_0.4.3     survival_3.8-3         filehash_2.4-6         shinythemes_1.2.0
→  [77] tinytex_0.58         ellipsis_0.3.2         TH.data_1.1-5          nlme_3.1-168
→  [81] xts_0.14.1           threejs_0.3.4          rprojroot_2.1.1        tensorA_0.36.2.1
→  [85] otel_0.2.0           DBI_1.2.3              pandoc_0.2.0           Exact_3.3
→  [89] tidyselect_1.2.1     emmeans_2.0.1          compiler_4.5.2         extrafontdb_1.1
→  [93] curl_7.0.0           tinytable_0.15.2       expm_1.0-0             xml2_1.5.1
→  [97] desc_1.4.3           bayestestR_0.17.0      colourpicker_1.3.0     posterior_1.6.1
→ [101] bookdown_0.46        checkmate_2.3.3        dygraphs_1.1.1.6       lmtest_0.9-40
→ [105] rappdirs_0.3.3       digest_0.6.39          minqa_1.2.8            htmltools_0.5.9
→ [109] pkgconfig_2.0.3      base64enc_0.1-3        lme4_1.1-38            fastmap_1.2.0
→ [113] rlang_1.1.6          htmlwidgets_1.6.4      shiny_1.12.1           farver_2.1.2
→ [117] zoo_1.8-15           jsonlite_2.0.0         magrittr_2.0.4         bayesplot_1.15.0
→ [121] parameters_0.28.3    furrr_0.3.1            stringi_1.8.7          rootSolve_1.8.2.4
→ [125] brio_1.1.5           MASS_7.3-65            plyr_1.8.9             pkgbuild_1.4.8
→ [129] ggstats_0.12.0       listenv_0.10.0         lmom_3.2               splines_4.5.2
→ [133] hms_1.1.4            igraph_2.2.1           markdown_2.0           effectsize_1.0.1
→ [137] reshape2_1.4.5       stats4_4.5.2           pkgload_1.4.1          rstantools_2.5.0
→ [141] evaluate_1.0.5       mitools_2.4            RcppParallel_5.1.11-1 nloptr_2.2.1
→ [145] tzdb_0.5.0           httpuv_1.6.16          Rttf2pt1_1.3.14        purrr_1.2.0
→ [149] future_1.68.0        broom_1.0.11           e1071_1.7-17           later_1.4.4
→ [153] viridisLite_0.4.2    class_7.3-23           tibble_3.3.0           memoise_2.0.1
→ [157] globals_0.18.0       bridgesampling_1.2-1
```
