# Supplementary material for: Identity- versus effort-based bureaucratic discrimination among mobile European Union citizens: Evidence from conjoint experiments
Source: Eur Union Polit. 2026 Mar 4;27(2):301–24. doi: 10.1177/14651165261423087 (PMC13218594; doi:10.1177/14651165261423087)
Supplement: sj-zip-3-eup-10.1177_14651165261423087 - Supplemental material for Identity- versus effort-based bureaucratic discrimination among mobile European Union citizens: Evidence from conjoint experiments [file sj-zip-3-eup-10.1177_14651165261423087.zip › README.pdf]

Replication material for "Identity versus effort-based bureaucratic discrimination among mobile European Union citizens: a conjoint experiment"

European Union Politics (2026)

Jana Gómez-Díaz, Eva Thomann, Anita Manatschal & Xavier Fernández-i-Marín

REQUIREMENTS

Software

The analysis is performed using R and JAGS:

- R: https://www.r-project.org/
- JAGS: http://mcmc-jags.sourceforge.net/

Both need to be installed in the system before installing `R`'s packages.

R packages

The file `install_packages.R` is provided for convenience to ensure that all necessary `R` packages are installed.

Note that for MacOS users, installing the `Cairo` package requires prior installation of https://xquartz.org.

Fonts

In order to replicate the figures as in the main text, the report explicitly calls for the "Source Sans Pro" font. If it is not available, change it for a font that is available in the system that is performing the replication, or leave it empty for the default.

The report also needs, in case of a PDF output, the "Source Code Pro" font.

Source Sans Pro and Source Code Pro are available via an open source license at https://www.fontsquirrel.com/fonts/source-sans-pro and https://www.fontsquirrel.com/fonts/source-code-pro, respectively.

INSTRUCTIONS

In order to generate the full report, simply compile `weave-p7.Rmd`, which is the main file containing calls to the rest of the files. This can be done either by invoking `make` in a POSIX operating system (GNU/Linux, BSD Unix or MacOS) from a command line, by running `run-p7.R` in R, or by "knitting" `weave-p7.Rmd` from R-Studio.

FILE DESCRIPTION

`weave-p7.Rmd`

Contains the R Markdown code for generating the whole report in both formats. It is empty of content, and only processes the aesthetical and functional details of the report. It calls child files (Rmd) that perform the different analyses.

The script takes a few minutes to generate in a CPU with an Apple M1 Max processor running on GNU/Linux with OpenBlas linear algebra libraries.

The result is a report (see `weave-p7.pdf`) containing the full analysis and replication, in PDF.

`weave-7.pdf`

This is the resulting file with the literate programming as obtained for a specific set of OS, software versions and linear algebra libraries employed.

`part-*Rmd`

RMarkdown files containing different parts of the analysis.

`load_packages.R`

List of R packages that are needed in all scripts.

Files with `*.RData` and directory `data/`

These files contain both the original and the pre-processed data.

`header.tex`

Used in the processing of the PDF output mostly to define fonts to use.

`install_packages.R`

In case R packages are missing, run this in R to install them.

NOTES ON REPLICATION

The exact numbers that appear in the tables have been generated using the details at the end of the report, with the output of `sessionInfo()` in `R`. But software changes, evolves, and default options may no longer be the same in future versions or in different computer setups.

In addition, using Bayesian inference methods and MCMC involves a stochastic process that relies heavily on the computer's capacity to produce random (or pseudo-random) numbers. While setting seeds ensures this, there is nothing that fixes that computers will use exactly the same default algorithm to produce

random numbers, specially when different versions of the software are combined, or when different software platforms (GNU/Linux, Windows, MacOS, mostly) and linear algebra software (in this specific case, BLAS libraries).

Therefore, the concrete and exact numbers reported, specially at certain degree of precision, can be different from the numbers retrieved using other software combinations.
